# Engineer chimeric Cas9 to expand PAM recognition based on evolutionary information

**DOI:** 10.1038/s41467-019-08395-8

**Published:** 2019-02-04

**Authors:** Dacheng Ma, Zhimeng Xu, Zhaoyu Zhang, Xi Chen, Xiangzhi Zeng, Yiyang Zhang, Tingyue Deng, Mengfei Ren, Zheng Sun, Rui Jiang, Zhen Xie

**Affiliations:** MOE Key Laboratory of Bioinformatics and Bioinformatics Division, Center for Synthetic and System Biology, Department of Automation, Beijing National Research Center for Information Science and Technology, Tsinghua University, Beijing, 100084 China

## Abstract

Although Cas9 nucleases are remarkably diverse in microorganisms, the range of genomic sequences targetable by a CRISPR/Cas9 system is restricted by the requirement of a short protospacer adjacent motif (PAM) at the target site. Here, we generate a group of chimeric Cas9 (cCas9) variants by replacing the key region in the PAM interaction (PI) domain of *Staphylococcus aureus* Cas9 (SaCas9) with the corresponding region in a panel of SaCas9 orthologs. By using a functional assay at target sites with different nucleotide recombinations at PAM position 3–6, we identify several cCas9 variants with expanded recognition capability at NNVRRN, NNVACT, NNVATG, NNVATT, NNVGCT, NNVGTG, and NNVGTT PAM sequences. In summary, we provide a panel of cCas9 variants accessible up to 1/4 of all the possible genomic targets in mammalian cells.

## Abstract (web summary)

The genomic locations that can be targeted for editing by CRISPR are limited by the presence of the nuclease-specific PAM sequence. Here, the authors show PAM recognition can be expanded by replacing the key region in the PAM interaction domain of SaCas9 with the corresponding region of SaCas9 orthologs.

## Introduction

CRISPR (clustered regularly interspaced short palindromic repeats)-Cas (CRISPR-associated) systems work as the prokaryotic adaptive immune systems that provide protection against infection^1^. The CRISPR system has been found in half of all sequenced bacterial genomes and nearly all archaeal genomes, and the CRISPR nucleases are highly diverse^2^. However, only several CRISPR nucleases are functional in mammalian cells so far. Several Cas9 orthologs from microbial type II CRISPR systems have been widely applied for targeted gene and base editing, transcription modulations, and epigenetic modifications in the mammalian genome^3–9^. Targeting of a specific genomic site (protospacer) is programmed by base-pairing with a chimeric guide RNA (gRNA) bound to the Cas9 endonuclease^10,11^. In addition, a short protospacer adjacent motif (PAM) is required for target recognition by the Cas9:gRNA complex, which significantly restricts the range of genomic sequences that are targetable by the CRISPR/Cas9 system^3,10^.

Recent structural studies of *Streptococcus pyogenes* Cas9 (SpCas9) and *Staphylococcus aureus* (SaCas9) elucidated the molecular mechanisms underlying PAM recognition, suggesting that multiple amino acid substitutions in the PAM interaction (PI) domain are often required to induce sufficient structural rearrangement to accommodate the altered PAM^12–16^. However, searching appropriate combinations of multiple amino acid substitutions for a specific PAM based on Cas9 structural information demands a daunting computational power. Several approaches, including error-prone PCR based evolution, phage-assisted continuous evolution (PACE), and structure-guided mutagenesis combined with bacterial-based negative and positive selection systems, have been successfully used to broaden the PAM compatibility by introducing random mutations in the PI domain or the entire coding region of CRISPR nucleases^17–20^. Nevertheless, these screening methods are still time-consuming, which demands the development of alternative strategies to generate Cas9 variants with altered PAM recognition compatibility.

**Fig. 1:**
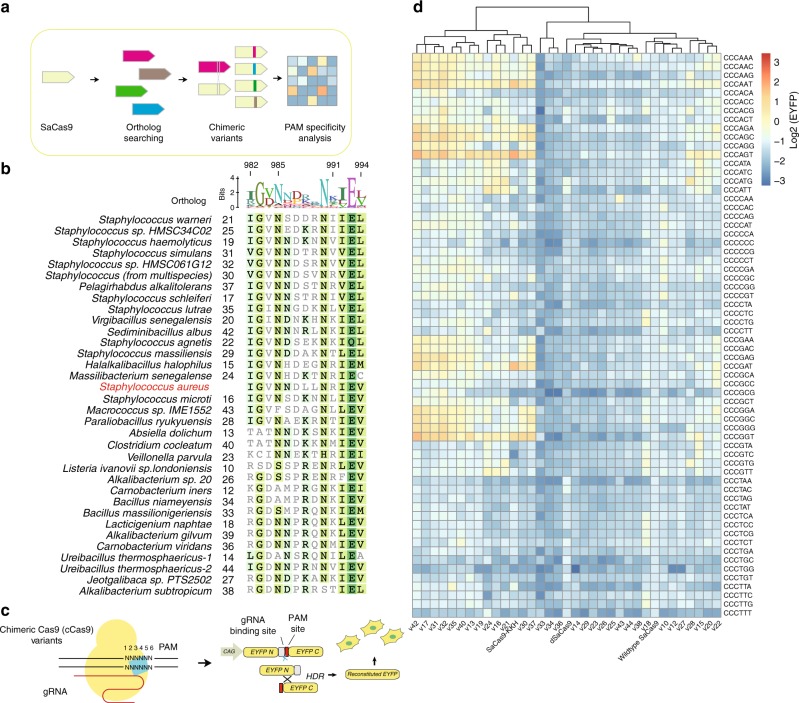
Chimeric Cas9 with expanded PAM specificities. **a** Design strategy for chimeric Cas9 (cCas9). **b** Sequence alignment of the 982–994 peptide fragment of orthologs. **c** Schematic of the EYFP reconstitution assay that was used to evaluate the cleavage activity of cCas9 variants at target sites containing indicated PAM sequences. **d** Data are shown as relative fluorescence intensity of EYFP measured by using flow cytometer 3 days after transfection into HEK293FT cells (*n* = 1). Source data are provided as a Source Data file

Here, we focus on SaCas9 engineering because SaCas9 is a compact Cas9 ortholog suitable for viral delivery for biomedical applications and SaCas9 displays a comparable activity to SpCas9 in mammalian cells^21,22^. The wild-type SaCas9 requires an NNGRRN (R = A or G) PAM with a preference for a T base at the 6th position of the PAM^21^. The structural study revealed that Arginine at position 1015 (R1015) forms bidentate hydrogen bonds with the G base at the 3rd position of the PAM^14^. A SaCas9 variant with E782K/N968K/R1015H triple mutations (SaCas9-KKH variant) has been identified to recognize an NNNRRT PAM^18^. Three amino acids (N985, N986, and R991), that lie in the key region of the PI domain, bind to the 4th and 5th bases of the PAM via direct and water-mediated hydrogen bonds^14^. Cas9 nucleases are remarkably diverse in microorganisms^23^, and many Cas9 nuclease orthologs recognize different PAM sequences^21,24,25^. Recently *Streptococcus* *canis* (ScCas9) with high similarity to SpCas9 has been found with minimal PAM restriction^26^. We hypothesize that replacing this key region in the PI domain of small SaCas9-KKH with the corresponding region of SaCas9 orthologs could likely result in a mammalian-compatible and compact chimeric Cas9 with expanded PAM compatibility (Fig. 1a).

## Results

### Description of PAM preference of chimeric SaCas9s

First, we searched for SaCas9 orthologs in the NCBI database by performing a BLAST analysis with the full-length amino acid sequences of SaCas9. We found 33 SaCas9 orthologs, including 11 orthologs identified in *Staphylococcus* species that showed a close homology to SaCas9 (Supplementary Fig. 1). We found that the crucial 13-aa region from 982 to 994 involved in binding to the 4th and 5th bases of the PAM was conserved in general (Fig. 1b)^14^. Besides, the key regions of ortholog 18 and 39 were identical to each other. Interestingly, the amino acid residues at position 986 and particularly that at position 991 were highly diverse, suggesting that these orthologs may recognize different PAM sequences (Fig. 1b). In addition, the residues at both N-terminal and C-terminal anchors of the crucial 13-aa region were highly conserved, which may help to accommodate the structural changes in the chimeric variants (Fig. 1b). Then, we generated 32 unique chimeric Cas9 (cCas9) variants by replacing this crucial 13-aa region in SaCas9-KKH with that region in SaCas9 orthologs.

We and other groups have shown that altering the 3rd or 4th U in the first stem-loop of the gRNA scaffold to disrupt the putative “UUUU” terminator sequences for Polymerase III can enhance Cas9 activity, likely due to the increased gRNA expression level^27–29^. Interestingly, we found the crRNA direct repeat region of all of the SaCas9 orthologs was highly diverse except for the first conserved 6-nt at the 5'-end, which may have evolved to avoid self-cleavage by its own Cas9 nucleases (Supplementary Fig. 2a). To prevent gRNA self-targeting when testing SaCas9 activity, we generated an optimized gRNA-2 by mutating the 2nd U to C (Supplementary Fig. 2b). By using a previous EYFP fluorescence reconstitution assay in HEK293FT cells^8^ (Fig. 1c), we compared the PAM preference of SaCas9-KKH directed by either the original gRNA or optimized gRNA-2 after transfection into HEK293FT cells for 3 days (Supplementary Fig. 2c). Since the SaCas9-KKH recognizes NNNRRT PAM sites and the 13-aa region is only responsible for the contact with 4th to 6th position of the PAM sequences, we arbitrarily assigned triple cytosines at the first to third PAM positions, and tested the PAM recognition preference to all 64 different sequences varying at PAM position 4, 5, and 6. Consistently, SaCas9-KKH with the wild-type gRNA scaffold showed a strong activity at the CCCRRT PAMs and a weak activity at the CCCGGA, CCCGGC, and CCCAGC PAMs. When directed by the optimized gRNA scaffold, SaCas9-KKH retained the high activity at the CCCRRT PAMs and displayed a weak activity at the CCCATT, CCCCGT, and most of the CCCRRV (V = A, C, G) PAMs (Supplementary Fig. 2c, d). The wild-type SaCas9 requires guanine at the third PAM position^21^. To investigate whether the optimized gRNA would also be suitable for the wild-type SaCas9, we further compared the activity of SaCas9 and SaCas9-KKH at CCGRRN with a guanine at the third position and CCCRRN PAMs with a cytosine at the third position, guided with wild-type gRNA or optimized gRNA-2. Similarly, we observed increased activity at CCGRRV PAMs by SaCas9 and CCSRRV PAMs (S = C or G) by SaCas9-KKH when directed with the optimized gRNA-2 (Supplementary Fig. 2e). Therefore, we used the optimized gRNA-2 in the rest of the study.

**Fig. 2:**
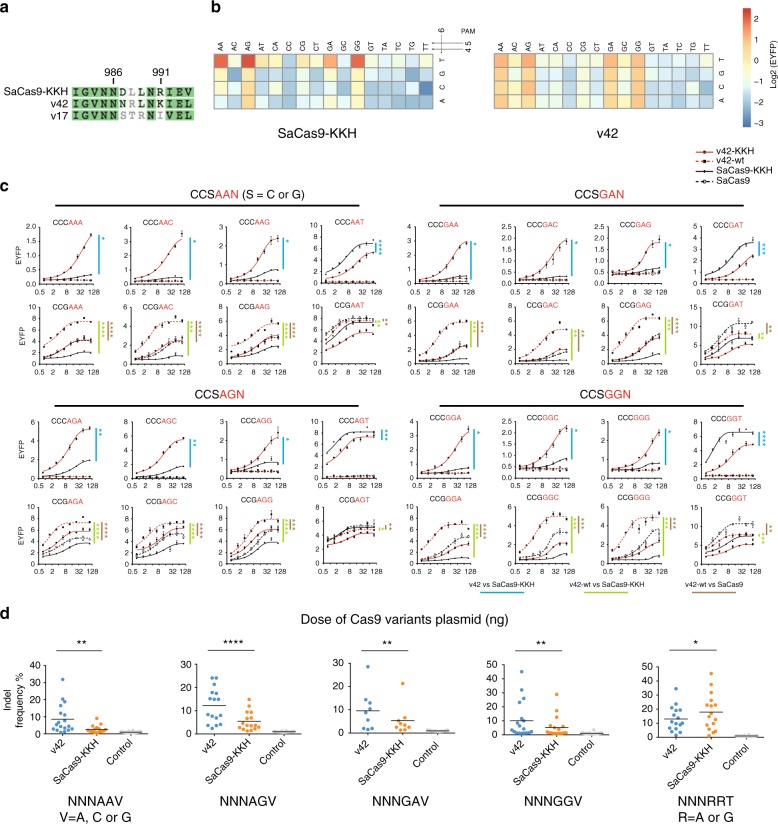
Characterization of PAM recognition of cCas9 v42. **a** Sequence alignment of the key 13-aa region of the PI domain in SaCas9-KKH, cCas9 v42, and v17. **b** Functional characterization of SaCas9-KKH and cCas9 v42 at CCCNNN PAMs. Schematic of the EYFP reconstitution assay is shown in Fig. 1c. Data indicated the mean of three independent biological replicates. **c** Functional comparison of v42-KKH, v42-wild-type (v42-wt), SaCas9 and SaCas9-KKH at CCGRRN and CCCRRN across eight plasmid doses by using the EYFP reconstitution assay. Data indicated the mean ± s.e.m. (*n* = 3 independent biological replicates). The relative EYFP fluorescence intensity (*y*-axis) was measured by FACS. **d** Indel frequencies induced by v42 or SaCas9-KKH at sites with NNNRRN PAMs measured by next-generation sequencing. Each point represents the mean of one endogenous site (*n* = 3 independent biological replicates). The black line indicates the mean of all targets. **c** and **d** *P < 0.05 (paired *t*-test, two-tailed); **P < 0.01 (paired *t*-test, two-tailed); ***P < 0.001 (paired *t*-test, two-tailed); ****P < 0.0001 (paired *t*-test, two-tailed). Source data are provided as a Source Data file

We evaluated the DNA cleavage efficiency of 32 cCas9 variants at all 64 different CCCNNN PAMs after transfection into HEK293FT cells for 3 days. We found that several cCas9 variants displayed a different PAM recognition pattern from SaCas9-KKH (Fig. 1d). We selected cCas9 v42 for further analysis because v42 displayed an enhanced activity at the CCCRRV PAMs compared to the SaCas9-KKH (Figs. 1d and  2). Sequence alignment showed that the key 13-aa region of SaCas9-KKH differed from that of v42 ortholog by three amino acid residues (Fig. 2a). After introducing mutations in a stepwise fashion, we found that R991K mutation reduced the preference of SaCas9-KKH to thymidine at the 6th PAM position, and R991K/D987N double mutations further relaxed the PAM specificity (Supplementary Fig. 3).

### Expanded PAM preference at sites with NNVRRV PAMs

Since the wild-type SaCas9 requires the guanine at the third position in the PAM and has better activity at sites of NNGRRV PAMs than SaCas9-KKH^18^, we further fused the 13-aa region of v42 to the wild-type SaCas9 to generate the cCas9 v42-wild-type (v42-wt). In addition, since the three-dimensional (3D) structure suggests that SaCas9 protein does not make direct contact with the first two nucleotides in the PAM sequences^14^, we arbitararily assigned double cytosines at the first two PAM positions in the experimental set-up. To characterize the cleavage activity of the cCas9 v42 and cCas9 v42-wild-type (v42-wt) at either CCGRRN or CCCRRN PAM, we transfected HEK293FT cells with four-plasmid mixtures, in which one plasmid expressed EBFP as the transfection marker, one expressed the optimized gRNA-2, one expressed the reporter EYFP gene and the fourth plasmid expressed the corresponding Cas9 variants. Notably, cCas9 v42 showed the highest activity at the CCCRRV (V = A, C and G) PAMs over a range of plasmid doses (Fig. 2c). As expected, we observed only basal level activity of cCas9 v42-wild-type at CCCRRN PAMs (Fig. 2c). By contrast, v42-wild-type showed the highest activity at the CCGRRV PAMs (Fig. 2c). Furthermore, SaCas9 and SaCas9-KKH showed the highest efficiency at the CCGRRT and CCCRRT, respectively (Fig. 2c). In addition, we demonstrated that cCas9 v42 displayed a comparable activity at the CCARRN PAMs, but only showed a weak activity at the CCTRRN PAMs (Supplementary Fig. 4). Collectively, these results suggested that cCas9 v42 had an expanded PAM recognition at the NNVRRV PAMs and a slightly increased activity at NNTRRV PAMs compared to SaCas9-KKH, with the assumption that the first two nucleotides can be either A, C, G, and T^14^.

To demonstrate that the v42 variant enables targeting of human endogenous sites in NNNRRV PAMs most of which currently cannot be efficiently recognized by SaCas9-KKH, we tested the v42 activity on 77 different endogenous gene target sites with a panel of NNNRRN PAMs with a lower plasmid dosage of 25 ng per transfection experiment. In general, cCas9 v42 displayed a higher activity at NNNRRV PAMs and a comparable activity at NNNRRT PAMs (Fig. 2d, Supplementary Fig. 5a, and Table 1). Sequence logo derived from sites with more than 5% indel frequency by v42 revealed preference of A, C, and G at the third position and no strong preference at the first two positions (Supplementary Fig. 5b), which were consistent with our reporter assay (Fig. 2c and Supplementary Fig. 4) and a previous report that SaCas9 does not make direct contact with the first two nucleotides in the PAM sequence^14,18^. To further examine whether the KKH trimutation in the chimeric Cas9 indeed expanded the third position of PAM, we selected 33 different endogenous gene target sites containing NNVRRN PAMs (V = A, C or G). As expected, only the cCas9 v42 showed efficient activity with a mean mutagenesis frequency above 10% across all of the sites, but v42-wild-type, SaCas9 and SaCas9-KKH displayed only basal levels of indels at the NNARRN PAMs and low activity with a mean indel frequency <6% at the NNCRRN PAMs (Supplementary Fig. 6 and Supplementary Table 2). Consistently, we also observed that nuclease inactivated cCas9 v42 fused with the gene activation domain VPR induced a 3 – 7-fold increase of *IL1RN* gene expression level when targeting the endogenous sites containing NNNRRV PAMs in the *IL1RN* promoter region, but resulted in a comparable *IL1RN* gene expression level when targeting the endogenous sites with NNNRRT PAMs (Supplementary Fig. 7).

Interestingly, the cCas9 v17 with either an I991K or I991L mutation (cCas9 v17-K and v17-L) expanded the activity on targets containing CCCRRN PAMs (Supplementary Fig. 8a). To compare the activity of v17-L, v42, and SaCas9-KKH when targeting endogenous target sites in HEK293FT cells, we performed the deep sequencing analysis on the indel frequency at 37 different endogenous target sites with NNNRRV PAMs. We observed that v17-L displayed about half of the sites showing higher than 5% indels with a mean mutagenesis frequency of 9.5% (Supplementary Fig. 8b and Supplementary Table 3).

### Expanded PAM preference at sites with non-NNNRRN PAMs

**Fig. 3:**
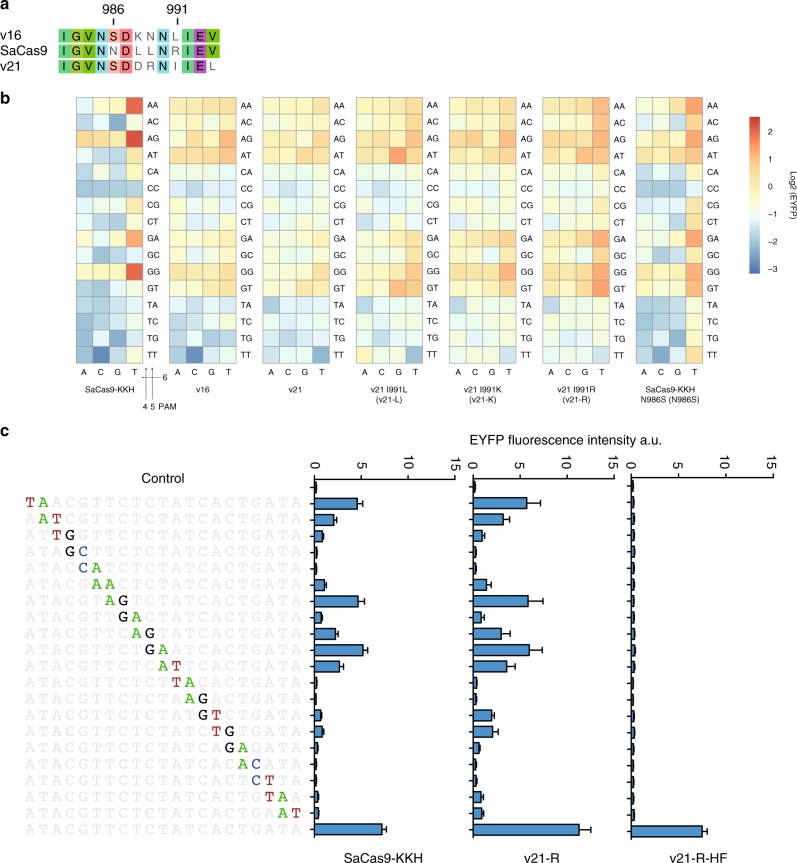
Variants with altered PAM preference and higher specificities. **a** Sequence alignment of the key 13-aa region of the PI domain in SaCas9-KKH (KKH), cCas9 v16 and v21. **b** Functional characterization of SaCas9 variants at CCCNNN PAMs. Schematic of the EYFP reconstitution assay is shown in Fig. 1c. Data indicated the mean (*n* = 3 independent biologic replicates) activities of indicated cCas9 variants and SaCas9-KKH. **c** Cleavage activities of SaCas9-KKH (KKH), cCas9 v21-R, and v21-R-HF (containing R499A, Q500K, R654A, and G655R mutations) at on-target and off-targets with dinucleotide mutations were evaluated by using the EYFP reconstitution assay. All targets contained a CCCAGT PAM. Bars represented the mean (± s.e.m., *n* = 3) of EYFP fluorescent intensity measured by using flow cytometer 3 days after transfection into HEK293FT cells. Source data are provided as a Source Data file

Next, we selected cCas9 v16 and v21 for further analysis because the residues at both position 986 and 991 in cCas9 v16 and v21 differed from those in the SaCas9-KKH (Fig. 3a), and these two variants showed a different PAM recognition pattern compared to the SaCas9-KKH (Figs. 1d and 3b). We mutated the Isoleucine (I) at position 991 to Leucine (L), Lysine (K) or Arginine (R), which were among the top residues that frequently appeared at position 991 in all 33 SaCas9 orthologs, generating cCas9 v21 I991L (v21-L), v21 I991K (v21-K) and v21 I991R (v21-R) variants (Fig. 3b). We found that these mutations increased the activity of cCas9 v21 on targets containing several non-NNNRRN-expanded PAM sequences, including CCCACT, CCCATG, CCCATT, CCCGCT, CCCGTG and CCCGTT (Fig. 3b). Interestingly, v16 and v21 shared the same Serine (S) residue at 986 position, which was different from the Asparagine (N) at the same position in SaCas9. We showed that the SaCas9 variant with N986S mutation also expanded the PAM specificity of SaCas9-KKH with a similar PAM recognition pattern compared to cCas9 v16 and v21 variants (Fig. 3b). Similar to the cCas9 v42 variant, we confirmed that the cCas9 v21-R variant showed efficient activities at six different PAMs with the adenosine, guanine, or cytosine but not thymidine at the third position (Supplementary Fig. 9).

To evaluate the off-target activity of cCas9 variants, we generated a panel of gRNAs with dinucleotide mutations to target a reporter gene containing the CCCAGT PAM (Fig. 3c). Although cCas9 v21-R showed increased activity on the on-target, it had a stronger activity on the off-target with dinucleotide mutations compared with the SaCas9-KKH (Fig. 3c). Recently, it has been reported that neutralization of positively charged residues positioned proximally to the nontarget strand groove promotes rehybridization between the target and nontarget mutations, resulting in mutant SpCas9 and SaCas9 with improved specificity^30^. Accordingly, we engineered the cCas9 v21-R with R499A, Q500K, R654A, and G655R mutations (v21-R-HF). We demonstrated that the cCas9 v21-R-HF retained a similar activity at the on-target but a negligible activity at the off-targets with dinucleotide mutations compared to SaCas9-KKH, although the gene editing activity of cCas9 v21-R-HF on the on-target reduced to ~65% compared with the cCas9 v21-R (Fig. 3). As shown in Supplementary Fig. 10, v21-R-HF displayed significantly decreased rates of mutagenesis at two out of three endogenous off-target sites containing one point mutation in the spacer sequences when directed by either wild-type gRNA or optimized gRNA-2 scaffold.

**Fig. 4:**
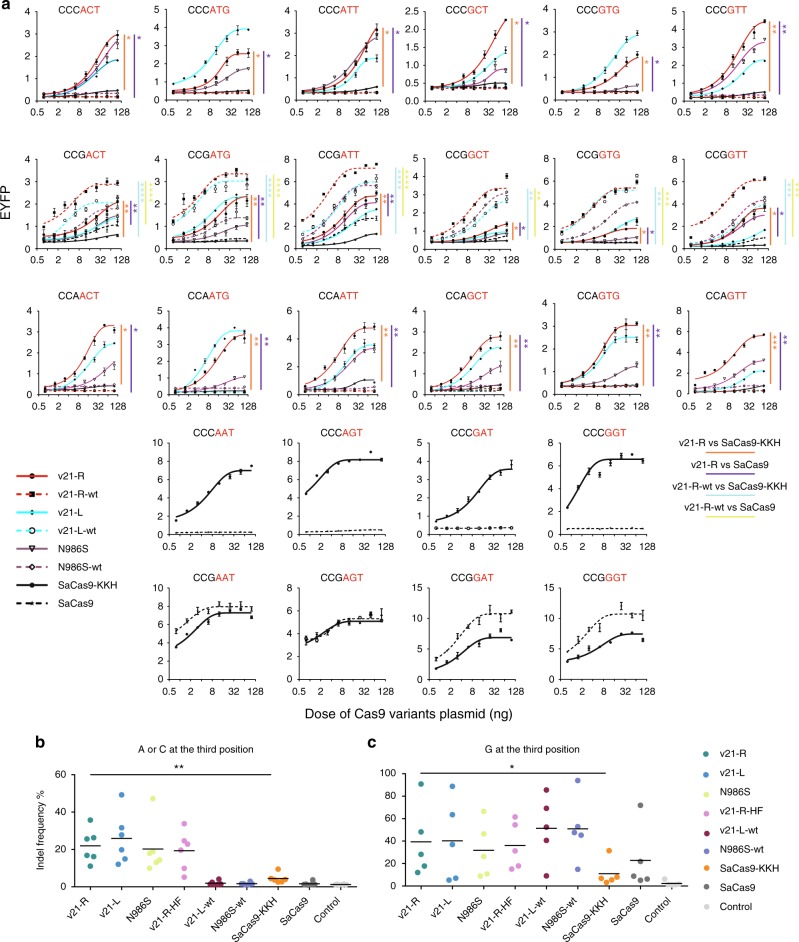
Comparison of chimeric cCas9 variants at non-NNNRRN PAMs. **a** Direct comparison of v21-R, v21-L, N986S, v21-R-wt, v21-L-wt, N986S-wt, SaCas9, and SaCas9-KKH with eight plasmids doses across CCVACT, CCVGTG, CCVGTT, CCVGCT, CCVATT, CCVATG PAMs (V = A, C or G) by EYFP reconstitution assay. (*n* = 3) Error bars, s.e.m. The relative EYFP fluorescence intensity (*y*-axis) is measured by FACS. **b** Indel frequency induced by indicated variant at the sites where the third position of PAM is A or C. **c** Indel frequency induced by indicated variant at the sites where the third position of PAM is G. Each point represents the mean of one endogenous site (*n* = 3 independent biological replicates). The black line indicates the mean activities at all targets. **a**, **b** and **c** *P < 0.05 (paired *t*-test, two-tailed); **P < 0.01 (paired *t*-test, two-tailed); ***P < 0.001 (paired *t*-test, two-tailed); ****P < 0.0001 (paired *t*-test, two-tailed). Source data are provided as a Source Data file

To further examine the nuclease activity of chimeric Cas9 variants at these 6 PAMs in a dose experiment, we fused the 13-aa of v21-R, v21-L, N986S into wild-type SaCas9 (v21-R-wt, v21-L-wt, N986S-wt), and tested the activity of these variants at 18 different PAMs with a guanine, a cytosine or an adenine at the third PAM position. By using the fluorescent reporter assay (Fig. 1c), we observed that v21-L and v21-R showed high activities at CCMACT, CCMATG, CCMATT, CCMGCT, CCMGTG, and CCMGTT PAMs (M = A or C), while N986S displayed relatively high efficiencies at CCMGTT, CCMATT, and CCMACT PAMs (Fig. 4a). Similarly, the cCas9 variants with the wild-type SaCas9 scaffold were highly active at PAM sites with a guanine at the third position (Fig. 4). Then, we selected 11 endogenous target sites with the non-NNNRRN PAMs and assayed the activities of different cCas9 variants by using the deep-sequencing analysis. We observed that the average indel frequencies induced by using v21-R, v21-L, N986S and v21-R-HF were >10% when targeting endogenous sites with six different PAMs (Fig. 4b, c and Supplementary Tables 4 and 5). Furthermore, chimeric Cas9 variants with the scaffold of either wild-type SaCas9 or SaCas9-KKH displayed higher level of indels than SaCas9-KKH at sites of non-NNNRRN PAMs with a guanine at the third position (Fig. 4c and Supplementary Table 5). In addition, we also confirmed that both v21-L and v21-R efficiently induced indels when targeting endogenous sites with NNVRRN PAMs (Supplementary Fig. 11). Altogether, these results showed that cCas9 v21-R had an expanded PAM recognition compared to SaCas9 and SaCas9-KKH.

## Discussion

In this study, we developed a strategy to engineer SaCas9 variants with altered PAM recognition specificity by swapping the key region in the PI domain in SaCas9 orthologs (Fig. 1). We identified several cCas9 v42 and v17-L variants with expanded DNA cleavage activities at NNVRRN PAMs, along with multiple cCas9 v16 and v21 derived variants that can efficiently target sites with NNVACT, NNVATG, NNVATT, NNVGCT, NNVGTG, and NNVGTT PAM (Figs. 2–4). In addition, we demonstrated that the v42-wt based on the wild-type SaCas9 scaffold showed a higher activity at NNGRRV PAMs than the wild-type SaCas9 by using the fluorescent reporter assay. Similarly, the v21-R-wt and v21-L-wt based on the wild-type SaCas9 scaffold also displayed an enhanced activity at NNGACT, NNGATG, NNGATT, NNGGCT, NNGGTG, and NNGGTT PAMs compared to the wild-type SaCas9. It will be interesting to reveal the 3D structure of these cCas9 variants to understand the molecular mechanism for the altered PAM recognition. In addition, directed evolution screening and structure-guided mutagenesis based on these cCas9 variants may further improve the DNA cleavage activities at targets containing the expanded PAM sequences. It is intriguing that although the v42, v17-L, SaCas9-KKH R991K, and SaCas9-KKH R991K/D987N showed expanded activities at NNVRRV PAMs, these variants displayed decreased activities on NNVRRT PAMs, which is consistent with the previous report that the SaCas9-KKH showed decreased activities at NNGRRT PAMs^18^. One explanation is that sufficient PAM binding activity of SaCas9 nucleases may be required to initiate strong gene editing activities and relaxed PAM binding activity of SaCas9 nucleases results in reduced DNA cleavage activity. Further studies are necessary to fully understand the functional relationship between the PAM recognition and the SaCas9 nuclease activity. Although cCas9 variants displayed expanded ability in non-canonical sites, we found these developed cCas9 variants showed decreased activities in previous canonical PAMs of SaCas9-KKH. For example, the cCas9 v42 generates lower efficiency in sites with NNNRRT PAMs, in line with the previous finding that SaCas9-KKH is weaker than wild-type SaCas9 at PAM sites with guanine at the third position^18^. In general, one of possible explanation is that the specific recognition and broad recognition is contradicted with each other due to the nature of protein–DNA interaction.

In summary, we provided a panel of cCas9 variants that are accessible up to 1/4 of all of the PAM sequences with a compact size suitable for viral delivery in mammalian cells, which will be valuable for biomedical applications that require precise Cas9 positioning. This chimeric strategy based on the evolutionary information may also be insightful to engineer Cas9 proteins for other functional purposes, such as low immugeneticity, high-fidelity and functional compatibility in mammalian cells.

## Methods

### Reagents and enzymes

Restriction endonuclease, polynucleotide kinase (PNK), T4 DNA ligase, and Q5 High-Fidelity DNA Polymerase were purchased from New England Biolabs. Oligonucleotides were synthesized by Ruibiotech.

### Plasmid DNA constructs

The gRNA sequences and associated primers are listed in Supplementary Tables 6–12. The DNA sequences of the constructs are listed in Supplementary Table 13.

### Cell culture and transfection

The HEK293FT cell line was purchased from Life Technologies. HEK293FT cells were cultured in high-glucose DMEM complete media (Dulbecco’s modified Eagle’s medium (DMEM), 4.5 g/L glucose, 0.045 unit/mL of penicillin, 0.045 g/mL streptomycin, and 10% FBS (Life Technologies)) at 37 °C, 100% humidity, and 5% CO_2_. One day before transfection, ∼1.2 × 10^5^ HEK293FT cells in 0.5 mL of high-glucose DMEM complete media were seeded into each well of 96-well plastic plates (Falcon). Shortly before transfection, the medium was replaced with fresh DMEM complete media. The transfection experiments were performed by using EpFect transfection reagent (SyngenTech) by following the manufacturer’s protocol. Each transfection experiment was independently repeated.

For the EYFP reconstitution reporter assay, 50 ng plasmid DNA encoding Cas9 variant if not emphasized particularly, 50 ng transfection control plasmid DNA (pB018 CAG:TagBFP) that constitutively express TagBFP, 50 ng plasmid DNA encoding the gRNA with a spacer sequence “ATACGTTCTCTATCACTGATA”, and 50 ng plasmid DNA encoding the inactive EYFP reporter gene that can be reconstituted via homologous recombination after DNA cleavage were mixed and cotransfected into each well of a 96-well plate. For the endogeneous editing assay, in Fig. 2d and Supplementary Fig 8b, 25 ng Cas9 variant plasmid DNA, 25 ng gRNA plasmid DNA, and 50 ng transfection control plasmid DNA (hEF1α:EYFP-2A-puro) that encoded a constitutively expressed puromycin gene were mixed and cotransfected into each well of a 96-well plate. In Fig. 4b, c, 100 ng Cas9 variant plasmid DNA, 100 ng gRNA plasmid DNA and 50 ng transfection control plasmid DNA were cotransfected into each well of a 96-well plate. Unless otherwise stated, 50 ng Cas9 variants plasmid DNA, 50 ng gRNA plasmid DNA, and 50 ng transfection control plasmid DNA were cotransfected into each well of a 96-well plate. To select transfected cells, puromycin (Invitrogen) was added at a final concentration of 10 µg/mL after 1 day, and fresh DMEM complete media were replaced after 4 days.

For each *IL1RN* gene activation assay in Supplementary Fig. 3b, 50 ng plasmid DNA encoding Cas9 fused to the transactivation domain VPR and 12.5 ng of four different plasmids encoding gRNAs with the same PAM sites were mixed and cotransfected into HEK293FT cells in each well of a 96-well plate.

### Flow cytometry

Cells were trypsinized 3 days after transfection and centrifuged at 300 × *g* for 7 min at 4 °C. The supernatant was removed, and the cells were resuspended in 1 × phosphate-buffered saline (PBS) that did not contain calcium or magnesium. Fortessa flow analyzer (BD Biosciences) was used for fluorescence-activated cell sorting (FACS) analysis with the following settings: EBFP2 was measured using a 405 nm laser and a 450/50 filter with a photomultiplier tube (PMT) set at 275 V. The EYFP was measured with a 488 nm laser and a 530/30 filter using a PMT set at 270 V. For each sample, ∼2 × 10^4^ to ∼3 × 10^4^ cell events were collected. The relative fluorescence intensity of EYFP (EYFP fluorescence intensity a.u.) was defined as the average fluorescence intensity of EYFP divided by the average fluorescence intensity of the internal control EBFP2 fluorescence.

### RNA purification and quantitative PCR

Total RNA from HEK293FT cells was extracted with Trizol reagent (Life Technology). For each sample, 500 ng total RNA was reversed transcripted by ReverTra Ace qPCR RT Master Mix with gDNA Remover Kit (TOYOBO), and 1 μL of cDNA was used for each qPCR reaction, using 2× EvaGreen Master Mix (Syngentech). The quantitative reverse transcription polymerase chain reaction (qRT-PCR) reaction was run and analyzed in the Light cycler 480 II (Roche) with all target gene expression levels normalized to β-actin mRNA levels. The primers are listed in Supplementary Table 14.

### Mutation quantification

All of the indels frequencies were measured by targeted next-generation sequencing (Illumina). Appropriately 5 days post-transfection, cells were harvested and lysed by lysis buffer (NP40 0.45% and 10 mM Tris-HCl, pH8.3) followed by 58 °C for 180 min and 95 °C for 10 min. Amplicons were generated by three rounds of nested PCR to add the illumina adaptor sequence. After filtering, Reads with mutation is defined by mismatch within a 48-bp window around the cleavage site. Indel frequency is counted by reads with mutation divided by total reads.

### Reporting summary

Further information on experimental design is available in the Nature Research Reporting Summary linked to this article.

## Supplementary information

Supplementary Information

Peer Review File

Description of Additional Supplementary Files

Source Data

Reporting Summary

## Data Availability

Next-generation sequencing data for detection indel frequency of the specific sites are available through NCBI Sequence Read Archive (PRJNA513032). The source data underlying Figs. 1d, 2b–d, 3b, c, 4a–c and Supplementary Figs. 2c, d, 3–11 are provided as a Source Data file. All the other data supporting the findings of this study within the article and its supplementary information files are available from the corresponding author upon reasonable request.
