# Supplementary material for: Engineer chimeric Cas9 to expand PAM recognition based on evolutionary information
Source: Nat Commun. 2019 Feb 4;10:560. doi: 10.1038/s41467-019-08395-8 (PMC6361995; doi:10.1038/s41467-019-08395-8)
Supplement: Supplementary file 1 — Supplementary Information [file 41467_2019_8395_MOESM1_ESM.pdf]

Engineer chimeric Cas9 to expand PAM recognition based on evolutionary information

Ma et al.

Supplementary Information

Supplementary Figures

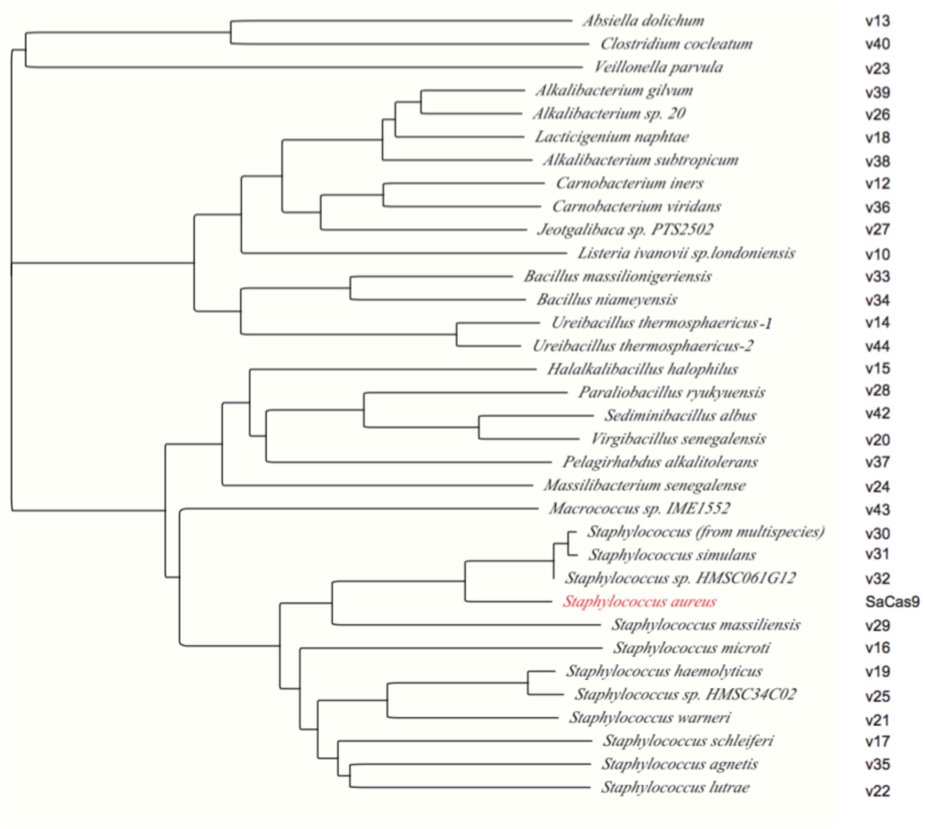

Supplementary Figure 1 | Phylogenetic tree of Cas9 orthologs.

The species names and numbers of the corresponding chimeric variants are listed. The phylogenetic tree was constructed by using Software Geneious R8.

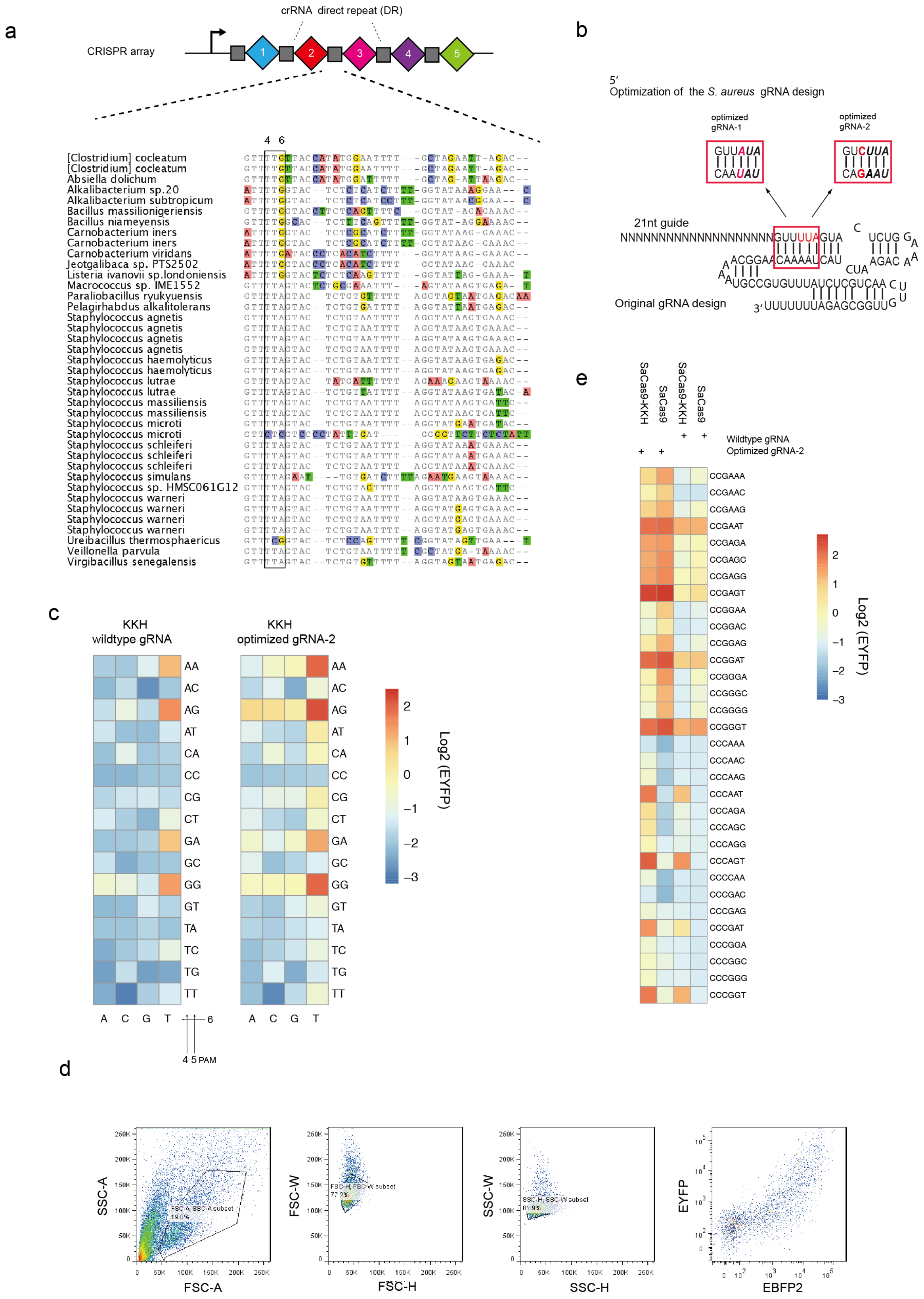

a
crRNA direct repeat (DR)
CRISPR array
1
2
3
4
5
4 6
[Clostridium] cocleatum
[Clostridium] cocleatum
Absiella dolichum
Alkalibacterium sp.20
Alkalibacterium subtropicum
Bacillus massilionigeriensis
Bacillus niameyensis
Carnobacterium iners
Carnobacterium iners
Carnobacterium viridans
Jeotgalibaca sp. PTS2502
Listeria ivanovii sp.londoniensis
Macrococcus sp. IME1552
Paraliobacillus ryukyuensis
Pelagirhabdus alkalitolerans
Staphylococcus agnetis
Staphylococcus agnetis
Staphylococcus agnetis
Staphylococcus agnetis
Staphylococcus haemolyticus
Staphylococcus haemolyticus
Staphylococcus lutrae
Staphylococcus lutrae
Staphylococcus massiliensis
Staphylococcus massiliensis
Staphylococcus microti
Staphylococcus microti
Staphylococcus schleiferi
Staphylococcus schleiferi
Staphylococcus schleiferi
Staphylococcus simulans
Staphylococcus sp. HMSC061G12
Staphylococcus warneri
Staphylococcus warneri
Staphylococcus warneri
Staphylococcus warneri
Ureibacillus thermosphaericus
Veillonella parvula
Virgibacillus senegalensis
b
5′
Optimization of the S. aureus gRNA design
optimized gRNA-1
optimized gRNA-2
GUUAUA
CAAUAU
GUCUUA
CAGAAU
21nt guide
NNNNNNNNNNNNNNNNNNNNNGUUUUAGUA
Original gRNA design
3′ UUUUUUUAGAGCGGUUG
c
KKH wildtype gRNA
KKH optimized gRNA-2
AA
AC
AG
AT
CA
CC
CG
CT
GA
GC
GG
GT
TA
TC
TG
TT
A C G T
4 5 PAM
6
Log2 (EYFP)
d
SSC-A
FSC-A
FSC-A, SSC-A subset
19.0%
FSC-W
FSC-H
FSC-H, FSC-W subset
77.2%
SSC-W
SSC-H
SSC-H, SSC-W subset
81.9%
EYFP
EBFP2
e
SaCas9-KKH
SaCas9
SaCas9-KKH
SaCas9
Wildtype gRNA
Optimized gRNA-2
CCGAAA
CCGAAC
CCGAAG
CCGAAT
CCGAGA
CCGAGC
CCGAGG
CCGAGT
CCGGAA
CCGGAC
CCGGAG
CCGGAT
CCGGGA
CCGGGC
CCGGGG
CCGGGT
CCCAAA
CCCAAC
CCCAAG
CCCAAT
CCCAGA
CCCAGC
CCCAGG
CCCAGT
CCCGAA
CCCGAC
CCCGAG
CCCGAT
CCCGGA
CCCGGC
CCCGGG
CCCGGT
Log2 (EYFP)

Supplementary Figure 2 | Optimization of gRNA scaffold for SaCas9 and its variants.

a. Sequence alignment of crRNA direct repeats in the corresponding species genome. **b.** The wildtype and two optimized gRNA scaffolds for SaCas9 variants. The optimized gRNA-1 was developed previously [1 2]. **c.** Cleavage activities of SaCas9-KKH (KKH) at CCCNNN PAMs with the wildtype gRNA scaffold or the optimized gRNA-2 scaffold were evaluated. **d.** Illustration of gates strategy of EYFP reconstitution assay. **e.** Comparison of SaCas9 and SaCas9-KKH with the wildtype gRNA or optimized gRNA-2 at sites with CCCRRN and CCGRRN PAMs. **c.** and **e.** Experiments were performed by using the EYFP reconstitution assay 3 days after transfection into HEK293FT cells. Data indicated the mean of three independent biological replicates. Source data are provided as a Source Data file.

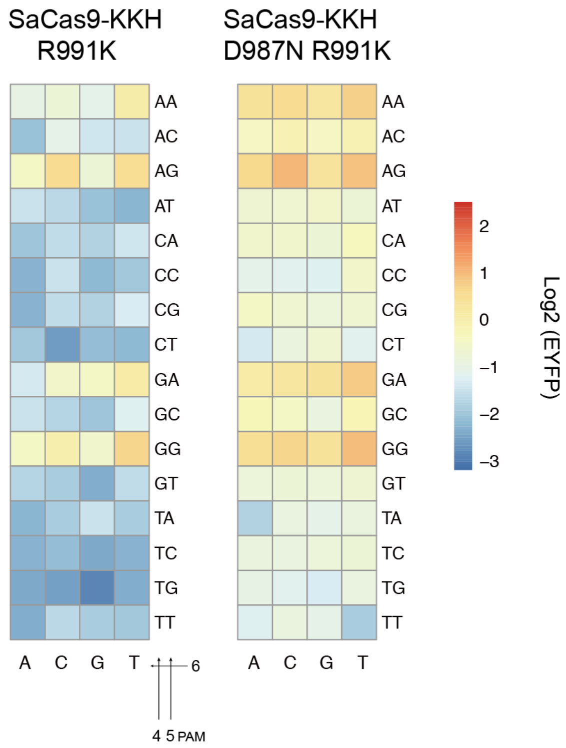

Supplementary Figure 3 | PAM preference of SaCas9-KKH based variants. Cleavage activities of SaCas9-KKH with the R991K mutation and SaCas9-KKH with D987N and R991K double mutations at CCCRRN PAMs by using the EYFP reconstitution assay (n=1). Source data are provided as a Source Data file.

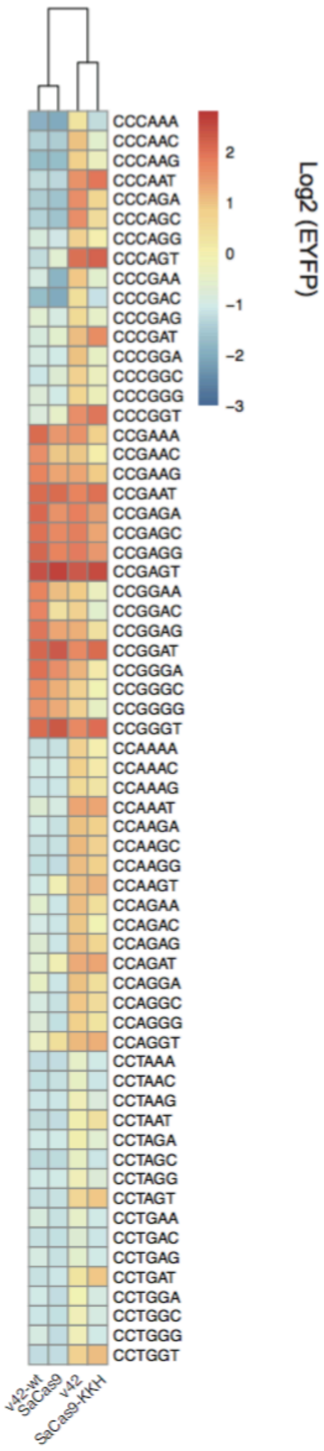

Supplementary Figure 4 | Comparison of variants at CCNRRN PAMs.
Cleavage activities of by SaCas9-KKH, SaCas9, cCas9 v42 and v42-wildtype (v42-wt) at CCNRRN PAMs using the EYFP reconstitution assay. Data represents the mean (n = 3 independent biologic replicates). Source data are provided as a Source Data file.

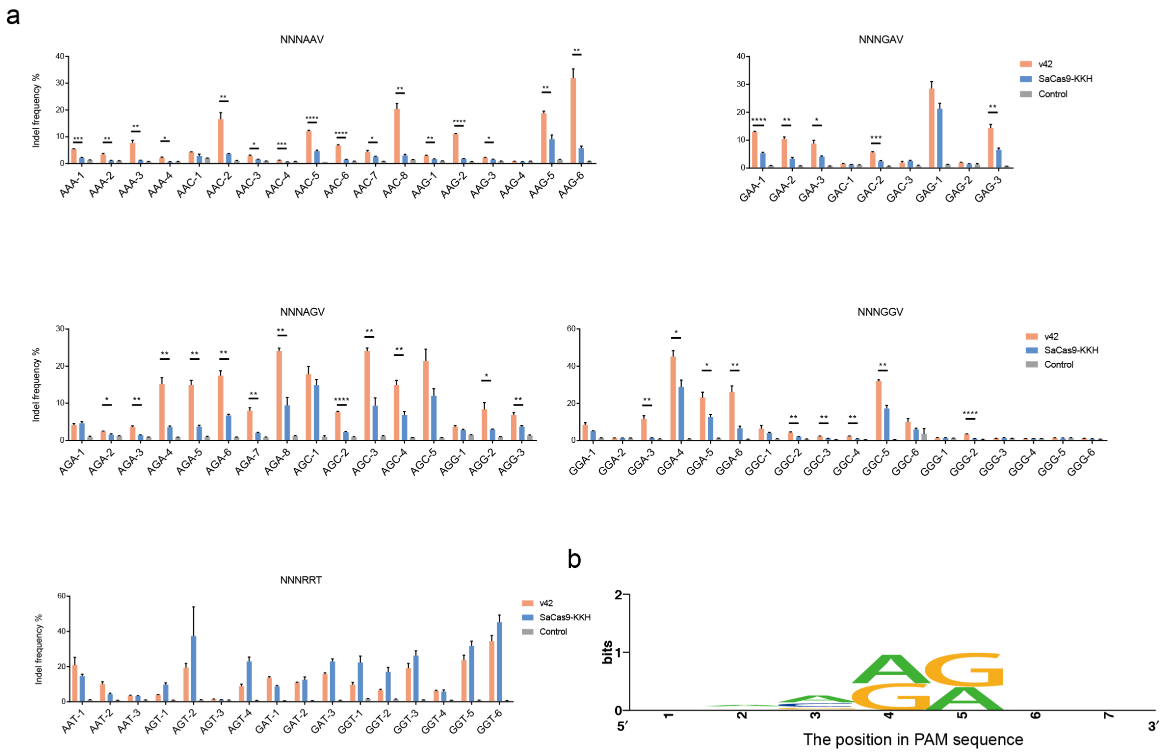

Supplementary Figure 5 | Indel frequency at NNNRRN PAMs.

a. Indel frequencies at sites generated by each gRNA measured by using the next generation sequencing. The information of gRNA sequences and primers used in this figure were listed in Supplementary Table 1. Error bar, s.e.m.; * p<0.05 (paired t-test, two-tailed); ** p<0.01 (paired t-test, two-tailed); *** p<0.001 (paired t-test, two-tailed); **** p<0.0001 (paired t-test, two-tailed). (n = 3 independent biologic replicates). **b.** Sequence logo shows the sequence preference in different PAM positions. The endogenous target sites with more than 5% mutation frequency generated by cCas9 v42 shown in Fig. 2d were used in the sequence logo analysis. Sequence logo was generated by using Web logo[3]. Source data are provided as a Source Data file.

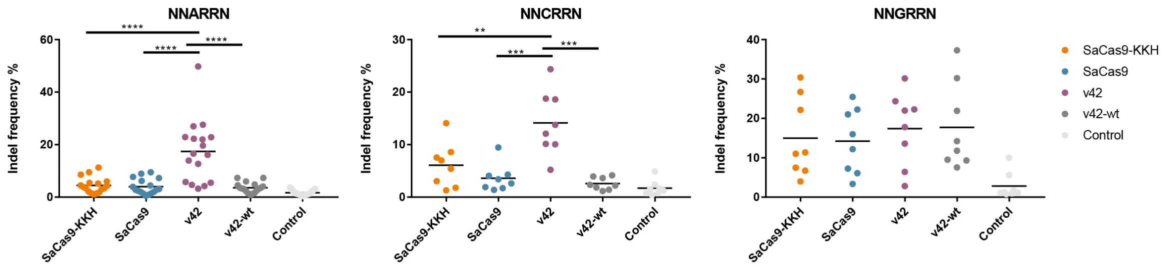

Supplementary Figure 6 | Indel frequency at NNVRRN PAMs.

SaCas9, SaCas9-KKH, cCas9 v42 and v42-wildtype (v42-wt) were transfected along with different gRNAs targeting endogenous sites with indicated NNVRRN PAMs (V=A, C or G). Indel frequencies were measured by using next generation sequencing as described in Methods. Each point represents the mean of one endogenous site. Black line indicates the mean indel frequency of all of the targets. The mean indel frequency of each endogenous site is listed in Supplementary Table 2. ** P<0.01 (paired t-test, two-tailed); *** P<0.001 (paired t-test, two-tailed); **** P<0.0001 (paired t-test, two-tailed). Source data are provided as a Source Data file.

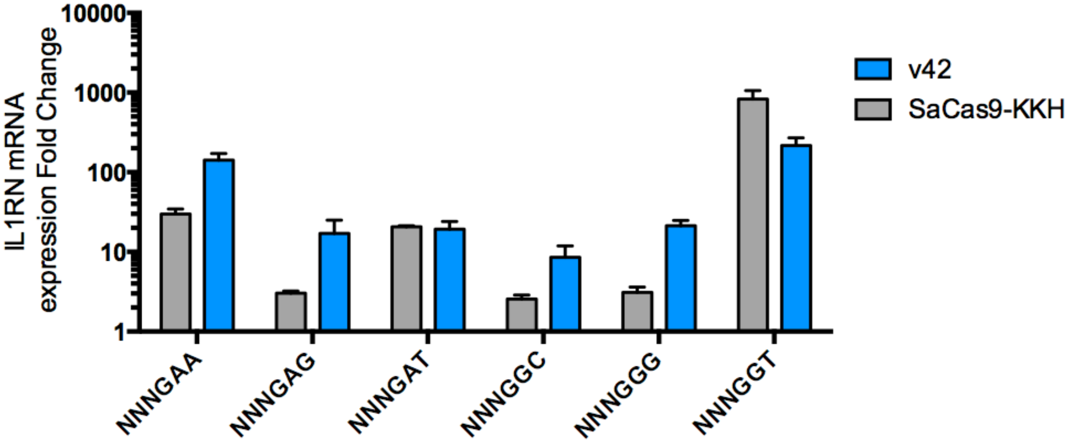

Supplementary Figure 7 | Transactivation efficiency of SaCas9 variants.

The *IL1RN* expression level was assayed using RT-PCR 4 days after transfecting dSaCas9-KKH:VPR or deactivated cCas9-v42:VPR in HEK293FT cells along with the corresponding gRNAs targeting at varying sites with indicated NNNRRN PAMs in the *IL1RN* promoter region. Data indicated the mean ± s.e.m. (n=2 or 3 independent replicates). Source data are provided as a Source Data file.

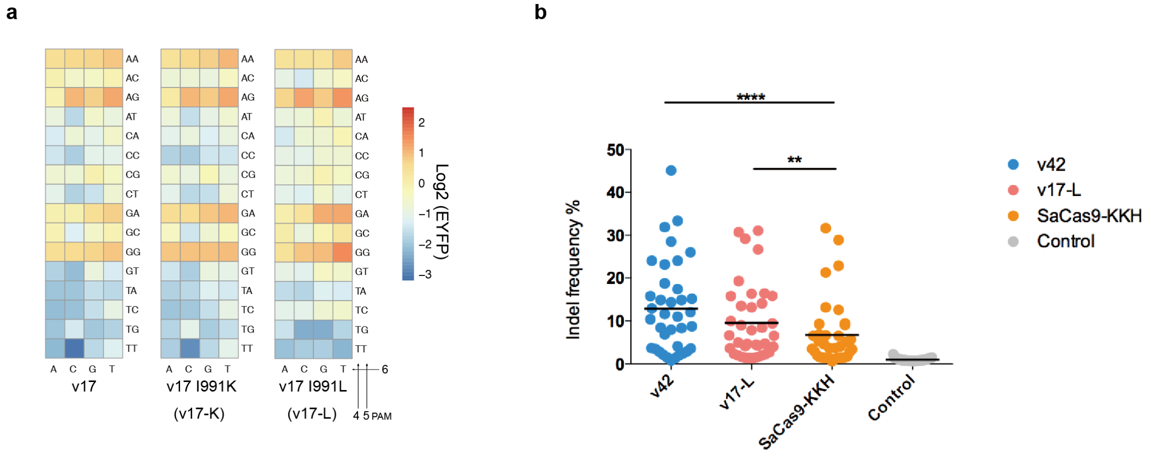

Supplementary Figure 8 | PAM preference of cCas9 v17 variants.

a. Cleavage activities of cCas9 v17, v17 with the I991K mutation (v17-K) and v17 with the I991L mutation (v17-L) at CCCRRN PAMs by using the EYFP reconstitution assay as shown in Fig. 1c (n=1). **b.** Indel frequencies generated by SaCas9-KKH, cCas9 v17-L and v42 at 37 different endogenous target sites with NNNRRV PAMs. Each point represents the mean of three independent biologic replicates targeting at one endogenous site. The black line indicates the mean value of all targets. The mean indel frequency of each endogenous site is listed in Supplementary Table 3. **** P<0.0001 (paired t-test, two-tailed); ** P<0.01 (paired t-test, two-tailed). Source data are provided as a Source Data file.

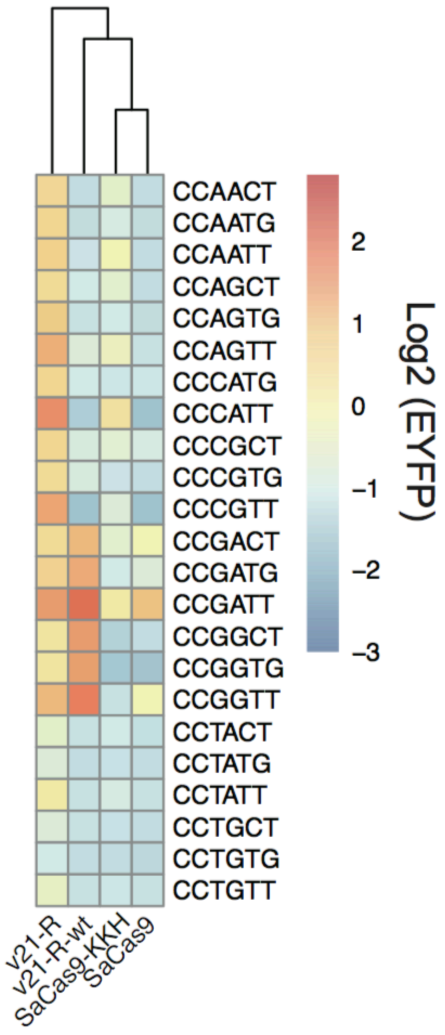

Supplementary Figure 9 | The preference in the third position of non-NNNRRN PAMs.

SaCas9, SaCas9-KKH, cCas9 v21 with the I991R mutation (v21-R), and v21 with the I991R mutation and the wildtype SaCas9 scaffold instead of SaCas9-KKH scaffold (v21-R-wt) were used to target the fluorescent reporter gene containing sites with indicated PAMs. The scheme of the fluorescent reporter assay is shown in Fig. 1c. Cleavage activities were measured by using FACS 3 days after transfection into HEK293FT cells. Data indicated the mean ± s.e.m. (n = 3 independent biologic replicates). Source data are provided as a Source Data file.

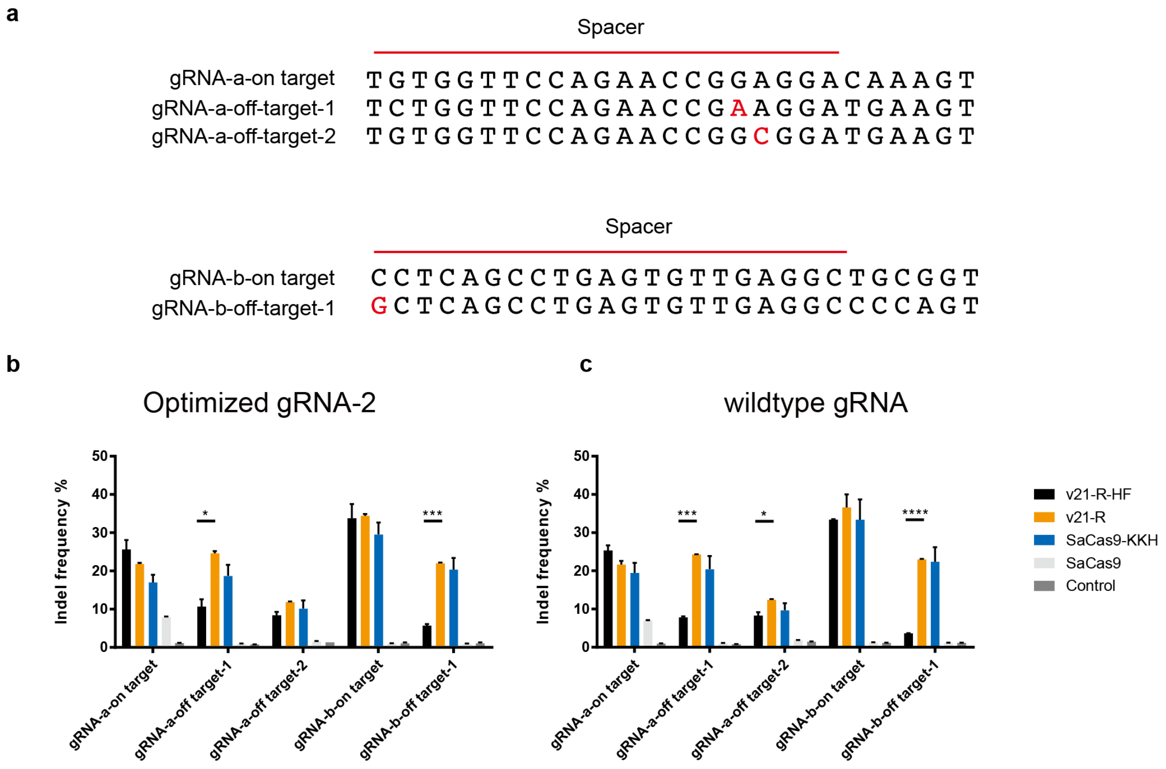

Supplementary Figure 10 | Off-target effect of cCas9 variants at endogenous target sites.

a. Sequence alignment of on-target sites and off-target sites of gRNA-a and gRNA-b. Letter in red indicates the mismatch. **b.** and **c.** Indel frequencies induced by SaCas9 and SaCas9-KKH, cCas9 v21-R and v21-R-HF were measured by using the next generation sequencing 5 days after transfection into HEK293FT cells. Data indicate the mean ± s.e.m. (n =2 independent replicates). **** P<0.0001 (paired t-test, two-tailed); *** P<0.001 (paired t-test, two-tailed); * P<0.05 (paired t-test, two-tailed). Source data are provided as a Source Data file.

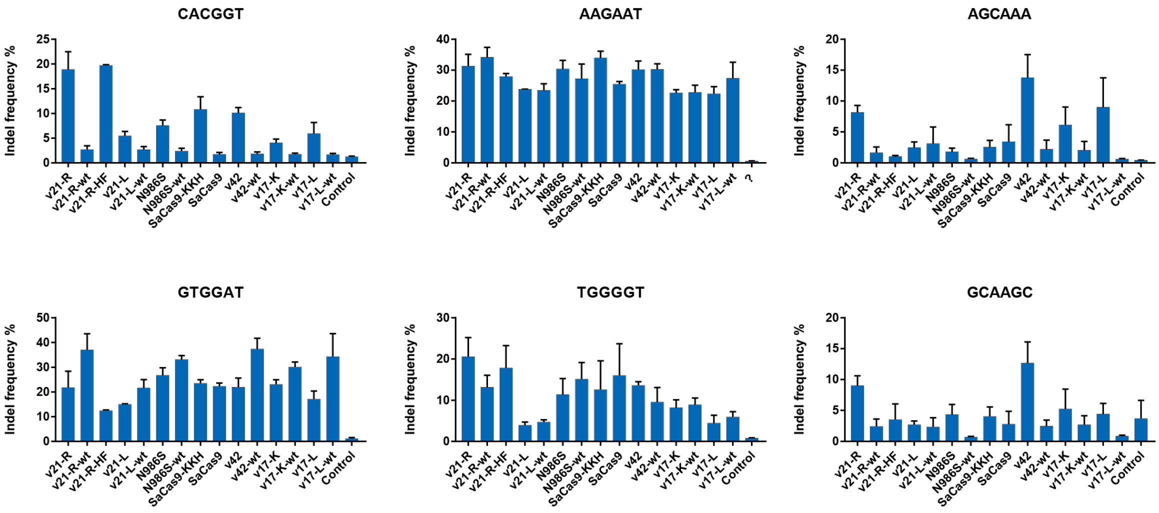

Supplementary Figure 11 | Genome editing efficiency of cCas9 variants at NNVRRN PAMs.

Indel frequencies induced by indicated SaCas9 variants at indicated NNVRRN PAMs were measured by the next generation sequencing 5 days after transfection into HEK293FT cells. Data indicated the mean ± s.e.m. (n =2 or 3 independent replicates). Source data are provided as a Source Data file.

Supplementary Tables

Supplementary Table 1| Indel frequency in Figure 2d and Supplementary Figure 5a

	v42	SaCas9-KKH	control
AAA-1	5.383788024	2.042248374	1.306799072
AAA-2	3.402756079	1.103170088	1.027052134
AAC-1	4.271990248	2.792671465	2.0112527
AAC-2	16.5550538	3.593279363	1.063740334
AAC-3	2.858080793	1.50079683	0.95394032
AAG-1	2.888478533	1.610489024	0.991765865
AAG-2	11.04153992	1.737134286	0.721921162
AAG-3	2.164166165	1.407361008	0.831974428
AAA-3	7.646681348	1.160600529	0.726599157
AAA-4	2.08220826	0.622104403	0.744622592
AAC-4	1.253566953	0.610897471	0.710144354
AAC-5	12.07589716	4.613157822	0.599518509
AAC-6	6.689099919	1.476474698	0.752239181
AAC-7	4.407234206	2.501331312	0.833189306
AAG-4	0.821875965	0.651561184	0.864294274
AAC-8	20.38138467	3.013375756	1.418486471
AAG-5	18.78359357	9.09596145	1.47857725
AAG-6	31.89745773	5.746877037	0.797583875
AAT-1	20.931038	14.64144917	1.163325043
AAT-2	10.04609089	4.283145261	0.852164045

AAT-3	3.399007223	3.254339049	0.936628732
AGA-1	4.11374284	4.589615638	0.853137062
AGA-2	2.423622466	1.503846807	1.165914836
AGC-1	17.82130348	14.82628023	0.963633002
AGC-2	7.626287989	2.211829938	0.862938518
AGG-1	3.683916248	2.799976128	1.378198967
AGG-2	8.347843498	2.903644571	0.923207805
AGT-1	3.981853808	9.769893369	0.863823287
AGT-2	19.36347817	37.49031066	1.013554984
AGA-3	3.602851554	1.304889034	0.780621408
AGA-4	15.19148426	3.535942727	0.761950958
AGA-5	14.91016734	3.688871135	0.854394237
AGA-6	17.43110729	6.689995764	0.838420507
AGA-7	7.975098005	2.022329368	0.763789016
AGT-3	1.453164233	1.160365415	0.775428699
AGT-4	8.912037516	22.94868291	0.720953644
AGA-8	24.08273229	9.442338254	1.115207821
AGG-3	6.888752331	3.703195595	1.288876399
AGC-3	24.08228367	9.33199372	1.078183362
AGC-4	14.90734792	6.895976845	0.794934783
AGC-5	21.35668053	12.01111414	0.675935126
GAA-1	12.93239191	5.288538631	0.877617219
GAA-2	10.38491926	3.390964915	0.766748453
GAG-1	28.52423813	21.29483955	1.277041971
GAG-2	1.963827355	1.381378736	1.362724879
GAA-3	8.752800468	4.046433489	0.76779198

GAG-3	14.34419176	6.571537047	0.654213655
GAT-2	11.02295959	12.59662582	0.644545319
GAT-3	15.77323106	22.9031055	0.819612298
GGA-1	8.451971395	5.084459189	1.318072034
GGC-1	6.378535503	4.026995258	0.897903612
GGT-1	9.73412833	22.26454243	1.808341393
GGT-2	6.580516174	16.97028217	1.406861519
GGT-3	19.07269305	26.2626449	0.987593676
GGA-2	1.332907671	1.350199474	1.306612487
GGC-2	4.302749423	2.102587518	0.807619368
GGG-1	1.463655043	1.391939008	1.252187458
GGT-4	6.074172152	5.759364608	0.801227704
GGA-3	11.63898719	1.450814542	0.876868468
GGA-4	45.10998651	28.89823637	0.859321826
GGC-3	2.282198285	1.159030711	0.598950081
GGC-4	2.28305406	0.940329907	0.614186307
GGC-5	32.07802121	17.22334605	0.622279078
GGG-2	3.460026689	1.082039826	0.631354085
GGA-5	23.17855202	12.62233624	1.122246355
GGA-6	26.00796462	6.545421396	0.75392965
GGC-6	10.01478885	5.987295983	3.618514981
GGT-5	23.64573915	31.71033343	0.868477208
GGT-6	34.4610265	45.2568938	0.742474882
GGG-3	1.228524418	1.371821451	1.245512197
GGG-4	1.119018655	1.125621074	1.120380982
GGG-5	1.29327884	1.109342438	1.407867728

GGG-6	1.234109603	0.916988899	0.730228194
GAC-1	1.50436264	1.276958779	1.080334222
GAC-2	5.584904789	2.488250127	0.647524559
GAC-3	1.966552459	2.524068701	0.82347796
GAT-1	13.6977706	8.746506016	0.522733219

Supplementary Table 2| Indel frequency in Supplementary Figure 6

gRNA sequence	SaCas9-KKH	SaCas9	v42	v42-wt	Control
GAGAACCACTTATAACTAACT	5.964977	6.152133	16.5705	7.345974	3.165357
GTTTTCGGGAAAACCAATGTG	6.72571	6.079152	2.826565	7.550714	0.949535
GGGATGGACCCTGTTATTCCC	2.192817	2.405336	13.91332	3.070477	2.865084
TCTGCATTCTGCCATCAGGGA	1.442926	1.423455	4.241936	1.23404	0.87536
TTATGAGCCCTTCTGCAAATG	3.306913	9.499751	26.96876	4.084066	1.567112
CTGGACCACTTGTCATTCACT	1.788048	1.981701	5.470734	3.560038	1.365287
GAAAGGACATTTGGAGGGTGC	7.468291	7.244384	6.404399	11.79585	1.062878
AGTGAATGACAAGTGGTCCAG	2.964778	2.032142	5.752632	3.160144	2.144033
GGAGCGGTCCCACTGCCACCC	3.037881	1.917354	12.09837	1.1653	1.629258
GCTTCCAGCCCCGCGAACAGC	3.979848	3.360766	17.7736	9.322766	1.725451
GAACTTGGATCCGAGCGGAGG	26.72098	22.26309	21.96992	37.29956	1.073405
TCTGTAGCTCACTGAAGGCTT	30.39664	25.44946	30.15816	30.21288	10.02323
TGGGCTTTATGGGCAAGCCAG	22.15709	21.03269	24.34252	21.91652	5.616783
TGCCGTGATCACAGGATAGCC	5.423019	4.289522	18.76475	3.641419	1.512041
GCATCATTTGGCTTGATCTTA	5.431097	4.404801	19.64185	3.593341	1.259107
ATTCTTAAGCCTTCAGTGAGC	1.296368	4.054265	5.215076	1.403914	0.764745

TCATTTGGCTTGATCTTATAA	4.470264	8.934931	22.84034	4.831125	1.495558
TTAAGAATTTGGGCTTTATGG	4.338785	2.775468	12.66736	2.467407	3.689073
GAATTTGGGCTTTATGGGCAA	8.584318	1.398834	18.62165	4.158306	4.898106
GCCGTGATCACAGGATAGCCT	9.493395	7.265513	22.87406	7.346522	2.652068
GGGGTTTTAAGGTAGTTCTCT	8.585745	1.309775	21.97974	1.614806	0.752924
GAAGCAGCTCCAGTGCCCAGA	7.525579	1.717651	10.06656	1.825731	1.242133
TCCGAGCGGAGGGTGGATGTT	11.33366	15.98731	13.57282	9.521295	0.779616
AGGCCCGCCGTCCTGGTCCAA	6.982134	2.601923	10.14279	2.325242	2.41301
TGGGTGAGTGGGAGAGTCCCG	11.02806	12.18742	22.26803	14.20093	1.233051
GGGCAGAGAGCTGGTTTTCGG	3.3531	0.54997	16.14013	0.955752	1.098554
GTTAAGAATTTGGGCTTTATG	14.08216	9.472067	24.35499	3.937848	0.742292
TGGCTTCCACGTAGTGCTCAA	1.066766	3.136055	4.675585	1.399205	0.703354
TAACTATGTGAAGTGTTACAC	5.184519	7.733171	49.75165	5.987027	2.387023
TGGAGGGTGCGTGGGGAGCTT	1.477927	0.921965	3.200313	2.291762	1.218692
CCTGATCTTCAAAGGTACAAA	3.857911	2.828971	22.22985	3.254008	0.441176
TTCAGGGCAAATAAGAGTAGA	11.28103	3.918283	27.56659	4.51224	1.068767
AGGTGTGTATTCCAGAATTGA	1.796273	3.411279	13.75203	2.204196	0.408876

Supplementary Table 3| Indel frequency in Supplementary Figure 8b

gRNA sequence	v42	SaCas9-KKH	v17-L	Control
GCGGAGGGTGGATGTTTGGGG	2.858081	1.500797	2.282631	0.95394
AACATCCACCCTCCGCTCGGA	2.888479	1.610489	6.546633	0.991766
GAGCAGACAACTCACAAATGC	11.04154	1.737134	15.83583	0.721921
GCGGGATGGACCCTGTTATTC	2.164166	1.407361	4.019905	0.831974

AATTATTTATAATTCAGGGCA	0.821876	0.651561	1.945215	0.864294
GGACCACAGGGAAGGCTGCCA	18.78359	9.095961	19.32154	1.478577
GTTAAGAATTTGGGCTTTATG	31.89746	5.746877	26.68633	0.797584
GGAGGCCGATGGTGGGTGAGT	4.113743	4.589616	2.030864	0.853137
GGGCTGGAAGCAGCTCCAGTG	2.423622	1.503847	1.62053	1.165915
ACGGTGCCGCGACCGGCTTGG	3.683916	2.799976	2.712772	1.378199
TGGCTTCCACGTAGTGCTCAA	8.347843	2.903645	3.630279	0.923208
ATTATTTATAATTCAGGGCAA	3.602852	1.304889	1.634604	0.780621
GCAACTATCATAGGTGTGTAT	15.19148	3.535943	6.630174	0.761951
CCTGATCTTCAAAGGTACAAA	14.91017	3.688871	4.316389	0.854394
TGCCGTGATCACAGGATAGCC	17.43111	6.689996	15.80242	0.838421
ATTCTTAAGCCTTCAGTGAGC	7.975098	2.022329	4.1961	0.763789
GACCACAGGGAAGGCTGCCAT	12.05179	13.19317	4.464219	2.267423
TTAAGAATTTGGGCTTTATGG	33.35647	31.65914	29.1707	0.787909
GCATCATTTGGCTTGATCTTA	24.08273	9.442338	16.4371	1.115208
TGCCATAAAAGCTGCCGTGAT	6.888752	3.703196	9.039727	1.288876
TCATTTGGCTTGATCTTATAA	24.08228	9.331994	16.36372	1.078183
TGCAGGCTATCCTGTGATCAC	14.90735	6.895977	4.646831	0.794935
CCAGGAGGGTGACTCAGGCTA	12.93239	5.288539	13.20753	0.877617
CATCATTTGGCTTGATCTTAT	10.38492	3.390965	9.431238	0.766748
GCCGTGATCACAGGATAGCCT	28.52424	21.29484	30.71284	1.277042
CAGTGGAATTCTTAAGCCTTC	1.963827	1.381379	1.364419	1.362725
CTGATCTTCAAAGGTACAAAG	8.7528	4.046433	7.839242	0.767792
TTATTTATAATTCAGGGCAAA	14.34419	6.571537	9.996689	0.654214
ATCTTCAAAGGTACAAAGTAA	15.79986	22.89803	13.50426	0.820129
GTCGCAGCTTCAGACCGCGGC	8.451971	5.084459	4.988769	1.318072

CACCCCTCCTCCTTTTTGCCG	1.332908	1.350199	1.348846	1.306612
ACACCCCTCCTCCTTTTTGCC	1.463655	1.391939	1.376424	1.252187
CTTACTTTGTACCTTTGAAGA	11.63899	1.450815	2.354955	0.876868
GGTACAAAGTAAGAAGAGAAA	45.10999	28.89824	31.06465	0.859322
ATTTGCCCTGAATTATAAATA	3.460027	1.08204	8.421338	0.631354
GCCATAAAAGCTGCCGTGATC	23.17855	12.62234	4.74802	1.122246
TTTGGGCTTTATGGGCAAGCC	26.00796	6.545421	14.08938	0.75393

Supplementary Table 4| Indel frequency in Figure 4b

gRNA sequence	v21-R	v21-L	v21-R-HF	SaCas9-KKH	N986S	v21-L-wt	N986S-wt	SaCas9	Control
AGACAGGAGGAGGAGGTCACC	11.11299	49.24419	22.86059	2.697692	13.07416	1.176115	1.126851	1.120731	1.032957
ATGTTAACCATGCTGCCAAAG	25.64272	14.94149	5.19863	9.475554	47.23149	1.114869	1.594376	1.323147	1.189963
AGTAGGGTGTGGCAGCTGAGA	16.87179	19.81117	9.857807	3.915293	14.42375	1.394264	1.120859	0.962022	1.175488
CCAAGGGGCATGGAAGGAAGC	26.34867	12.05672	24.61158	3.663447	18.85512	1.44885	1.604627	1.293039	1.438761
ATGATGGCTGCAGACATCCCG	35.7503	27.77496	33.75276	4.411669	17.91588	4.115427	2.954954	3.7021	1.240662
ACCTGGACCAAGGAGCTCAGC	16.22593	31.61956	19.61787	2.581841	9.984284	2.437051	1.559609	1.418458	1.461406

Supplementary Table 5| Indel frequency in Figure 4c

gRNA sequence	v21R	v21L	v21R-HF	SaCas9-KKH	N986S	v21L-wt	N986S-wt	SaCas9	control
CCTGCATGGTGGTTTCTAGGT	12.02654	5.262663	15.13692	3.241815	8.785131	9.025829	14.91207	5.125856	1.68582
AGGTCACCACCATGATCCTGG	48.21838	63.52833	61.56294	7.960178	46.43082	69.19564	45.32503	6.218725	1.172998
TCACCACCATGATCCTGGAGG	28.22389	6.927077	17.90429	5.482931	10.82454	40.5881	47.61172	21.90441	1.060181
TGCCTCCTCACTGCTTTCAGG	90.68085	88.7893	31.31988	31.41914	66.52682	85.56899	93.96794	71.88737	6.174891
AACTTGGATCCGAGCGGAGGG	17.86667	36.94025	54.45812	6.752216	26.25117	52.42698	52.8712	8.822457	1.234321

Supplementary Table 6| Information in Figure 2d and Supplementary Figure 5a

gRNA name	gRNA sequence	PAM position 1-7	Forward primer		Reverse primer	
AAA-1	GGGGGCAGAGAGCTGGTTTTC	gggaaaa	AAA-1F	TCTACACTCTTTCCCTACACGACGCTCTTCCGATCTTTCAGACCGCGGCCCAGGA	AAA-1R	GTGACTGGAGTTCAGACGTGTGCTCTTCCGATCTTCTCAGAGAACTTGGATCCGA
AAA-2	GGGTCCAACACATTGGTTTTC	ccgaaaa	AAA-2F	TCTACACTCTTTCCCTACACGACGCTCTTCCGATCTGCCGATGGTGGGTGAGTGGG	AAA-2R	GTGACTGGAGTTCAGACGTGTGCTCTTCCGATCTGTCCCGGGATGTCGTTTCAG

AAC-1	ACTGGAGCTGCTTCCAGCCCC	gcgaaca	AAC-1F	TCTACACTCTTTCCCTACACGACGCTCTTCCGATCTATCTCCCAGTGCCG	AAC-1R	GTGACTGGAGTTCAGACGTGTGCTCTTCCGATCTGCACTGCGCGGGA
AAC-2	GGGCAGAGAGCTGGTTTTCGG	gaaaacc	AAC-2F	TCTACACTCTTTCCCTACACGACGCTCTTCCGATCTCTTCAGACCGCGGCCCAGGA	AAC-2R	GTGACTGGAGTTCAGACGTGTGCTCTTCCGATCTTCTCAGAGAACTTGGATCCGA
AAC-3	GCGGAGGGTGGATGTTTGGGG	tccaaca	AAC-3F	TCTACACTCTTTCCCTACACGACGCTCTTCCGATCTGGAGAGTCCCGGAGAGCAGG	AAC-3R	GTGACTGGAGTTCAGACGTGTGCTCTTCCGATCTAGCCTAACTCTCCCATTCGT
AAG-1	AACATCCACCCTCCGCTCGGA	tccaagt	AAG-1F	TCTACACTCTTTCCCTACACGACGCTCTTCCGATCTGGAGAGTCCCGGAGAGCAGG	AAG-1R	GTGACTGGAGTTCAGACGTGTGCTCTTCCGATCTAGCTCAGCCTAACTCTCCCA
AAG-2	GAGCAGACAACTCACAAATGC	ttaaagc	AAG-2F	TCTACACTCTTTCCCTACACGACGCTCTTCCGATCTGGGGTCTCTTGCCA	AAG-2R	GTGACTGGAGTTCAGACGTGTGCTCTTCCGATCTTCCTCCTTTTTGCCGTTGGG
AAG-3	GCGGGATGGACCCTGTTATTC	cctaaga	AAG-3F	TCTACACTCTTTCCCTACACGACGCTCTTCCGATCTGAGAGTATCTGGCCTTGTGG	AAG-3R	GTGACTGGAGTTCAGACGTGTGCTCTTCCGATCTGGTGGAGGGAACAGCAAGGG
AAA-3	AGGTGTGTATTCCAGAATTGA	agcaaag	AAA-3F	TCTACACTCTTTCCCTACACGACGCTCTTCCGATCTGCCCTTCAGCACAAGTTACCA	AAA-3R	GTGACTGGAGTTCAGACGTGTGCTCTTCCGATCTATTGTTTATTGATGATTACA
AAA-4	AATTTAGCCTGTTTTCAAACT	tagaaat	AAA-4F	TCTACACTCTTTCCCTACACGACGCTCTTCCGATCTCATATCCATCTTTCTCTTCT	AAA-4R	GTGACTGGAGTTCAGACGTGTGCTCTTCCGATCTTCAAATAATATTTAAAATCA

AAC-4	CACCTATGATAGTTGCCTAAG	tgtaaca	AAC-4F	TCTACACTCTTTCCCTACACGACGCTCTTCCGATCTCAAGTTACCAGTTTTGACAA	AAC-4R	GTGACTGGAGTTCAGACGTGTGCTCTTCCGATCTTGATCTTGTATTGTTTATTG
AAC-5	TTCAGGGCAAATAAGAGTAGA	agaaacc	AAC-5F	TCTACACTCTTTCCCTACACGACGCTCTTCCGATCTTTGGCAGCTCTCCACTTCTG	AAC-5R	GTGACTGGAGTTCAGACGTGTGCTCTTCCGATCTTTCTATCTTTCCTGATCTTC
AAC-6	CTATGTGAAGTGTTACACTTA	ggcaact	AAC-6F	TCTACACTCTTTCCCTACACGACGCTCTTCCGATCTCAACACTCCCGGAATTTTGG	AAC-6R	GTGACTGGAGTTCAGACGTGTGCTCTTCCGATCTTAAATTCTAATCTTTCTTGA
AAC-7	AAAGATGGGAGCAGGGAAGTT	gggaacc	AAC-7F	TCTACACTCTTTCCCTACACGACGCTCTTCCGATCTTTCTGTTAAGTCAGGATTTC	AAC-7R	GTGACTGGAGTTCAGACGTGTGCTCTTCCGATCTAAGAGAAAGATGGATATGGT
AAG-4	AATTATTTATAATTCAGGGCA	aataaga	AAG-4F	TCTACACTCTTTCCCTACACGACGCTCTTCCGATCTTAGAGTCAGATTGGCAGCTC	AAG-4R	GTGACTGGAGTTCAGACGTGTGCTCTTCCGATCTTGATCTTCAAAGGTACAAAG
AAC-8	GTGATCACAGGATAGCCTGCA	gagaact	AAC-8F	TCTACACTCTTTCCCTACACGACGCTCTTCCGATCTGGTGACCCAGATGCCATGAG	AAC-8R	GTGACTGGAGTTCAGACGTGTGCTCTTCCGATCTTCCCCTCTAGATGACCTTCC
AAG-5	GGACCACAGGGAAGGCTGCCA	taaaagc	AAG-5F	TCTACACTCTTTCCCTACACGACGCTCTTCCGATCTGTTGATGCAGCTTGGGGAAT	AAG-5R	GTGACTGGAGTTCAGACGTGTGCTCTTCCGATCTTTCTAGGTGGGGTTTTAAGG
AAG-6	GTTAAGAATTTGGGCTTTATG	ggcaagc	AAG-6F	TCTACACTCTTTCCCTACACGACGCTCTTCCGATCTAAAGTGAGGTTCAGCAA	AAG-6R	GTGACTGGAGTTCAGACGTGTGCTCTTCCGATCTGGGCTTCCTACTGTC

AAT-1	TCTGTAGCTCACTGAAGGCTT	aagaatt	AAT-1F	TCTACACTCTTTCCCTACACGACGCTCTTCCGATCTAAAGAAGCTCTCATTAT	AAT-1R	GTGACTGGAGTTCAGACGTGTGCTCTTCCGATCTTCAGGTTGTAAAATGAAG
AAT-2	TGGGCTTTATGGGCAAGCCAG	tggaatt	AAT-2F	TCTACACTCTTTCCCTACACGACGCTCTTCCGATCTCCCATTTTACAGAAGAGG	AAT-2R	GTGACTGGAGTTCAGACGTGTGCTCTTCCGATCTCCTACTGTCCCCAAAAGC
AAT-3	AGAGAGCTGGTTTTCGGGAAA	accaatg	AAT-3F	TCTACACTCTTTCCCTACACGACGCTCTTCCGATCTAGACCGCGGCCCAGGA	AAT-3R	GTGACTGGAGTTCAGACGTGTGCTCTTCCGATCTTCGTTTCAGTTCTCAGA
AGA-1	GGAGGCCGATGGTGGGTGAGT	gggagag	AGA-1F	TCTACACTCTTTCCCTACACGACGCTCTTCCGATCTCGTGACATCCAGAAAACGCG	AGA-1R	ACTGGAGTTCAGACGTGTGCTCTTCCGATCTTGACCAAGGGTGGATGTTTGGGGTCC
AGA-2	GGGCTGGAAGCAGCTCCAGTG	cccagac	AGA-2F	TCTACACTCTTTCCCTACACGACGCTCTTCCGATCTGCCGTCCTGGTCCAAGCCGG	AGA-2R	ACTGGAGTTCAGACGTGTGCTCTTCCGATCTTAGCTTACGCTCACTCCCTTCACAAG
AGC-1	TGGGTGAGTGGGAGAGTCCCG	gagagca	AGC-1F	TCTACACTCTTTCCCTACACGACGCTCTTCCGATCTAAAACGCGAAACCTCAGGAA	AGC-1R	ACTGGAGTTCAGACGTGTGCTCTTCCGATCTACATGTAGGGTGGATGTTTGGGGTCC
AGC-2	AACACATTGGTTTTCCCGAAA	accagct	AGC-2F	TCTACACTCTTTCCCTACACGACGCTCTTCCGATCTCAGGAGGCCGATGGTGGGTG	AGC-2R	GTGACTGGAGTTCAGACGTGTGCTCTTCCGATCTGGATGTCGTTTCAGTTCTCA
AGG-1	ACGGTGCCGCGACCGGCTTGG	accagga	AGG-1F	TCTACACTCTTTCCCTACACGACGCTCTTCCGATCTCCCAGAGCCTCAGAGAAGGC	AGG-1R	GTGACTGGAGTTCAGACGTGTGCTCTTCCGATCTCGGTCCCACTGCCACCCTCC

AGG-2	TGGCTTCCACGTAGTGCTCAA	acaaggc	AGG-2F	TCTACACTCTTTCCCTACACGACGCTCTTCCGATCTCAGGTATAGGGCATGGGGGT	AGG-2R	GTGACTGGAGTTCAGACGTGTGCTCTTCCGATCTCAATGTCAAGGAAGGGGAAG
AGT-1	TTCCCTAAGACATGGCTTCCA	cgtagtg	AGT-1F	TCTACACTCTTTCCCTACACGACGCTCTTCCGATCTGCAGAGGCCAGGTATAGGG	AGT-1R	GTGACTGGAGTTCAGACGTGTGCTCTTCCGATCTCAAGGAAGGGGAAGAATATT
AGT-2	GCTGACTTGATGCCAAGCAGG	cccagtt	AGT-2F	TCTACACTCTTTCCCTACACGACGCTCTTCCGATCTTTACGCAGATAAGAACCAGT	AGT-2R	GTGACTGGAGTTCAGACGTGTGCTCTTCCGATCTGCCTCCCCTCCCACACACTC
AGA-3	ATTATTTATAATTCAGGGCAA	ataagag	AGA-3F	TCTACACTCTTTCCCTACACGACGCTCTTCCGATCTAGAGTCAGATTGGCAGCTCT	AGA-3R	GTGACTGGAGTTCAGACGTGTGCTCTTCCGATCTCTGATCTTCAAAGGTACAAA
AGA-4	GCAACTATCATAGGTGTGTAT	tccagaa	AGA-4F	TCTACACTCTTTCCCTACACGACGCTCTTCCGATCTCAGCACAAGTTACCAGTTTT	AGA-4R	GTGACTGGAGTTCAGACGTGTGCTCTTCCGATCTTTGTATTGTTTATTGATGAT
AGA-5	CCTGATCTTCAAAGGTACAAA	gtaagaa	AGA-5F	TCTACACTCTTTCCCTACACGACGCTCTTCCGATCTTTATTTATAATTCAGGGCAA	AGA-5R	GTGACTGGAGTTCAGACGTGTGCTCTTCCGATCTAAAAATATAGACAGAATGAT
AGA-6	TGCCGTGATCACAGGATAGCC	tgcagag	AGA-6F	TCTACACTCTTTCCCTACACGACGCTCTTCCGATCTAAGGTGACCCAGATGCCATG	AGA-6R	GTGACTGGAGTTCAGACGTGTGCTCTTCCGATCTCTCTAGATGACCTTCCCTGC
AGA-7	ATTCTTAAGCCTTCAGTGAGC	tacagag	AGA-7F	TCTACACTCTTTCCCTACACGACGCTCTTCCGATCTAGCTCTCATTATTATCCCCA	AGA-7R	GTGACTGGAGTTCAGACGTGTGCTCTTCCGATCTGGAGGTCAGGTTGTAAAATG

AGT-3	TATCCCCATTTTACAGAAGAG	gaaagtg	AGT-3F	TCTACACTCTTTCCCTACACGACGCTCTTCCGATCTCCTGACATTGTTTAAACTAT	AGT-3R	GTGACTGGAGTTCAGACGTGTGCTCTTCCGATCTTCACAAAATCTATGCTGGGC
AGT-4	GAATTTGGGCTTTATGGGCAA	gccagtg	AGT-4F	TCTACACTCTTTCCCTACACGACGCTCTTCCGATCTGAGGAAAGTGAGGTTCAGCA	AGT-4R	GTGACTGGAGTTCAGACGTGTGCTCTTCCGATCTTTCCTACTGTCCCCAAAAGC
AGA-8	GCATCATTTGGCTTGATCTTA	taaagaa	AGA-8F	TCTACACTCTTTCCCTACACGACGCTCTTCCGATCTAGGGATGAAAGCAGGGATGC	AGA-8R	ACTGGAGTTCAGACGTGTGCTCTTCCGATCTCAGATCGCCTTGCTGAACCTCACTTT
AGG-3	TGCCATAAAAGCTGCCGTGAT	cacagga	AGG-3F	TCTACACTCTTTCCCTACACGACGCTCTTCCGATCTTGCAGCTTGGGGAATGGTTC	AGG-3R	GTGACTGGAGTTCAGACGTGTGCTCTTCCGATCTTTCCCTGCATGGTGGTTTCT
AGC-3	TCATTTGGCTTGATCTTATAA	agaagct	AGC-3F	TCTACACTCTTTCCCTACACGACGCTCTTCCGATCTAGGGATGAAAGCAGGGATGC	AGC-3R	ACTGGAGTTCAGACGTGTGCTCTTCCGATCTACTTGAGCCTTGCTGAACCTCACTTT
AGC-4	TGCAGGCTATCCTGTGATCAC	ggcagct	AGC-4F	TCTACACTCTTTCCCTACACGACGCTCTTCCGATCTGGTGACCCAGATGCCATGAG	AGC-4R	GTGACTGGAGTTCAGACGTGTGCTCTTCCGATCTTCCCCTCTAGATGACCTTCC
AGC-5	TTAAGAATTTGGGCTTTATGG	gcaagcc	AGC-5F	TCTACACTCTTTCCCTACACGACGCTCTTCCGATCTGCCCTTCAGCACAAGTTACCA	AGC-5R	ACTGGAGTTCAGACGTGTGCTCTTCCGATCTCAGATCATTGTTTATTGATGATTACA
GAA-1	CCAGGAGGGTGACTCAGGCTA	gcagaaa	GAA-1F	TCTACACTCTTTCCCTACACGACGCTCTTCCGATCTAAGGCCCAGCTCAGTTCTCT	GAA-1R	GTGACTGGAGTTCAGACGTGTGCTCTTCCGATCTTGGCCAGCATGAGGAGATGG

GAA-2	CATCATTTGG CTTGATCTTAT	aaagaag	GAA-2F	TCTACACTCTTTCCCTACACGACGCTCTTCCGATCTAGGGATGAAAGCAGGGATGC	GAA-2R	GTGACTGGAGTTCAGACGTGTGCTCTTCCGATCTGCCTTGCTGAACCTCACTTT
GAG-1	GCCGTGATCA CAGGATAGCC T	gcagaga	GAG-1F	TCTACACTCTTTCCCTACACGACGCTCTTCCGATCTAGGTGACCCAGATGCCATGA	GAG-1R	GTGACTGGAGTTCAGACGTGTGCTCTTCCGATCTCCTCTAGATGACCTTCCCTG
GAG-2	CAGTGGAATT CTTAAGCCTTC	agtgagc	GAG-2F	TCTACACTCTTTCCCTACACGACGCTCTTCCGATCTATTATTATCCCCATTTTACA	GAG-2R	GTGACTGGAGTTCAGACGTGTGCTCTTCCGATCTGATGCAGGGAGGTCAGGTTG
GAA-3	CTGATCTTCA AAGGTACAAA G	taagaag	GAA-3F	TCTACACTCTTTCCCTACACGACGCTCTTCCGATCTATTATTTATAATTCAGGGCA	GAA-3R	GTGACTGGAGTTCAGACGTGTGCTCTTCCGATCTAAAATATAGACAGAATGATT
GAG-3	TTATTTATAAT TCAGGGCAAA	taagagt	GAG-3F	TCTACACTCTTTCCCTACACGACGCTCTTCCGATCTGAGTCAGATTGGCAGCTCTC	GAG-3R	GTGACTGGAGTTCAGACGTGTGCTCTTCCGATCTCCTGATCTTCAAAGGTACAA
GAT-2	TTCAATTCTGG AATACACACC	tatgata	GAT-2F	TCTACACTCTTTCCCTACACGACGCTCTTCCGATCTGCCCTTCAGCACAAGTTACCA	GAT-2R	GTGACTGGAGTTCAGACGTGTGCTCTTCCGATCTATTGTTTATTGATGATTACA
GAT-1	GAGAACCACT TATAACTAAC T	ccagata	GAT-1F	CTTTCCCTACACGACGCTCTTCCGATCTTTGCTGAGAGATAGGGCGGGGATTGC	GAT-1R	GAGTTCAGACGTGTGCTCTTCCGATCTGTGTCGCCTCCAAATGTCCTTTCCAT
GAT-3	CTCTTCTTACT TTGTACCTTT	gaagatc	GAT-3F	TCTACACTCTTTCCCTACACGACGCTCTTCCGATCTTCTTTCCCAATTATTTATAA	GAT-3R	TCTACACTCTTTCCCTACACGACGCTCTTCCGATCTTCTTTCCCAATTATTTATAA

GAC-1	AGACAAACTCAGCTCCTGCCA	ggagaca	GAC-1F	CTTTCCCTACACGACGCTCTTCCGATCTTCAGAGCCCTGTGTATAATTCTTCTA	GAC-1R	GAGTTCAGACGTGTGCTCTTCCGATCTCGAGTACGTGGTTTGCTAAGGACTCT
GAC-2	GCTTACAGTTTAAAGAATGGA	aaggaca	GAC-2F	CTTTCCCTACACGACGCTCTTCCGATCTTAGTAGTCCTAATGAAATGGAGAACC	GAC-2R	GAGTTCAGACGTGTGCTCTTCCGATCTGCCTAAAAAGGAGAGCTTTCAGTCTC
GAC-3	TGGAGGGTGCGTGGGGAGCTT	agagacc	GAC-3F	CTTTCCCTACACGACGCTCTTCCGATCTAGTAATACTCCAGATACAAATTGTGG	GAC-3R	CTTTCCCTACACGACGCTCTTCCGATCTAGTAATACTCCAGATACAAATTGTGG
GGA-1	GTCGCAGCTTCAGACCGCGGC	ccaggag	GGA-1F	TCTTTCCCTACACGACGCTCTTCCGATCTCGTCCCCTTTCCAGAGCTGT	GGA-1R	ACTGGAGTTCAGACGTGTGCTCTTCCGATCTTTCCCGAAAACCAGCTCTCT
GGC-1	GGACTCTCCCACTCACCCACC	atcggcc	GGC-1F	TCTTTCCCTACACGACGCTCTTCCGATCTAGAAAACGCGAAACCTCAGG	GGC-1R	ACTGGAGTTCAGACGTGTGCTCTTCCGATCTGCCAATAGGGTGGATGTTTGGGGTCC
GGT-1	AGGCCCGCCGTCCTGGTCCAA	gccggtc	GGT-1F	TCTTTCCCTACACGACGCTCTTCCGATCTCGCCGACACTTGAGCCCCCA	GGT-1R	ACTGGAGTTCAGACGTGTGCTCTTCCGATCTGATCAGTGCCACCCTCCAGCTGTTCG
GGT-2	GAAGCAGCTCCAGTGCCCAGA	cacggtg	GGT-2F	TCTTTCCCTACACGACGCTCTTCCGATCTCGATGTAAGGCGAGGGGGTGGATCTC	GGT-2R	ACTGGAGTTCAGACGTGTGCTCTTCCGATCTACTTGACTTCACAAGGGGCCAGGCGG
GGT-3	TCCGAGCGGAGGGTGGATGTT	tggggtc	GGT-3F	TCTTTCCCTACACGACGCTCTTCCGATCTCGATGTGGAGAGTCCCGGAGAGCAGG	GGT-3R	ACTGGAGTTCAGACGTGTGCTCTTCCGATCTTTAGGCGCCTAACTCTCCCATTCGTC

GGA-2	CACCCCTCCTC CTTTTTGCCG	ttgggag	GGA-2F	TCTTTCCCTACACGACGCTCTTCCGAT CTTTGTCTGCTCCCTGATGGCA	GGA-2R	ACTGGAGTTCAGACGTGTGCTCTTCCGA TCTCTGCCGTGACATTGTCCACA
GGC-2	CCATCCAAGT TGATGGGGAG C	agtggca	GGC-2F	TCTTTCCCTACACGACGCTCTTCCGAT CTTGGCTGCAGACCTGGGGACT	GGC-2R	ACTGGAGTTCAGACGTGTGCTCTTCCGA TCTTGTAGCTTCATGTCTCTGCA
GGG-1	ACACCCCTCC TCCTTTTTGCC	gttggga	GGG-1F	TCTTTCCCTACACGACGCTCTTCCGAT CTCATTTGTGAGTTGTCTGCTC	GGG-1R	ACTGGAGTTCAGACGTGTGCTCTTCCGA TCTCCACAAGGCCAGATACTCTC
GGT-4	ACCCCTGGGC AGGACGAAGT C	ccgggtc	GGT-4F	TCTTTCCCTACACGACGCTCTTCCGAT CTTGCCCACACACACACAACTG	GGT-4R	ACTGGAGTTCAGACGTGTGCTCTTCCGA TCTCTTCATGTCTCTGCATTCTG
GGA-3	CTTACTTTGTA CCTTTGAAGA	tcaggaa	GGA-3F	TCTTTCCCTACACGACGCTCTTCCGAT CTCTTGTATCTTTCCCAATTATTTATA A	GGA-3R	ACTGGAGTTCAGACGTGTGCTCTTCCGA TCTGAATGATTCTAATTTAGCCT
GGA-4	GGTACAAAGT AAGAAGAGAA A	gatggat	GGA-4F	TCTTTCCCTACACGACGCTCTTCCGAT CTCCATCTTTCCCAATTATTTA	GGA-4R	ACTGGAGTTCAGACGTGTGCTCTTCCGA TCTAATGATTCTAATTTAGCCTG
GGC-3	GTGTAACACT TCACATAGTT A	tatggct	GGC-3F	TCTTTCCCTACACGACGCTCTTCCGAT CTTGACCACACTCCCGGAATTTTGGC TT	GGC-3R	ACTGGAGTTCAGACGTGTGCTCTTCCGA TCTAATAAATTCTAATCTTTCTT
GGC-4	CTTTCCCAATT ATTTATAATT	cagggca	GGC-4F	TCTTTCCCTACACGACGCTCTTCCGAT CTACATGTCACTCCCGGAATTTTGGCT T	GGC-4R	ACTGGAGTTCAGACGTGTGCTCTTCCGA TCTCAAAGGTACAAAGTAAGAAG

GGC-5	TAACTATGTGAAGTGTTACAC	ttaggca	GGC-5F	TCTTTCCCTACACGACGCTCTTCCGATCTGAAACCATAGAGTCAGATTG	GGC-5R	ACTGGAGTTCAGACGTGTGCTCTTCCGATCTAAATAAATTCTAATCTTTCT
GGG-2	ATTTGCCCTGAATTATAAATA	attggga	GGG-2F	TCTTTCCCTACACGACGCTCTTCCGATCTGCCAATCACTCCCGGAATTTTGGCTT	GGG-2R	ACTGGAGTTCAGACGTGTGCTCTTCCGATCTGATCAGTCCTGATCTTCAAAGGTACA
GGA-5	GCCATAAAAGCTGCCGTGATC	acaggat	GGA-5F	TCTTTCCCTACACGACGCTCTTCCGATCTGCCAATTGCAGCTTGGGGAATGGTTC	GGA-5R	ACTGGAGTTCAGACGTGTGCTCTTCCGATCTTGGTTTCTAGGTGGGGTTTT
GGA-6	TTTGGGCTTTATGGGCAAGCC	agtggaa	GGA-6F	TCTTTCCCTACACGACGCTCTTCCGATCTGAAGAGGAAAGTGAGGTTCA	GGA-6R	ACTGGAGTTCAGACGTGTGCTCTTCCGATCTTACTGTCCCCAAAAGCCAAG
GGC-6	GAAGAGGAAAGTGAGGTTCAG	caaggct	GGC-6F	TCTTTCCCTACACGACGCTCTTCCGATCTACATGTCACACAGCATCATTTGGCTT	GGC-6R	ACTGGAGTTCAGACGTGTGCTCTTCCGATCTTGACCAATTTGGGCTTTATGGGCAAG
GGT-5	CCATTTTACAGAAGAGGAAAG	tgaggtt	GGT-5F	TCTTTCCCTACACGACGCTCTTCCGATCTTTAGGCGCCTTCATTCTCTGCATCAG	GGT-5R	ACTGGAGTTCAGACGTGTGCTCTTCCGATCTCGATGTGTGGAATTCTTAAGCCTTCA
GGT-6	CCCTGCATGGTGGTTTCTAGG	tggggtt	GGT-6F	TCTTTCCCTACACGACGCTCTTCCGATCTTTAGGCCTGCCATAAAAGCTGCCGTG	GGT-6R	ACTGGAGTTCAGACGTGTGCTCTTCCGATCTCGATGTAATGTTAGTGATCCCCAGTA
GGG-6	GATCCGAGCGGAGGGTGGATG	tttgggg	GGG-6F	CTTTCCCTACACGACGCTCTTCCGATCTCGAAGTGTAGCTCAGCCTAACTCTCC	GGG-6R	GAGTTCAGACGTGTGCTCTTCCGATCTCCTTCAGGAGAGTCCCGGAGAGCAGG

GGG-3	CAAGCCGGTC GCGGCACCGT G	tctgggc	GGG-3F	CTTTCCCTACACGACGCTCTTCCGATC TGCTATCCGGTCCCACTGCCACCCTCC	GGG-3R	GAGTTCAGACGTGTGCTCTTCCGATCTTT TTCGCCCCCAGAGCCTCAGAGAAG
GGG-4	ACCGCGGCCC AGGAGGCCGA T	ggtgggt	GGG-4F	CTTTCCCTACACGACGCTCTTCCGATC TTTTTGGAACACATTGGTTTTCCCGAA	GGG-4R	GAGTTCAGACGTGTGCTCTTCCGATCTT GTCACGAGCTGTCCCCCGTGACATC
GGG-5	CACTGCCACC CTCCAGCTGTT	cgcgggg	GGG-5F	CTTTCCCTACACGACGCTCTTCCGATC TACGGGGACGCTCACTCCCTTCACAA G	GGG-5R	GAGTTCAGACGTGTGCTCTTCCGATCTA GCGCGGTCCTGGTCCAAGCCGGTCG

Supplementary Table 7| Information in Supplementary Figure 6

gRNA sequence	PAM position 1-7	Forward primer		Reverse primer	
GAGAACCACTTA TAACTAACT	ccagata	SF6-1F	CTTTCCCTACACGACGCTCTTCCGATCTT TGCTGAGAGATAGGGCGGGGATTGC	SF6-1R	GAGTTCAGACGTGTGCTCTTCCGATCTGTGTC GCCTCCAAATGTCCTTTCCAT
GTTTTCGGGAAA ACCAATGTG	ttggacc	SF6-2F	CTTTCCCTACACGACGCTCTTCCGATCTC ACAAAATGTCGTTTCAGTTCTCAGA	SF6-2R	GAGTTCAGACGTGTGCTCTTCCGATCTGTGTG GCCCAGGAGGCCGATGGTGGG
TCTGCATTCTGCC ATCAGGGA	gcagaca	SF6-3F	CTTTCCCTACACGACGCTCTTCCGATCTT CGCAGGTTCTGAACACCCCTCCTCC	SF6-3R	GAGTTCAGACGTGTGCTCTTCCGATCTATTAGT CCCAGGGGTCTCTTGCCACT

GGGATGGACCCT GTTATTCCC	taagaca	SF6-4F	CTTTCCCTACACGACGCTCTTCCGATCTG ATATAGTGGAGGGAACAGCAAGGG	SF6-4R	GAGTTCAGACGTGTGCTCTTCCGATCTTGGCCT GAGAGTATCTGGCCTTGTGG
TTATGAGCCCTTC TGCAAATG	agagggt	SF6-5F	CTTTCCCTACACGACGCTCTTCCGATCTG GGGGCAGAGGAAGGAGCAGACCAGC	SF6-5R	GAGTTCAGACGTGTGCTCTTCCGATCTACCTC GTGCAGGGTTAGAGGAAATTG
CTGGACCACTTGT CATTCACT	tgagacc	SF6-6F	CTTTCCCTACACGACGCTCTTCCGATCTT CCGGAATAGATGGGTGTGGCGGGAG	SF6-6R	GAGTTCAGACGTGTGCTCTTCCGATCTGGGCC GCTGCTTTCTCTGCTGGGACA
GAAAGGACATTT GGAGGGTGC	gtgggga	SF6-7F	CTTTCCCTACACGACGCTCTTCCGATCTG TTACTCTTATAACTAACTCCAGATA	SF6-7R	GAGTTCAGACGTGTGCTCTTCCGATCTCGCTAT CCTGGCGCTGCTTGAAAGGA
AGTGAATGACAA GTGGTCCAG	ggagacc	SF6-8F	CTTTCCCTACACGACGCTCTTCCGATCTA AATGGATAGATGGGTGTGGCGGGAG	SF6-8R	GAGTTCAGACGTGTGCTCTTCCGATCTATACA CCTGCTTTCTCTGCTGGGACA
GGAGCGGTCCCA CTGCCACCC	tccagct	SF6-9F	CTTTCCCTACACGACGCTCTTCCGATCTA GTCTTTCCTGGTCCAAGCCGGTCGC	SF6-9R	GAGTTCAGACGTGTGCTCTTCCGATCTCGGCA GGGAAAGGGGACGCTCACTCC
GCTTCCAGCCCCG CGAACAGC	tggaggg	SF6-10F	CTTTCCCTACACGACGCTCTTCCGATCTG CATCAGGATCTCCCAGTGCCGAGGC	SF6-10R	GAGTTCAGACGTGTGCTCTTCCGATCTCGGCC CCTTCACAAGGGGCCAGGCGG
GAACTTGGATCC GAGCGGAGG	gtggatg	SF6-11F	CTTTCCCTACACGACGCTCTTCCGATCTT CACACCCGGTGTGTAGCTCAGCCTA	SF6-11R	GAGTTCAGACGTGTGCTCTTCCGATCTTGTCTT GGAGAGTCCCGGAGAGCAGG

TCTGTAGCTCACTGAAGGCTT	aagaatt	SF6-12F	CTTTCCCTACACGACGCTCTTCCGATCTCAGAAGCAGGTTGTAAAATGAAGTGG	SF6-12R	GAGTTCAGACGTGTGCTCTTCCGATCTATGATATAAAGAAGCTCTCATTATTA
TGGGCTTTATGGGCAAGCCAG	tggaatt	SF6-13F	CTTTCCCTACACGACGCTCTTCCGATCTCGACACACTGTCCCCAAAAGCCAAGA	SF6-13R	GAGTTCAGACGTGTGCTCTTCCGATCTTAAGTGCAGAAGAGGAAAGTGAGGTT
TGCCGTGATCACAGGATAGCC	tgcagag	SF6-14F	TCTACACTCTTTCCCTACACGACGCTCTTCCGATCTAAGGTGACCCAGATGCCATG	SF6-14R	GTGACTGGAGTTCAGACGTGTGCTCTTCCGATCTCTCTAGATGACCTTCCCTGC
GCATCATTTGGCTTGATCTTA	taaagaa	SF6-15F	TCTACACTCTTTCCCTACACGACGCTCTTCCGATCTCAGAGGGATGAAAGCAGGGATGC	SF6-15R	GTGACTGGAGTTCAGACGTGTGCTCTTCCGATCTCTGAGCCTTGCTGAACCTCACTTT
ATTCTTAAGCCTTCAGTGAGC	tacagag	SF6-16F	TCTACACTCTTTCCCTACACGACGCTCTTCCGATCTAGCTCTCATTATTATCCCCA	SF6-16R	GTGACTGGAGTTCAGACGTGTGCTCTTCCGATCTGGAGGTCAGGTTGTAAAATG
TCATTTGGCTTGATCTTATAA	agaagct	SF6-17F	TCTACACTCTTTCCCTACACGACGCTCTTCCGATCTAGGGATGAAAGCAGGGATGC	SF6-17R	GTGACTGGAGTTCAGACGTGTGCTCTTCCGATCTGCCTTGCTGAACCTCACTTT
TTAAGAATTTGGGCTTTATGG	gcaagcc	SF6-18F	TCTACACTCTTTCCCTACACGACGCTCTTCCGATCTAAAGTGAGGTTCAGCAA	SF6-18R	GTGACTGGAGTTCAGACGTGTGCTCTTCCGATCTGGGCTTCCTACTGTC
TGGCTTCCACGTAGTGCTCAA	acaaggc	SF6-19F	TACACTCTTTCCCTACACGACGCTCTTCCGATCTTTGCAGCAGGTATAGGGCATGGGG	SF6-19R	TGACTGGAGTTCAGACGTGTGCTCTTCCGATCTGGCTATCAATGTCAAGGAAGGGGAA

GAATTTGGGCTTT ATGGGCAA	gccagtg	SF6-20F	TCTACACTCTTTCCCTACACGACGCTCTT CCGATCTGAGGAAAGTGAGGTTCAGCA	SF6-20R	GTGACTGGAGTTCAGACGTGTGCTCTTCCGAT CTTTCCTACTGTCCCCAAAAGC
GCCGTGATCACA GGATAGCCT	gcagaga	SF6-21F	TCTACACTCTTTCCCTACACGACGCTCTT CCGATCTAGGTGACCCAGATGCCATGA	SF6-21R	GTGACTGGAGTTCAGACGTGTGCTCTTCCGAT CTCCTCTAGATGACCTTCCCTG
GGGGTTTTAAGG TAGTTCTCT	gcaggct	SF6-22F	TCTTTCCCTACACGACGCTCTTCCGATCT ACTTGATGAGGTTGTGGCCAGGACCA	SF6-22R	ACTGGAGTTCAGACGTGTGCTCTTCCGATCTT GACCAGCTTTCATCCCTGGCTACTG
GAAGCAGCTCCA GTGCCCAGA	cacggtg	SF6-23F	TCTTTCCCTACACGACGCTCTTCCGATCT CGATGTAAGGCGAGGGGGTGGATCTC	SF6-23R	ACTGGAGTTCAGACGTGTGCTCTTCCGATCTA CTTGACTTCACAAGGGGCCAGGCGG
TCCGAGCGGAGG GTGGATGTT	tggggtc	SF6-24F	TCTTTCCCTACACGACGCTCTTCCGATCT CGATGTGGAGAGTCCCGGAGAGCAGG	SF6-24R	ACTGGAGTTCAGACGTGTGCTCTTCCGATCTTT AGGCGCCTAACTCTCCCATTCGTC
AGGCCCGCCGTC CTGGTCCAA	gccggtc	SF6-25F	TCTTTCCCTACACGACGCTCTTCCGATCT CGCCGACACTTGAGCCCCCA	SF6-25R	ACTGGAGTTCAGACGTGTGCTCTTCCGATCTG ATCAGTGCCACCCTCCAGCTGTTCG
TGGGTGAGTGGG AGAGTCCCG	gagagca	SF6-26F	TCTACACTCTTTCCCTACACGACGCTCTT CCGATCTAAAACGCGAAACCTCAGGAA	SF6-26R	GTGACTGGAGTTCAGACGTGTGCTCTTCCGAT CTAGGGTGGATGTTTGGGGTCC
GGGCAGAGAGCT GGTTTTCGG	gaaaacc	SF6-27F	TCTACACTCTTTCCCTACACGACGCTCTT CCGATCTCTTCAGACCGCGGCCCAGGA	SF6-27R	GTGACTGGAGTTCAGACGTGTGCTCTTCCGAT CTTCTCAGAGAACTTGGATCCGA

TGGAGGGTGCGTGGGGAGCTT	agagacc	SF6-28F	CTTTCCCTACACGACGCTCTTCCGATCTAGTAATACTCCAGATACAAATTGTGG	SF6-28R	GAGTTCAGACGTGTGCTCTTCCGATCTGCCACTGGATATACACATGCCTGGCG
GTTAAGAATTTGGGCTTTATG	ggcaagc	SF6-29F	TCTACACTCTTTCCCTACACGACGCTCTTCCGATCTGGTCTAAAAGTGAGGTTCAGCAA	SF6-29R	GTGACTGGAGTTCAGACGTGTGCTCTTCCGATCTTAAGCTGGGCTTCCTACTGTC
TAACTATGTGAAGTGTTACAC	ttaggca	SF6-30F	TTTCCCTACACGACGCTCTTCCGATCTGTTCACGCCAATCACTCCCGGAATTTTGGC	SF6-30R	ACTGGAGTTCAGACGTGTGCTCTTCCGATCTACCTAGAAATAAATTCTAATCTTTCT
CCTGATCTTCAAAGGTACAAA	gtaagaa	SF6-31F	TCTTTCCCTACACGACGCTCTTCCGATCTTACCGTTTATTTATAATTCAGGGCAA	SF6-31R	TGACTGGAGTTCAGACGTGTGCTCTTCCGATCTCACTGAAAAAATATAGACAGAATGA
TTCAGGGCAAATAAGAGTAGA	agaaacc	SF6-32F	TACACTCTTTCCCTACACGACGCTCTTCCGATCTAGGCAATTGGCAGCTCTCCACTTC	SF6-32R	TGACTGGAGTTCAGACGTGTGCTCTTCCGATCTAATCGCTTCTATCTTTCCTGATCTT
AGGTGTGTATTCCAGAATTGA	agcaaag	SF6-33F	TACACTCTTTCCCTACACGACGCTCTTCCGATCTAGTCATGCCCTTCAGCACAAGTTAC	SF6-33R	GACTGGAGTTCAGACGTGTGCTCTTCCGATCTTCCGAGATTGTTTATTGATGATTA

Supplementary Table 8| Information in Supplementary Figure 8

gRNA sequence	PAM position 1-7	Forward primer		Reverse primer	
CCAGGAGGGTGA CTCAGGCTA	gcagaaa	SF8-1F	TCTACACTCTTTCCCTACACGACGCTCTTCC GATCTAAGGCCCAGCTCAGTTCTCT	SF8-1R	GTGACTGGAGTTCAGACGTGTGCTCTTCCG ATCTTGGCCAGCATGAGGAGATGG
CATCATTTGGCTT GATCTTAT	aaagaag	SF8-2F	TCTACACTCTTTCCCTACACGACGCTCTTCC GATCTAGGGATGAAAGCAGGGATGC	SF8-2R	GTGACTGGAGTTCAGACGTGTGCTCTTCCG ATCTGCCTTGCTGAACCTCACTTT
GCCGTGATCACA GGATAGCCT	gcagaga	SF8-3F	TCTACACTCTTTCCCTACACGACGCTCTTCC GATCTAGGTGACCCAGATGCCATGA	SF8-3R	GTGACTGGAGTTCAGACGTGTGCTCTTCCG ATCTCCTCTAGATGACCTTCCCTG
CAGTGGAATTCTT AAGCCTTC	agtgagc	SF8-4F	TCTACACTCTTTCCCTACACGACGCTCTTCC GATCTATTATTATCCCCATTTTACA	SF8-4R	GTGACTGGAGTTCAGACGTGTGCTCTTCCG ATCTGATGCAGGGAGGTCAGGTTG
CTGATCTTCAAAG GTACAAAG	taagaag	SF8-5F	TCTACACTCTTTCCCTACACGACGCTCTTCC GATCTATTATTTATAATTCAGGGCA	SF8-5R	GTGACTGGAGTTCAGACGTGTGCTCTTCCG ATCTAAAATATAGACAGAATGATT
TTATTTATAATTC AGGGCAAA	taagagt	SF8-6F	TCTACACTCTTTCCCTACACGACGCTCTTCC GATCTGAGTCAGATTGGCAGCTCTC	SF8-6R	GTGACTGGAGTTCAGACGTGTGCTCTTCCG ATCTCCTGATCTTCAAAGGTACAA
ATCTTCAAAGGT ACAAAGTAA	gaagaga	SF8-7F	TCTACACTCTTTCCCTACACGACGCTCTTCC GATCTTCTTTCCCAATTATTTATAA	SF8-7R	GTGACTGGAGTTCAGACGTGTGCTCTTCCG ATCTAAATATAGACAGAATGATTC

GTCGCAGCTTCA GACCGCGGC	ccaggag	SF8-8F	TCTTTCCCTACACGACGCTCTTCCGATCTCG TCCCCTTTCCAGAGCTGT	SF8-8R	ACTGGAGTTCAGACGTGTGCTCTTCCGATC TTTCCCGAAAACCAGCTCTCT
CACCCCTCCTCCT TTTTGCCG	ttgggag	SF8-9F	TCTTTCCCTACACGACGCTCTTCCGATCTTT GTCTGCTCCCTGATGGCA	SF8-9R	ACTGGAGTTCAGACGTGTGCTCTTCCGATC TCTGCCGTGACATTGTCCACA
ACACCCCTCCTCC TTTTTGCC	gttggga	SF8-10F	TCTTTCCCTACACGACGCTCTTCCGATCTCA TTTGTGAGTTGTCTGCTC	SF8-10R	ACTGGAGTTCAGACGTGTGCTCTTCCGATC TCCACAAGGCCAGATACTCTC
CTTACTTTGTACC TTTGAAGA	tcaggaa	SF8-11F	TCTTTCCCTACACGACGCTCTTCCGATCTCT TGTATCTTTCCCAATTATTTATAA	SF8-11R	ACTGGAGTTCAGACGTGTGCTCTTCCGATC TGAATGATTCTAATTTAGCCT
GGTACAAAGTAA GAAGAGAAA	gatggat	SF8-12F	TCTTTCCCTACACGACGCTCTTCCGATCTCC ATCTTTCCCAATTATTTA	SF8-12R	ACTGGAGTTCAGACGTGTGCTCTTCCGATC TAATGATTCTAATTTAGCCTG
ATTTGCCCTGAAT TATAAATA	attggga	SF8-13F	TCTTTCCCTACACGACGCTCTTCCGATCTGC CAATCACTCCCGGAATTTTGGCTT	SF8-13R	ACTGGAGTTCAGACGTGTGCTCTTCCGATC TGATCAGTCCTGATCTTCAAAGGTACA
GCCATAAAAGCT GCCGTGATC	acaggat	SF8-14F	TCTTTCCCTACACGACGCTCTTCCGATCTGC CAATTGCAGCTTGGGGAATGGTTC	SF8-14R	ACTGGAGTTCAGACGTGTGCTCTTCCGATC TTGGTTTCTAGGTGGGGTTTT
TTTGGGCTTTATG GGCAAGCC	agtggaa	SF8-15F	TCTTTCCCTACACGACGCTCTTCCGATCTGA AGAGGAAAGTGAGGTTCA	SF8-15R	ACTGGAGTTCAGACGTGTGCTCTTCCGATC TTACTGTCCCCAAAAGCCAAG

GGAGGCCGATGG TGGGTGAGT	gggagag	SF8-16F	TCTACACTCTTTCCCTACACGACGCTCTTCC GATCTCGTGACATCCAGAAAACGCG	SF8-16R	ACTGGAGTTCAGACGTGTGCTCTTCCGATC TTGACCAAGGGTGGATGTTTGGGGTCC
GGGCTGGAAGCA GCTCCAGTG	cccagac	SF8-17F	TCTACACTCTTTCCCTACACGACGCTCTTCC GATCTGCCGTCCTGGTCCAAGCCGG	SF8-17R	ACTGGAGTTCAGACGTGTGCTCTTCCGATC TTAGCTTACGCTCACTCCCTTCACAAG
ACGGTGCCGCGA CCGGCTTGG	accagga	SF8-18F	TCTACACTCTTTCCCTACACGACGCTCTTCC GATCTCCCAGAGCCTCAGAGAAGGC	SF8-18R	GTGACTGGAGTTCAGACGTGTGCTCTTCCG ATCTCGGTCCCACTGCCACCCTCC
TGGCTTCCACGTA GTGCTCAA	acaaggc	SF8-19F	TCTACACTCTTTCCCTACACGACGCTCTTCC GATCTCAGGTATAGGGCATGGGGGT	SF8-19R	GTGACTGGAGTTCAGACGTGTGCTCTTCCG ATCTCAATGTCAAGGAAGGGGAAG
ATTATTTATAATT CAGGGCAA	ataagag	SF8-20F	TCTACACTCTTTCCCTACACGACGCTCTTCC GATCTAGAGTCAGATTGGCAGCTCT	SF8-20R	GTGACTGGAGTTCAGACGTGTGCTCTTCCG ATCTCTGATCTTCAAAGGTACAAA
GCAACTATCATA GGTGTGTAT	tccagaa	SF8-21F	TCTACACTCTTTCCCTACACGACGCTCTTCC GATCTCAGCACAAGTTACCAGTTTT	SF8-21R	GTGACTGGAGTTCAGACGTGTGCTCTTCCG ATCTTTGTATTGTTTATTGATGAT
CCTGATCTTCAAA GGTACAAA	gtaagaa	SF8-22F	TCTACACTCTTTCCCTACACGACGCTCTTCC GATCTTTATTTATAATTCAGGGCAA	SF8-22R	GTGACTGGAGTTCAGACGTGTGCTCTTCCG ATCTAAAAATATAGACAGAATGAT
TGCCGTGATCAC AGGATAGCC	tgcagag	SF8-23F	TCTACACTCTTTCCCTACACGACGCTCTTCC GATCTAAGGTGACCCAGATGCCATG	SF8-23R	GTGACTGGAGTTCAGACGTGTGCTCTTCCG ATCTCTCTAGATGACCTTCCCTGC

ATTCTTAAGCCTT CAGTGAGC	tacagag	SF8-24F	TCTACACTCTTTCCCTACACGACGCTCTTCC GATCTAGCTCTCATTATTATCCCCA	SF8-24R	GTGACTGGAGTTCAGACGTGTGCTCTTCCG ATCTGGAGGTCAGGTTGTAAAATG
GACCACAGGGAA GGCTGCCAT	aaaagct	SF8-25F	TCTACACTCTTTCCCTACACGACGCTCTTCC GATCTTTGATGCAGCTTGGGGAATG	SF8-25R	GTGACTGGAGTTCAGACGTGTGCTCTTCCG ATCTTTTCTAGGTGGGGTTTTAAG
TTAAGAATTTGG GCTTTATGG	gcaagcc	SF8-26F	TCTACACTCTTTCCCTACACGACGCTCTTCC GATCTAAAGTGAGGTTCAGCAA	SF8-26R	GTGACTGGAGTTCAGACGTGTGCTCTTCCG ATCTGGGCTTCCTACTGTC
GCATCATTTGGCT TGATCTTA	taaagaa	SF8-27F	TCTACACTCTTTCCCTACACGACGCTCTTCC GATCTAGGGATGAAAGCAGGGATGC	SF8-27R	ACTGGAGTTCAGACGTGTGCTCTTCCGATC TCAGATCGCCTTGCTGAACCTCACTTT
TGCCATAAAAGC TGCCGTGAT	cacagga	SF8-28F	TCTACACTCTTTCCCTACACGACGCTCTTCC GATCTTGCAGCTTGGGGAATGGTTC	SF8-28R	GTGACTGGAGTTCAGACGTGTGCTCTTCCG ATCTTTCCCTGCATGGTGGTTTCT
TCATTTGGCTTGA TCTTATAA	agaagct	SF8-29F	TCTACACTCTTTCCCTACACGACGCTCTTCC GATCTAGGGATGAAAGCAGGGATGC	SF8-29R	ACTGGAGTTCAGACGTGTGCTCTTCCGATC TACTTGAGCCTTGCTGAACCTCACTTT
TGCAGGCTATCCT GTGATCAC	ggcagct	SF8-30F	TCTACACTCTTTCCCTACACGACGCTCTTCC GATCTGGTGACCCAGATGCCATGAG	SF8-30R	GTGACTGGAGTTCAGACGTGTGCTCTTCCG ATCTTCCCCTCTAGATGACCTTCC
GCGGAGGGTGGA TGTTTGGGG	tccaaca	SF8-31F	TCTACACTCTTTCCCTACACGACGCTCTTCC GATCTGGAGAGTCCCGGAGAGCAGG	SF8-31R	GTGACTGGAGTTCAGACGTGTGCTCTTCCG ATCTAGCCTAACTCTCCCATTCGT

AACATCCACCCTCCGCTCGGA	tccaagt	SF8-32F	TCTACACTCTTTCCCTACACGACGCTCTTCCGATCTGGAGAGTCCCGGAGAGCAGG	SF8-32R	GTGACTGGAGTTCAGACGTGTGCTCTTCCGATCTAGCTCAGCCTAACTCTCCCA
GAGCAGACAACTCACAAATGC	ttaaagc	SF8-33F	TCTACACTCTTTCCCTACACGACGCTCTTCCGATCTGGGGTCTCTTGCCA	SF8-33R	GTGACTGGAGTTCAGACGTGTGCTCTTCCGATCTTCCTCCTTTTTGCCGTTGGG
GCGGGATGGACCCTGTTATTC	cctaaga	SF8-34F	TCTACACTCTTTCCCTACACGACGCTCTTCCGATCTGAGAGTATCTGGCCTTGTGG	SF8-34R	GTGACTGGAGTTCAGACGTGTGCTCTTCCGATCTGGTGGAGGGAACAGCAAGGG
AATTATTTATAATTCAGGGCA	aataaga	SF8-35F	TCTACACTCTTTCCCTACACGACGCTCTTCCGATCTTAGAGTCAGATTGGCAGCTC	SF8-35R	GTGACTGGAGTTCAGACGTGTGCTCTTCCGATCTTGATCTTCAAAGGTACAAAG
GGACCACAGGGAAGGCTGCCA	taaaagc	SF8-36F	TCTACACTCTTTCCCTACACGACGCTCTTCCGATCTGTTGATGCAGCTTGGGGAAT	SF8-36R	GTGACTGGAGTTCAGACGTGTGCTCTTCCGATCTTTCTAGGTGGGGTTTTAAGG
GTTAAGAATTTGGGCTTTATG	ggcaagc	SF8-37F	TCTACACTCTTTCCCTACACGACGCTCTTCCGATCTAAAGTGAGGTTCAGCAA	SF8-37R	GTGACTGGAGTTCAGACGTGTGCTCTTCCGATCTGGGCTTCCTACTGTC

Supplementary Table 9| Information in Figure 4b and 4c

gRNA sequence	PAM position 1-7	Forward primer		Reverse primer	
AGACAGGAGGAG GAGGTCACC	accatga	F4bc-1F	TCTTTCCCTACACGACGCTCTTCCGATCT ATCACGGACATCCCGAGAGTGACCAC	F4bc-1R	ACTGGAGTTCAGACGTGTGCTCTTCCGATCT GCTCAGACTCAGGGCCATCC
TGCCTCCTCACTG CTTTCAGG	ccgatgc	F4bc-2F	TCTTTCCCTACACGACGCTCTTCCGATCT CAAGGAGCAGATGTTAACCA	F4bc-2R	ACTGGAGTTCAGACGTGTGCTCTTCCGATCT GAGGTTCTGCGTCTCACCAC
ATGTTAACCATGC TGCCAAAG	gaaattc	F4bc-3F	TCTTTCCCTACACGACGCTCTTCCGATCT ATCAGGGGAGGAGGTGACCAGGGGAC	F4bc-3R	ACTGGAGTTCAGACGTGTGCTCTTCCGATCT CAGATCCTGCCTCCTCACTGCTTTCAGG
AACTTGGATCCG AGCGGAGGG	tggatgt	F4bc-4F	TCTTTCCCTACACGACGCTCTTCCGATCT TTAGGCGGAGAGTCCCGGAGAGCAGG	F4bc-4R	ACTGGAGTTCAGACGTGTGCTCTTCCGATCT GACGAATGGGAGAGTTAGGC
CCTGCATGGTGGT TTCTAGGT	ggggttt	F4bc-5F	TCTTTCCCTACACGACGCTCTTCCGATCT GATCAGCTGCCATAAAAGCTGCCGTG	F4bc-5R	ACTGGAGTTCAGACGTGTGCTCTTCCGATCT ACTTGAAATGTTAGTGATCCCCAGTA
AGTAGGGTGTGG CAGCTGAGA	ggagctg	F4bc-6F	TCTTTCCCTACACGACGCTCTTCCGATCT CAGATCTCAGGTCAGAGAAGGCTTCC	F4bc-6R	ACTGGAGTTCAGACGTGTGCTCTTCCGATCT TTAGGCAGCTCTCCTGTCACCCAAAG
CCAAGGGGCATG GAAGGAAGC	ggagttt	F4bc-7F	TCTTTCCCTACACGACGCTCTTCCGATCT GAAGGGTGGGTGAGGGCTTG	F4bc-7R	ACTGGAGTTCAGACGTGTGCTCTTCCGATCT GCTTCCTGGCTGCTCTCCTG

AGGTCACCACCA TGATCCTGG	aggatga	F4bc-8F	TCTTTCCCTACACGACGCTCTTCCGATCT GACATCCCGAGAGTGACCAC	F4bc-8R	ACTGGAGTTCAGACGTGTGCTCTTCCGATCT AGGGCTCAGACTCAGGGCCA
TCACCACCATGAT CCTGGAGG	atgactc	F4bc-9F	TCTTTCCCTACACGACGCTCTTCCGATCT AGACATCCCGAGAGTGACCA	F4bc-9R	ACTGGAGTTCAGACGTGTGCTCTTCCGATCT GAAAGGGCTCAGACTCAGGG
ATGATGGCTGCA GACATCCCG	agagtga	F4bc-10F	TCTTTCCCTACACGACGCTCTTCCGATCT GTGTCTTACCTGAGAGCCTG	F4bc-10R	ACTGGAGTTCAGACGTGTGCTCTTCCGATCT CTCCTGTCTATCTTCCTCTT
ACCTGGACCAAG GAGCTCAGC	ggagtgg	F4bc-11F	TCTTTCCCTACACGACGCTCTTCCGATCT CTAAGTGATTGCCCCAGGAC	F4bc-11R	ACTGGAGTTCAGACGTGTGCTCTTCCGATCT GGCTAGTCTTGCACCCAGGAGTCATC

Supplementary Table 10| Information in Supplementary Figure 7

spacer sequence	PAM position 1-7	IL1RN forward primer	IL1RN reverse primer	β-ACTIN forward primer	β-ACTIN reverse primer	gene name
AGGCCCTTACC AGATCCCTGT	tgagaag	GGAATCCATGGAG GGAAGAT	TGTTCTCGCTCAGG TCAGTG	CATGTACGTTGCTA TCCAGGC	CTCCTTAATGTCAC GCACGAT	IL1RN
TGCCCTTGCTGT TCCCTCCAC	ctggaat	GGAATCCATGGAG GGAAGAT	TGTTCTCGCTCAGG TCAGTG	CATGTACGTTGCTA TCCAGGC	CTCCTTAATGTCAC GCACGAT	IL1RN
CTCCTCCTTTTT GCCGTTGGG	agcgaac	GGAATCCATGGAG GGAAGAT	TGTTCTCGCTCAGG TCAGTG	CATGTACGTTGCTA TCCAGGC	CTCCTTAATGTCAC GCACGAT	IL1RN

CCAGGAGGGTGACTCAGGCTA	gcagaaa	GGAATCCATGGAGGGAAGAT	TGTTCTCGCTCAGGTCAGTG	CATGTACGTTGCTATCCAGGC	CTCCTTAATGTCACGCACGAT	IL1RN
GAAGGCCCTTACCAGATCCCT	gttgaga	GGAATCCATGGAGGGAAGAT	TGTTCTCGCTCAGGTCAGTG	CATGTACGTTGCTATCCAGGC	CTCCTTAATGTCACGCACGAT	IL1RN
CTCCACTCTTATGTCACCCTC	tcagaga	GGAATCCATGGAGGGAAGAT	TGTTCTCGCTCAGGTCAGTG	CATGTACGTTGCTATCCAGGC	CTCCTTAATGTCACGCACGAT	IL1RN
ACCCCTCCTCCTTTTTGCCGT	tgggagc	GGAATCCATGGAGGGAAGAT	TGTTCTCGCTCAGGTCAGTG	CATGTACGTTGCTATCCAGGC	CTCCTTAATGTCACGCACGAT	IL1RN
ATGCCAAGCAGGCCCAGTTTC	caggagg	GGAATCCATGGAGGGAAGAT	TGTTCTCGCTCAGGTCAGTG	CATGTACGTTGCTATCCAGGC	CTCCTTAATGTCACGCACGAT	IL1RN
AGAGGAAATTGAAGGCCCTTA	ccagatc	GGAATCCATGGAGGGAAGAT	TGTTCTCGCTCAGGTCAGTG	CATGTACGTTGCTATCCAGGC	CTCCTTAATGTCACGCACGAT	IL1RN
CCCTCAAAAGCATATCCTGCT	ttggatt	GGAATCCATGGAGGGAAGAT	TGTTCTCGCTCAGGTCAGTG	CATGTACGTTGCTATCCAGGC	CTCCTTAATGTCACGCACGAT	IL1RN
CAAATGCTTAAAGCCATCCAA	gttgatg	GGAATCCATGGAGGGAAGAT	TGTTCTCGCTCAGGTCAGTG	CATGTACGTTGCTATCCAGGC	CTCCTTAATGTCACGCACGAT	IL1RN
ACAGAGGGTTGGCCAGCATGA	ggagatg	GGAATCCATGGAGGGAAGAT	TGTTCTCGCTCAGGTCAGTG	CATGTACGTTGCTATCCAGGC	CTCCTTAATGTCACGCACGAT	IL1RN
TGTCAGGAGGGACAGATTGTA	ggtggca	GGAATCCATGGAGGGAAGAT	TGTTCTCGCTCAGGTCAGTG	CATGTACGTTGCTATCCAGGC	CTCCTTAATGTCACGCACGAT	IL1RN
GGACCCTGTTATTCCCTAAGA	catggct	GGAATCCATGGAGGGAAGAT	TGTTCTCGCTCAGGTCAGTG	CATGTACGTTGCTATCCAGGC	CTCCTTAATGTCACGCACGAT	IL1RN

CCATCCAAGTTGATGGGGAGC	agtggca	GGAATCCATGGAGGGAAGAT	TGTTCTCGCTCAGGTCAGTG	CATGTACGTTGCTATCCAGGC	CTCCTTAATGTCACGCACGAT	IL1RN
AGGGTTGGCCAGCATGAGGAG	atgggcc	GGAATCCATGGAGGGAAGAT	TGTTCTCGCTCAGGTCAGTG	CATGTACGTTGCTATCCAGGC	CTCCTTAATGTCACGCACGAT	IL1RN
TTATGAGCCCTTCTGCAAATG	agagggt	GGAATCCATGGAGGGAAGAT	TGTTCTCGCTCAGGTCAGTG	CATGTACGTTGCTATCCAGGC	CTCCTTAATGTCACGCACGAT	IL1RN
ACTCTTATGTCACCCTCTCAG	agagggc	GGAATCCATGGAGGGAAGAT	TGTTCTCGCTCAGGTCAGTG	CATGTACGTTGCTATCCAGGC	CTCCTTAATGTCACGCACGAT	IL1RN
ACACCCCTCCTCCTTTTTGCC	gttggga	GGAATCCATGGAGGGAAGAT	TGTTCTCGCTCAGGTCAGTG	CATGTACGTTGCTATCCAGGC	CTCCTTAATGTCACGCACGAT	IL1RN
GAGGGTTGGCCAGCATGAGGA	gatgggc	GGAATCCATGGAGGGAAGAT	TGTTCTCGCTCAGGTCAGTG	CATGTACGTTGCTATCCAGGC	CTCCTTAATGTCACGCACGAT	IL1RN
TATGAGCCCTTCTGCAAATGA	gagggtt	GGAATCCATGGAGGGAAGAT	TGTTCTCGCTCAGGTCAGTG	CATGTACGTTGCTATCCAGGC	CTCCTTAATGTCACGCACGAT	IL1RN
CTTCCCCTTCCTTGACATTGC	tcaggtc	GGAATCCATGGAGGGAAGAT	TGTTCTCGCTCAGGTCAGTG	CATGTACGTTGCTATCCAGGC	CTCCTTAATGTCACGCACGAT	IL1RN
ACCCCTGGGCAGGACGAAGTC	ccgggtc	GGAATCCATGGAGGGAAGAT	TGTTCTCGCTCAGGTCAGTG	CATGTACGTTGCTATCCAGGC	CTCCTTAATGTCACGCACGAT	IL1RN
CCAAGCAGGCCCAGTTTCCAG	gagggtg	GGAATCCATGGAGGGAAGAT	TGTTCTCGCTCAGGTCAGTG	CATGTACGTTGCTATCCAGGC	CTCCTTAATGTCACGCACGAT	IL1RN

Supplementary Table 11| Information in Supplementary Figure 10

gRNA name	Forward primer		Reverse primer	
gRNA-a-off target-1	aoff-1F	TCTACACTCTTTCCCTACACGACGCTCTTCCGATCTTTATTTGACACGAAAACGTC	aoff-1R	GTGACTGGAGTTCAGACGTGTGCTCTTCCGATCTCTCCACCTTCTCCTCATCTC
gRNA-a-off target-2	aoff-2F	TCTACACTCTTTCCCTACACGACGCTCTTCCGATCTAACCTGGGTCTCATCTCGGT	aoff-2R	GTGACTGGAGTTCAGACGTGTGCTCTTCCGATCTGCTTCTCGCCAGCCTCCTTG
gRNA-a-on site	aon-1F	TCTACACTCTTTCCCTACACGACGCTCTTCCGATCTTGGCTGCTCTGGGGGCCTCC	aon-1R	GTGACTGGAGTTCAGACGTGTGCTCTTCCGATCTGGGAGCCCTTCTTCTTCTGC
gRNA-b-off target-1	boff-1F	TCTACACTCTTTCCCTACACGACGCTCTTCCGATCTCCCAGTTGTTGATGGGTTTG	boff-1R	GTGACTGGAGTTCAGACGTGTGCTCTTCCGATCTCTGGTGTAAACTGATCTATC
gRNA-b-on site	bon-1F	TCTACACTCTTTCCCTACACGACGCTCTTCCGATCTTGTGAATGTTAGACCCATGG	bon-1R	GTGACTGGAGTTCAGACGTGTGCTCTTCCGATCTCCAGGGAGGGAGGGGCACAG

Supplementary Table 12| Information in Supplementary Figure 11

gRNA sequence	PAM position 1-7	Forward primer		Reverse primer	
GAACTTGGATCCGAGCGGAGG	gtggatg	SF11-1F	CTTTCCCTACACGACGCTCTTCCGATCTTCACACCCGGTGTGTAGCTCAGCCTA	SF11-1R	GAGTTCAGACGTGTGCTCTTCCGATCTTGTCTTGGAGAGTCCCGGAGAGCAGG
TCTGTAGCTCACTGAAGGCTT	aagaatt	SF11-2F	CTTTCCCTACACGACGCTCTTCCGATCTCAGAAGCAGGTTGTAAAATGAAGTGG	SF11-2R	GAGTTCAGACGTGTGCTCTTCCGATCTATGATATAAAGAAGCTCTCATTATTA
TTAAGAATTTGGGCTTTATGG	gcaagcc	SF11-3F	TCTACACTCTTTCCCTACACGACGCTCTTCCGATCTAAAGTGAGGTTCAGCAA	SF11-3R	GTGACTGGAGTTCAGACGTGTGCTCTTCCGATCTGGGCTTCCTACTGTC
GAAGCAGCTCCAGTGCCCAGA	cacggtg	SF11-4F	TCTTTCCCTACACGACGCTCTTCCGATCTCGATGTAAGGCGAGGGGGTGGATCTC	SF11-4R	ACTGGAGTTCAGACGTGTGCTCTTCCGATCTACTTGACTTCACAAGGGGCCAGGCGG
TCCGAGCGGAGGGTGGATGTT	tggggtc	SF11-5F	TCTTTCCCTACACGACGCTCTTCCGATCTCGATGTGGAGAGTCCCGGAGAGCAGG	SF11-5R	ACTGGAGTTCAGACGTGTGCTCTTCCGATCTTTAGGCGCCTAACTCTCCCATTCGTC
AGGTGTGTATTCCAGAATTGA	agcaaag	SF11-6F	TACACTCTTTCCCTACACGACGCTCTTCCGATCTAGTCATGCCCTTCAGCACAAGTTAC	SF11-6R	GACTGGAGTTCAGACGTGTGCTCTTCCGATCTTCCGAGATTGTTTATTGATGATTA

Supplementary Table 13 | DNA/protein sequences

Name	DNA/protein sequence	Description
SaCas9-KKH	MPKKKRKVGGGSPGGGGSKRNYILGLDIGITSVGYGIIDYETRDVIDAGVRLFKEANVENNEGRRSKRGARRLKRRRRHRIQR<br>VKKLLFDYNLLTDHSELSGINPYEARVKGLSQKLSEEEFSAALLHLAKRRGVHNVNEVEEDTGNELSTKEQISRNSKALEEKYV<br>AELQLERLKKDGEVRGSINRFKTSDYVKEAKQLLKVQKAYHQLDQSFIDTYIDLLETRRTYYEGPGEGSPFGWKDIKEWYEML<br>MGHCTYFPEELRSVKYAYNADLYNALNDLNNLVITRDENEKLEYYEKFQIIENVFKQKKKPTLKQIAKEILVNEEDIKGYRVTST<br>GKPEFTNLKVYHDIKDITARKEIIENAELLDQIAKILTIYQSSEDIQEELTNLNSELTQEEIEQISNLKGYTGTHNLSLKAINLILDEL<br>WHTNDNQIAIFNRLKLVPKKVDLSQQKEIPTTLVDDFILSPVVKRSFIQSIKVINAIIKKYGLPNDIIIELAREKNSKDAQKMINE<br>MQKRNRQTNERIEEIIRTTGKENAKYLIEKIKLHDMQEGKCLYSLEAIPLEDLLNNPFNYEVDHIIPRSVSFDNSFNNKVLVKQE<br>ENSKKGNRTPFQYLSSSDSKISYETFKKHILNLAKGKGRISKTKKEYLLEERDINRFSVQKDFINRNLVDTRYATRGLMNLLRSYF<br>RVNNLDVKVKSINGGFTSFLRRKWKFKKERNKGYKHHAEDALIIANADFIFKEWKKLDKAKKVMENQMFEEKQAESMPEIET<br>EQEYKEIFITPHQIKHIKDFKDYKYSHRVDKKPNRKLINDTLYSTRKDDKGNTLIVNNLNGLYDKDNDKLKKLINKSPEKLLMYH<br>HDPQTYQKLKLIMEQYGDEKNPLYKYYEETGNYLTKYSKKDNGPVIKKIKYYGNKLNAHLDITDDYPNSRNKVVKLSLKPYRF<br>DVYLDNGVYKFVTVKNLDVIKKENYYEVNSKCYEEAKKLKKISNQAEFIASFYKNDLIKINGELYRVIGVNNDLLNRIEVNMIDI<br>TYREYLENMNDKRPPHIIKTIASKTQSIKKYSTDILGNLYEVKSKKHPQIIKKGTSGGGSKRPAATKKAGQAKKKKSR	Green:NLS

inactive EYFP reporter gene	ATGGTGAGCAAGGGCGAGGAGCTGTTCACCGGGGTGGTGCCCATCCTGGTCGAGCTGGACGGCGACGTAAACGGC<br>CACAAGTTCAGCGTGTCCGGCGAGGGCGAGGGCGATGCCACCTACGGCAAGCTGACCCTGAAGTTCATCTGCACCA<br>CCGGCAAGCTGCCCGTGCCCTGGCCCACCCTCGTGACCACCTTCGGCTACGGCCTGCAGTGCTTCGCCCGCTACCCC<br>GACCACATGAAGCAGCACGACTTCTTCAAGTCCGCCATGCCCGAAGGCTACGTCCAGGAGCGCACCATCTTCTTCAA<br>GGACGACGGCAACTACAAGACCCGCGCCGAGGTGAAGTTCGAGGGCGACACCCTGGTGAACCGCATCGAGCTGAA<br>GGGCATCGACTTCAAGGAGGACGGCAACATCCTGGGGCACAAGCTGGAGTACAACTACAACAGCCACAACGTCTAT<br>ATCATGGCCGACAAGCAGAAGAACGGCATCAAGGTGAACTTCAAGATCCGCCACAACATCGAGGACGGCAGCGTGC<br>AGCTCGCCGACCACTACCAGCAGAACACCCCCATCGGCGACGGCCCCGTGCTGCTGCCCGACAACCACTACCTGAG<br>CTACCAGTCCAAGCTGAGCAAAGACCCCAACGAGAAGCGCGATTAACCTAGATACGTTCTCTATCACTGATACCNNN<br>NGCCTGTGCTTCGCCCGCTACCCCGACCACATGAAGCAGCACGACTTCTTCAAGTCCGCCATGCCCGAAGGCTACGT<br>CCAGGAGCGCACCATCTTCTTCAAGGACGACGGCAACTACAAGACCCGCGCCGAGGTGAAGTTCGAGGGCGACACC<br>CTGGTGAACCGCATCGAGCTGAAGGGCATCGACTTCAAGGAGGACGGCAACATCCTGGGGCACAAGCTGGAGTACA<br>ACTACAACAGCCACAACGTCTATATCATGGCCGACAAGCAGAAGAACGGCATCAAGGTGAACTTCAAGATCCGCCA<br>CAACATCGAGGACGGCAGCGTGCAGCTCGCCGACCACTACCAGCAGAACACCCCCATCGGCGACGGCCCCGTGCT<br>GCTGCCCGACAACCACTACCTGAGCTACCAGTCCAAGCTGAGCAAAGACCCCAACGAGAAGCGCGATCACATGGTC<br>CTGCTGGAGTTCGTGACCGCCGCCGGGATCACTCTCGGCATGGACGAGCTGTACAAG	Blue:gRNA spacer sequence<br>Red:3th-6th PAM sequence

dSaCas9-KKH:VPR	MPKKKRKVGGGSPGGGGSKRNYILGLAIGITSVGYGIIDYETRDVIDAGVRLFKEANVENNEGRRSKRGARRLKRRRRHRIQR<br>VKKLLFDYNLLTDHSELSGINPYEARVKGLSQKLSEEEFSAALLHLAKRRGVHNVNEVEEDTGNELSTKEQISRNSKALEEKYV<br>AELQLERLKKDGEVRGSINRFKTSDYVKEAKQLLKVQKAYHQLDQSFIDTYIDLLETRRTYYEGPGEGSPFGWKDIKEWYEML<br>MGHCTYFPEELRSVKYAYNADLYNALNDLNNLVITRDENEKLEYYEKFQIIENVFKQKKKPTLKQIAKEILVNEEDIKGYRVTST<br>GKPEFTNLKVYHDIKDITARKEIIENAELLDQIAKILTIYQSSEDIQEELTNLNSELTQEEIEQISNLKGYTGTHNLSLKAINLILDEL<br>WHTNDNQIAIFNRLKLVPKKVDLSQQKEIPTTLVDDFILSPVVKRSFIQSIKVINAIIKKYGLPNDIIIELAREKNSKDAQKMINE<br>MQKRNRQTNERIEEIIRTTGKENAKYLIEKIKLHDMQEGKCLYSLEAIPLEDLLNNPFNYEVDHIIPRSVSFDNSFNNKVLVKQE<br>EASKKGNRTPFQYLSSSDSKISYETFKKHILNLAKGKGRISKTKKEYLLEERDINRFSVQKDFINRNLVDTRYATRGLMNLLRSYF<br>RVNNLDVKVKSINGGFTSFLRRKWKFKKERNKGYKHHAEDALIIANADFIFKEWKKLDKAKKVMENQMFEEKQAESMPEIET<br>EQEYKEIFITPHQIKHIKDFKDYKYSHRVDKKPNRKLINDTLYSTRKDDKGNTLIVNNLNGLYDKDNDKLKKLINKSPEKLLMYH<br>HDPQTYQKLKLIMEQYGDEKNPLYKYYEETGNYLTKYSKKDNGPVIKKIKYYGNKLNAHLDITDDYPNSRNKVVKLSLKPYRF<br>DVYLDNGVYKFVTVKNLDVIKKENYYEVNSKCYEEAKKLKKISNQAEFIASFYKNDLIKINGELYRVIGVNNDLLNRIEVNMIDI<br>TYREYLENMNDKRPPHIIKTIASKTQSIKKYSTDILGNLYEVKSKKHPQIIKKGTSRADPKKKRKVEASGSGRADALDDFDLDML<br>GSDALDDFDLDMLGSDALDDFDLDMLGSDALDDFDLDMLINSRSQYLPDTDDRHRIEEKRKRTYETFKSIMKKSPFSGPTDP<br>RPPPRRIAVPSRSSASVPKPAPQPYPFTSSLSTINYDEFPTMVFPSGQISQASALAPAPPQVLPQAPAPAPAPAMVSALAQAP<br>APVPVLAPGPPQAVAPPAPKPTQAGEGTLSEALLQLQFDDEDLGALLGNSTDPAVFTDLASVDNSEFQQLLNQGIPVAPHT<br>TEPMLMEYPEAITRLVTGAQRPPDPAPAPLGAPGLPNGLLSGDEDFSSIADMDFSALLGSGSGSRDSREGMFLPKPEAGSAIS<br>DVFEGREVCQPKRIRPFHPPGSPWANRPLPASLAPTPTGPVHEPVGSLTPAPVPQPLDPAPAVTPEASHLLEDPDEETSQAV<br>KALREMADTVIPQKEEAAICGQMDLSHPPPRGHLDELTTTLESMTEDLNLDSPLTPELNEILDTFLNDECLLHAMHISTGFSIF<br>DTSLF	NLS VPR

dV42:VPR	MPKKKRKVGGGSPGGGGSKRNYILGLAIGITSVGYGIIDYETRDVIDAGVRLFKEANVENNEGRRSKRGARRLKRRRRHRIQR<br>VKKLLFDYNLLTDHSELSGINPYEARVKGLSQKLSEEEFSAALLHLAKRRGVHNVNEVEEDTGNELSTKEQISRNSKALEEKYV<br>AELQLERLKKDGEVRGSINRFKTSDYVKEAKQLLKVQKAYHQLDQSFIDTYIDLLETRRTYYEGPGEGSPFGWKDIKEWYEML<br>MGHCTYFPEELRSVKYAYNADLYNALNDLNNLVITRDENEKLEYYEKFQIIENVFKQKKKPTLKQIAKEILVNEEDIKGYRVTST<br>GKPEFTNLKVYHDIKDITARKEIIENAELLDQIAKILTIYQSSEDIQEELTNLNSELTQEEIEQISNLKGYTGTHNLSLKAINLILDEL<br>WHTNDNQIAIFNRLKLVPKKVDLSQQKEIPTTLVDDFILSPVVKRSFIQSIKVINAIIKKYGLPNDIIIELAREKNSKDAQKMINE<br>MQKRNRQTNERIEEIIRTTGKENAKYLIEKIKLHDMQEGKCLYSLEAIPLEDLLNNPFNYEVDHIIPRSVSFDNSFNNKVLVKQE<br>EASKKGNRTPFQYLSSSDSKISYETFKKHILNLAKGKGRISKTKKEYLLEERDINRFSVQKDFINRNLVDTRYATRGLMNLLRSYF<br>RVNNLDVKVKSINGGFTSFLRRKWKFKKERNKGYKHHAEDALIIANADFIFKEWKKLDKAKKVMENQMFEEKQAESMPEIET<br>EQEYKEIFITPHQIKHIKDFKDYKYSHRVDKKPNRKLINDTLYSTRKDDKGNTLIVNNLNGLYDKDNDKLKKLINKSPEKLLMYH<br>HDPQTYQKLKLIMEQYGDEKNPLYKYYEETGNYLTKYSKKDNGPVIKKIKYYGNKLNAHLDITDDYPNSRNKVVKLSLKPYRF<br>DVYLDNGVYKFVTVKNLDVIKKENYYEVNSKCYEEAKKLKKISNQAEFIASFYKNDLIKINGELYRVIGVNNNRLNKIELNMIDI<br>TYREYLENMNDKRPPHIIKTIASKTQSIKKYSTDILGNLYEVKSKKHPQIIKKGTSRADPKKKRKVEASGSGRADALDDFDLDML<br>GSDALDDFDLDMLGSDALDDFDLDMLGSDALDDFDLDMLINSRSQYLPDTDDRHRIEEKRKRTYETFKSIMKKSPFSGPTDP<br>RPPPRRIAVPSRSSASVPKPAPQPYPFTSSLSTINYDEFPTMVFPSGQISQASALAPAPPQVLPQAPAPAPAPAMVSALAQAP<br>APVPVLAPGPPQAVAPPAPKPTQAGEGTLSEALLQLQFDDEDLGALLGNSTDPAVFTDLASVDNSEFQQLLNQGIPVAPHT<br>TEPMLMEYPEAITRLVTGAQRPPDPAPAPLGAPGLPNGLLSGDEDFSSIADMDFSALLGSGSGSRDSREGMFLPKPEAGSAIS<br>DVFEGREVCQPKRIRPFHPPGSPWANRPLPASLAPTPTGPVHEPVGSLTPAPVPQPLDPAPAVTPEASHLLEDPDEETSQAV<br>KALREMADTVIPQKEEAAICGQMDLSHPPPRGHLDELTTTLESMTEDLNLDSPLTPELNEILDTFLNDECLLHAMHISTGFSIF<br>DTSLF	NLS VPR

Supplementary Table 14 | primers used in quantitative PCR

β-actin-F	CATGTACGTTGCTATCCAGGC
β-actin-R	CTCCTTAATGTCACGCACGAT
IL1RN-F	GGAATCCATGGAGGGAAGAT
IL1RN-R	TGTTCTCGCTCAGGTCAGTG

Supplementary References

1. Ma, D., Peng, S., Huang, W., Cai, Z. & Xie, Z. Rational Design of Mini-Cas9 for Transcriptional Activation. *ACS Synth. Biol.* **7,** 978–985 (2018).
2. Chen, B. *et al.* Expanding the CRISPR imaging toolset with Staphylococcus aureus Cas9 for simultaneous imaging of multiple genomic loci. *Nucleic Acids Res.* **44,** e75 (2016).
3. Crooks, G. E., Hon, G., Chandonia, J. & Brenner, S. E. WebLogo: a sequence logo generator. *Genome Res.* **14,** 1188–90 (2004).
