# Supplementary material for: Engineer chimeric Cas9 to expand PAM recognition based on evolutionary information
Source: Nat Commun. 2019 Feb 4;10:560. doi: 10.1038/s41467-019-08395-8 (PMC6361995; doi:10.1038/s41467-019-08395-8)
Supplement: Supplementary file 3 — Description of Additional Supplementary Files [file 41467_2019_8395_MOESM3_ESM.pdf]

Description of Additional Supplementary Information

File Name: Source Data

Description: The raw data underlying Figs 1d, 2b-d, 3b-c, 4a-c and Supplementary Figs 2c-d, 3-11 is listed in the Source Data file.
