# Supplementary material for: Engineer chimeric Cas9 to expand PAM recognition based on evolutionary information
Source: Nat Commun. 2019 Feb 4;10:560. doi: 10.1038/s41467-019-08395-8 (PMC6361995; doi:10.1038/s41467-019-08395-8)
Supplement: Supplementary file 5 — Reporting Summary [file 41467_2019_8395_MOESM5_ESM.pdf]

Reviewers' comments:

Reviewer #1 (Remarks to the Author):

The manuscript by Ma et al describes a series of chimeric Staphylococcus aureus Cas9s, generated by replacing residues in a region of the PAM interacting domain of the KKH expanded PAM SaCas9 variant with those corresponding to various SaCas9 orthologs. The authors show that some of the resulting chimeras have expanded PAM recognition in both a mammalian fluorescent reporter system and, to a lesser extent, a T7E1 DNA digestion assay of genomic DNA. Although the approach is interesting, the heavy reliance on reporter data, incomplete assessments of the chimeric Cas9s, and inconsistencies in the data shown call into question the claims made by the authors. Overall, this manuscript is not suitable for publication in Nature Communications.

Major concerns:

Overall, fluorescent reporters and T7EI assays are not sufficient to prove that these new cCas9 variants can access RRN PAMs on the genome better than KKH-SaCas9. The genome editing field has largely moved away from T7EI assays because they have been shown to be prone to report artifacts such as binding without cutting. Data is not internally consistent between the fluorescent reporter and those values reported in the T7EI assay for PAM accessibility, and the values for KKH-SaCas9 are far too high in T7EI on non NNNRRT PAM sites (Supp figure 3a). All experiments are performed on a non-validated optimized gRNA.

The type of replicates in the figures is either not stated or is ambiguously stated – what is an "independent replicate?"

Optimized sgRNA does not seem to increase on-target activity a lot (SaKKH Cas9 on NNNRRT PAMs) but drastically increases activity on off-target sites (e.g., NNNRRN increases a lot). What consequence does this optimized sgRNA have on the already well characterized KKH SaCas9 (raw editing numbers by HTS, off targets, etc.). Do these constructs only work with this optimized sgRNA that increases non-canonical activity?

KKH R991K and R991K/D987N do appear relax the preference to the 6th position, but with the downside of decreased on-target activity.

In figure 1, the authors claim 2/3 of all 32 cCas9 variants display a different PAM recognition pattern but this claim seems like an exaggeration because 1/3 of the cCas9s don't seem to have any activity and another 8 don't have activity on any PAM comparable to KKH on its NNNRRT PAMs, calling into question if they are actually active. Also, the true background of this reporter assay on all 64 PAMs (for instance, the fluorescence obtained by transfection with nuclease-inactive Cas9) should be provided for comparison.

The dynamic range of the EYFP reporter appears to be at best 64-fold (6 units of log base 2). Given the inconsistency between some of the data, this limited range calls into question the ability of this assay to distinguish between true instances of improved PAM recognition. It is also interesting that the T7E1 DNA cleavage assays appear to show a lower or zero difference between the cCas9s and KKH compared to the EYFP reporter assay (even at 3 days).

In figure 2e (and figure 3e), some of the fluorescent values in the bar graphs don't correspond well to those from the heat map, although the text implies they were obtained using the same assay. For example, the intensity of V17K on AGC is ~ 4 in (e) and looks to be ~8 fold higher than KKH, but looking at the heatmap in part (d) V17K on AGC is ~ 2 and seems very close to the value for KKH on AGC in the heatmap in part (b). Yet the error bars in (e) look quite small (but the type of replicate is not clear). This inconsistency is troubling given that much of the paper's claims are derived from this EYFP assay.

In figure 2f, with the exception of GGC, the values of both KKH and V42 appear to be fairly close to each other. Also, no statistical testing is given to show if these values are actually significantly different. It's doubtful if the case that V42 is acts at a "faster speed" than KKH can be made from this data.

In Supplementary Figure 3a, T7EI is not an accurate measurement for your purposes since it shows high level of KKH indel formation at NNNRRV sites (>20%), which is not representative at all of what occurs on the genome. It also does not correspond to the results in the fluorescent reporter, for example GAC is completely inactive in your fluorescent reporter but shows levels equal to GGT for KKH. Again, the genome editing field has mostly moved past the T7EI assay since that assay, while easy to do, is not reliable for the reasons discussed above.

The 6th base on the x axis of the heat maps in figure 3b-d are inconsistent with figure 2b-d. Is this an error?

Figure 3F data is very erratic in terms of editing efficiencies of your mutants on each PAM, indicating that there might be an additional sequence bias (is it truly NNNRRNN, or are any of those Ns defined?). Create a sequence logo from the efficiencies at these sites to determine. Additionally, statistical testing to show if these variants are significantly different would lend more credence to this data.

Figures 3E and 3F lack a wt on wt control (KKH on an NNNRRT site) do not show all variants on all sites

If the authors want to claim that V21R-HF should be used instead of V21R, they need to demonstrate that V21R-HF can still work on a non-NNNRRT site as well

Supplementary figure 6: these should all be compared directly to the efficiencies of wt KKH Cas9. What are V32 and V35?

Minor comments:

The writing is very uneven, with many grammatical mistakes

A reference for the EYFP reconstitution assay is not given in the text when it is first introduced

In figure 1, the legend on the scale for the heat map is too small to read clearly – especially the base of the Log scale

In figure 2 and 3, the naming of the cCas9 variants is inconsistent between the heat maps and the bar graphs

In figure 3b, the heat map of KKH should be shown for comparison

In figure 3f, what do the error bars represent? Also, since each data point here is the mean of 3 replicates, was the error propagated to obtain these overall error bars?

Reviewer #2 (Remarks to the Author):

The protospacer adjacent motif (PAM) requirement of CRISPR nucleases is one of the most important determinants of targeting range. The smaller Cas9 ortholog from Staphylococcus aureus (SaCas9) recognizes an extended NNGRRN PAM (with a preference for T in the 6th position), and previous work by others showed that the PAM recognition of SaCas9 could be expanded using a directed engineering approach to generate a variant called SaCas9-KKH that targets NNNRRT PAMs. In the present study, Ma et al. describe an approach where they seek to expand the PAM recognition capabilities of SaCas9-KKH by generating chimeric SaCas9-KKH variants that bear different 13 amino acid stretches from other Cas9 orthologs (in a region of the PAM-interacting domain that is thought to be important for PAM base readout). The approach is interesting and potentially useful, but unfortunately the results are severely under-sampled and thus do not justify the conclusions, the comparisons are not performed with appropriate controls (ex. to wild-type SaCas9), and there are many missing data sets. This is compounded by a meandering narrative that is not adequately explained by the authors. The authors can remedy many of these critiques by adding much more data to support their claims.

Major comments:

1) One major omission is the complete lack of comparison to wild-type SaCas9. The authors should include data for WT SaCas9 in many relevant areas, especially on sites that contain PAMs that SaCas9 is known to be more potent than SaCas9-KKH (NNGRRV). For example, data for wild-type SaCas9 should be included in Fig. 1, Figs. 2B, 2E, and 2F; Supplementary Fig. 3 (especially for the endogenous site targeting, as many of these sites should be more potently targeted with SaCas9 vs. SaCas9-KKH). This also begs the larger question of why the authors moved the 13 aa stretch from orthologs into SaCas9-KKH instead of into wild-type SaCas9? For some of their prominent variants, the authors should move the 13aa sequence into wild-type SaCas9 and examine the PAM preferences of these variants. No comparison to wild-type SaCas9? It's more potent on NNGRRT and NNGRRV PAMs…! Why didn't the authors move the 13aa stretch into WT? Why was WT SaCas9 omitted from Figure 1D? And Figures 2b-2e?

2) Another major issue with this study is the undersampling of sites to justify their claims. Conclusions are often based on the results from a comparison on one target site per PAM – this is not a robust enough data set to draw meaningful conclusions. The authors should include more sites per PAM that they are claiming are now accessible with their variants, otherwise it's impossible to tell whether these sites were intentionally chosen because they showed the intended outcome.

3) As the authors are aware, the SaCas9 PAM is 6 nucleotides. This reviewer appreciates the desire for brevity and reducing the PAM to stating only positions 4-6, but in many cases this is confusing. The authors should make it abundantly clear what the nucleotides in positions 1-3 are. In

many cases, the 3rd PAM position should be explicitly stated, since the context of the whole PAM is important for activity. The full PAM sequence should be included for all endogenous target sites, and the nucleotides in positions 1-3 should be included in the schematic of Fig. 1C (if the sequence is indeed consistent across all EYFP experiments).

4) Related to point #2, in supplementary Table 2, it looks like the authors are using a CCCNNN PAM for their EYFP experiments. Why did the authors choose a static PAM with a C in the 3rd position? Using a G in the 3rd position would have enabled a comparison to wild-type SaCas9, and the authors should therefore make it clear why they chose a C in the 3rd position. This is a major flaw in their experimental design that must be addressed.

5) The authors need to be much more careful when making comparative statements to the KKH variant. For example, “These results suggested that cCas9 V42 had an enhanced PAM recognition capability than SaCas9-KKH” implies that the cV42 variant is more active than KKH on the PAMs that the KKH variant is known to target (NNNRRT). There are only two RRT PAMs in Supp. Fig. 3B, and KKH performs equal to cV42 on one site and almost ten-fold better on the other. The authors should use a word like “expanded” instead of enhanced, as the cV42 is not enhanced compared to KKH. Additionally, the authors should indicate in all of their comparisons to KKH which PAMs are expected to be targeted by KKH, and which are not (based on the NNNRRT PAM preference).

6) The manuscript would benefit from substantial English-language editing. There are numerous grammatical mistakes throughout (prominently in the title and in the abstract). Also, the title implies something different than what the study delivers (since the variants aren’t really enhanced), so the title should be revised accordingly.

7) The authors should re-plot the KKH data beside the KKH(N968S) variant data in Fig. 3D. Also, the authors should probably name the KKH(N968S) variant as KSH, since they changed an amino acid that is contained within the KKH variant. Furthermore, the KSH variant data should be plotted in Fig. 3E, and also experiments should be performed with this variant for the equivalent sites in Fig. 3F and Supplementary Figure 4. From their assay data in Fig. 3D, the KSH variant looks like it should be the most active, so this is a notable omission not to include it in the most relevant experiments.

8) The on-target editing comparisons between KKH, V21R, and V21R-HF in Figure 4 should be expanded. At the one site examined, there is a clear loss of on-target activity, so the authors should compare the on-target activities on 10-12 on-target sites at a minimum if this is a major point of their manuscript (to justify including it as a main figure).

9) In the text, the rationale for switching from chimeric variants to orthologs is not clear, and the statement in the manuscript “Next, we tested the gene editing activity of these SaCas9 orthologs in mammalian cells.” does not indicate why. In Supplementary Figures 6B and 6C, it looks like the KKH variants of O32 and O35 actually decrease activity compared to V32 and V35. The authors should include experiments to compare the activities of wild-type O32 and O35 in Supplementary Figure 6D to O32-KKH and O35-KKH. Also, what crRNA/tracrRNAs are used in these experiments, are they the optimal one for the variant/ortholog?

10) In Supplementary Figure 2, the gRNA scaffold optimization is very interesting. The authors should perform identical experiments with wild-type SaCas9 to determine if they observe a concomitant increase in activity and targeting range with the optimized sgRNA scaffold. Furthermore, in Figure 2B and Supplementary Figure 2C, where are the results for optimized sgRNA-1? And is optimized sgRNA-1 the previously described one from ref. 25? This should be clarified.

Minor comments:

1) It would be very helpful if the authors could make the order of orthologs consistent between Figs. 1B and 1D.

2) The “cCas9 V17K and cCas9 V17L” nomenclature is confusing, and implies a mutation at Valine 17. Perhaps the authors can use more complete names (v17-I991L) or consider making the “V” lowercase (v17-L)?

3) In Supplementary Figure 2A, the authors should indicate from which strain each crRNA is derived from.

4) What PAMs do the other orthologs in this study naturally target (can the authors predict bioinformatically from the crRNA sequences in the endogenous CRISPR locus)?

5) Why do the authors wait 8 days before extracting genomic DNA and performing the T7E1 assay? Almost all other genome editing studies use a 3-day time point.

6) I believe that the 6th position of the PAM is mislabeled in Figure 3, as order of PAMs between the plots of Figure 2B-2D compared to 3B-3D (in the 6th position, ACGT vs. ATCG, respectively) changes (and doesn’t seem correct in Figure 3).

7) In Supplementary Figure 5, the authors should perhaps generate the alignment with SaCas9 instead of SaCas9-KKH and simply indicate the positions where the KKH substitutions are found.

8) In Supplementary Table 2, it would be helpful for the authors to indicate important parts of the polypeptide sequences (for example, what sequence is the NLS, VPR, etc.)

9) Certain statements are lacking citations, such as: “By using our previous EYFP fluorescence reconstitution assay in HEK293FT cells”

Reviewers' comments:

Reviewer #1 (Remarks to the Author):

The manuscript by Ma et al describes a series of chimeric Staphylococcus aureus Cas9s, generated by replacing residues in a region of the PAM interacting domain of the KKH expanded PAM SaCas9 variant with those corresponding to various SaCas9 orthologs. The authors show that some of the resulting chimeras have expanded PAM recognition in both a mammalian fluorescent reporter system and, to a lesser extent, a T7E1 DNA digestion assay of genomic DNA. Although the approach is interesting, the heavy reliance on reporter data, incomplete assessments of the chimeric Cas9s, and inconsistencies in the data shown call into question the claims made by the authors. Overall, this manuscript is not suitable for publication in Nature Communications.

Major concerns:

1.1 Overall, fluorescent reporters and T7EI assays are not sufficient to prove that these new cCas9 variants can access RRN PAMs on the genome better than KKH-SaCas9. The genome editing field has largely moved away from T7EI assays because they have been shown to be prone to report artifacts such as binding without cutting. Data is not internally consistent between the fluorescent reporter and those values reported in the T7EI assay for PAM accessibility, and the values for KKH-SaCas9 are far too high in T7EI on non NNNRRT PAM sites (Supp figure 3a). All experiments are performed on a non-validated optimized gRNA.

A1.1: We thank the reviewer for this comment. To more robustly evaluate the activity of the chimeric Cas9 variants, SaCas9-KKH and SaCas9 by using the fluorescent reporter assay, we performed dosage experiments in Fig. 2c and Fig. 4a. Based on these results, we hypothesized that oversaturation may be the reason for the observation that the values for

KKH-SaCas9 were too high in T7EI on non-NNNRRT PAM sites.

As shown in Fig. 2d, Fig. 4b, Fig. 4c, Supplementary Fig. 5, Supplementary Fig. 6, Supplementary Fig. 8b, Supplementary Fig. 10, and Supplementary Fig. 11, all the endogenous gene editing assays in the revised manuscript have been measured by using the next generation sequencing. These results collectively showed that the chimeric Cas9 variants has expanded PAM recognition as stated on Page 6 and Page 8 in the manuscript.

To evaluate the activity of the optimized gRNA, we compared the activity of SaCas9 and SaCas9-KKH at CCGRRN with a guanine at the third position and CCCRRN PAMs with a cytosine at the third position, guided with wildtype gRNA or optimized gRNA-2. We observed an increased activity at CCCRRV PAMs induced by SaCas9-KKH and CCGRRV PAMs induced by SaCas9 when directed with the optimized gRNA-2 (Supplementary Fig. 2d).

In addition, as shown in Supplementary Fig. 10, v21-R-HF displayed significantly decreased rates of mutagenesis at two out of three endogenous off-target sites containing one point-mutation in the spacer sequences when directed by either wildtype gRNA or optimized gRNA-2.

1.2 The type of replicates in the figures is either not stated or is ambiguously stated – what is an "independent replicate?"

A1.2: We thank the reviewer for this comment. The independent replicate means that we performed biological independent experiments for indicated repeats. Each biological independent experiment represents one independent transfection assay. To avoid misleading, we added the following description in the Methods section "…Each transfection experiment was independently….".

1.3 Optimized sgRNA does not seem to increase on-target activity a lot (SaKKH Cas9 on NNNRRT PAMs) but drastically increases activity on off-target sites (e.g., NNNRRN increases a lot). What consequence does this optimized sgRNA have on the already well characterized KKH SaCas9 (raw editing numbers by HTS, off

targets, etc.). Do these constructs only work with this optimized sgRNA that increases non-canonical activity?

A1.3: We thank the reviewer for this comment. We agree with the reviewer that the optimized gRNA has a stronger effect on increasing the activity of SaCas9 and SaCas9-KKH on target sites with NNNRRV PAMs compared to target sites with NNNRRT PAMs (Supplementary Fig. 2c and 2d). Then, we decided to use the optimized gRNA in our study. In addition, we also showed that cCas9 variants, SaCas9 and SaCas9-KKH displayed a comparable activity at endogenous sites when guided by both the wild-type gRNA and the optimized gRNA-2 (Supplementary Fig. 10).

1.4 KKH R991K and R991K/D987N do appear relax the preference to the 6th position, but with the downside of decreased on-target activity.

A1.4: We thank the reviewer for this comment. In the discussion part, we added with this description "... It is intriguing that although the v42, v17-L, SaCas9-KKH R991K and SaCas9-KKH R991K/D987N showed expanded activities at NNVRRV PAMs, these variants displayed decreased activities on NNVRRT PAMs, which is consistent with the previous report that the SaCas9-KKH showed decreases activities at NNGRRT PAMs. One explanation is that sufficient PAM binding activity of SaCas9 nucleases may be required to initiate strong gene editing activities and relaxed PAM binding activity of SaCas9 nucleases results in reduced DNA cleavage activity. Further studies are necessary to fully understand the functional relationship between the PAM recognition and the SaCas9 nuclease activity…."

1.5 In figure 1, the authors claim 2/3 of all 32 cCas9 variants display a different PAM recognition pattern but this claim seems like an exaggeration because 1/3 of the cCas9s don't seem to have any activity and another 8 don't have activity on any PAM comparable to KKH on its NNNRRT PAMs, calling into question if they are actually active. Also, the true background of this reporter assay on all 64 PAMs (for instance, the fluorescence obtained by transfection with nuclease-inactive

Cas9) should be provided for comparison.

A1.5: We thank the reviewer for this suggestion. We revised the description "…2/3 of all 32 cCas9 variants display a different PAM recognition pattern…" to "…We found that several cCas9 variants displayed a different PAM recognition pattern from SaCas9-KKH (Fig. 1d)…." in the manuscript. In addition, we added the results of nuclease-inactive Cas9 (dCas9) in the reporter assay as the negative control (Fig. 1c).

1.6 The dynamic range of the EYFP reporter appears to be at best 64-fold (6 units of log base 2). Given the inconsistency between some of the data, this limited range calls into question the ability of this assay to distinguish between true instances of improved PAM recognition. It is also interesting that the T7E1 DNA cleavage assays appear to show a lower or zero difference between the cCas9s and KKH compared to the EYFP reporter assay (even at 3 days).

A1.6: We thank the reviewer for this comment. We performed the EYFP reporter assay in a wide range of plasmid doses of different Cas9 variants (Fig. 2c and Fig. 4a), and these dosage results were highly consistent to each other (Fig. 2a, Fig. 3, Supplementary Fig. 4 and Supplementary Fig. 9). We also measured the activities of different Cas9 variants by using deep sequencing analysis (Fig. 2d, Fig. 4b, Fig. 4c, Supplementary Fig. 5, Supplementary Fig. 6, Supplementary Fig. 8b, Supplementary Fig. 10 and Supplementary Fig. 11). These results were also well correlated to our dosage results.

1.7 In figure 2e (and figure 3e), some of the fluorescent values in the bar graphs don't correspond well to those from the heat map, although the text implies they were obtained using the same assay. For example, the intensity of V17K on AGC is ~ 4 in (e) and looks to be ~8 fold higher than KKH, but looking at the heatmap in part (d) V17K on AGC is ~ 2 and seems very close to the value for KKH on AGC in the heatmap in part (b). Yet the error bars in (e) look quite small (but the type of replicate is not clear). This inconsistency is troubling given that much of the

paper's claims are derived from this EYFP assay.

A1.7: We thank the reviewer for this comment. To more robustly compare the activities of these variants, we performed the EYFP reporter assay in eight different doses of cCas9 variant (v42 and v21) plasmids (Fig. 2c and Fig.4a). Because the cCas v42 and v21 are our main focuses, we moved the results of cCas9 v17 into Supplementary Figure 8. In the revised manuscript, we described the results on Page 6 as following: "Interestingly, the cCas9 v17 with either an I991K or I991L mutation (cCas9 v17-K and v17-L) expanded the activity on targets containing CCCRRN PAMs (Supplementary Fig. 8a). To compare the activity of v17-L, v42 and SaCas9-KKH when targeting endogenous target sites in HEK293FT cells, we performed the deep sequencing analysis on the indel frequency at 37 different endogenous target sites with NNNRRV PAMs. We observed that v17-L displayed about half of the sites showing higher than 5% indels with a mean mutagenesis frequency of 9.5% (Supplementary Fig. 8b and Supplementary Table 3)."

1.8 In figure 2f, with the exception of GGC, the values of both KKH and V42 appear to be fairly close to each other. Also, no statistical testing is given to show if these values are actually significantly different. It's doubtful if the case that V42 is acts at a "faster speed" than KKH can be made from this data.

A1.8: We thank the reviewer for this comment. We performed a dosage experiment to compare the activity of v42 and SaCas9-KKH by using the EYFP reporter assay. We found that cCas9 v42 showed the highest activity at the CCCRRV (V=A, C and G) PAMs over a range of plasmid doses (Fig. 2c). Then, we also chose 77 endogenous sites with NNNRRN PAMs (at least 3 target sites with each PAM) and measured the Cas9 activity by using the next generation sequencing (Fig. 2d). Consistent with the results from the EYFP reporter assay, we demonstrated that cCas9 v42 displayed an enhanced activity at endogenous target site with NNNRRV PAM and a comparable activity on endogenous target site with NNNRRT PAM (Fig. 2d).

In addition, sequence logo derived from sites with more than 5% indel frequency by

cCas9 v42 revealed preference of A, C, and G at the third position and no strong preference at the first two positions (Supplementary Fig. 5b). To further examine whether the KKH trimutation in the chimeric Cas9 indeed expanded the third position of PAM, we selected 33 different endogenous gene target sites containing NNVRRN PAMs (V=A, C, or G). As expected, only the cCas9 v42 showed efficient activity with a mean mutagenesis frequency above 10% across all of the sites, but v42-wildtype, SaCas9 and SaCas9-KKH displayed only basal levels of indels at the NNARRN PAM and low activities with a mean indel frequency less than 6% at the NNCRRN PAM (Supplementary Fig. 6 and Supplementary Table 2).

1.9 In Supplementary Figure 3a, T7EI is not an accurate measurement for your purposes since it shows high level of KKH indel formation at NNNRRV sites (>20%), which is not representative at all of what occurs on the genome. It also does not correspond to the results in the fluorescent reporter, for example GAC is completely inactive in your fluorescent reporter but shows levels equal to GGT for KKH. Again, the genome editing field has mostly moved past the T7EI assay since that assay, while easy to do, is not reliable for the reasons discussed above.

A1.9: We thank the reviewer for this suggestion. In the revised manuscript, at each PAM of NNNRRN, we chose more than 3 endogenous target sites and compare the indel frequency induced by indicated SaCas9 variants by using the next generation sequencing (Fig. 2d and Supplementary Fig. 5). In general, the results of the EYFP reporter assay (Fig. 2b) were highly correlated with the results of indel analysis (Fig. 2d and Supplementary Fig. 5).

1.10 The 6th base on the x axis of the heat maps in figure 3b-d are inconsistent with figure 2b-d. Is this an error?

A1.10: We thank the reviewer for this suggestion. We apologize for this mistake. It has been corrected in our revised manuscript.

1.11 Figure 3F data is very erratic in terms of editing efficiencies of your mutants on each PAM, indicating that there might be an additional sequence bias (is it truly NNNRRNN, or are any of those Ns defined?). Create a sequence logo from the efficiencies at these sites to determine. Additionally, statistical testing to show if these variants are significantly different would lend more credence to this data.

A1.11: We thank the reviewer for this suggestion. We answered a similar question in A1.8. Briefly, we created a sequence logo from sites with more than 5% indel frequency by cCas9 v42 (Supplementary Fig. 5). We found a preference of A, C, and G at the third position and no strong preference at the first two positions (Supplementary Fig. 5b). The paired t-test has been used to evaluate the statistical significance in these results.

1.12 Figures 3E and 3F lack a wt on wt control (KKH on an NNNRRT site) do not show all variants on all sites.

A1.12: We thank the reviewer for this suggestion. To quantitatively evaluate the activity of cCas9 v21-R, v21-L, N986S, SaCas9-KKH, v21-R-wt, v21-L-wt, N986S-wt and SaCas9 across six non-NNNRRN PAMs, we first compared activities of these variants by the EYFP reporter assay across eight plasmid doses (Fig. 4a). Furthermore, as shown in Figure 4b and 4c, we tested 6 different cCas9 variants and three controls (SaCas9, SaCas9-KKH and negative control) on 11 different endogenous sites with the six new PAMs.

1.13 If the authors want to claim that V21R-HF should be used instead of V21R, they need to demonstrate that V21R-HF can still work on a non-NNNRRT site as well

A1.3: We thank the reviewer for this suggestion. In Fig. 4b and 4c, we showed the activity of v21-R-HF across 11 endogenous sites with non-NNNRRN PAMs and another six sites with NNNRRN PAMs in Supplementary Fig. 11. In addition, we measured the activities of v21-R-HF at two groups of on-target and off-target sites in Supplementary Fig. 10. Collectively, these results demonstrated that v21-R-HF can still work on target sites with

both non-NNNRRT and NNNRRN PAMs.

1.14 Supplementary figure 6: these should all be compared directly to the efficiencies of wt KKH Cas9. What are V32 and V35?

A1.14: We thank the reviewer for this suggestion. As the activities of orthologs (SshCas9 and SlCas9) were weak and unrelated to the main idea of the chimeric Cas9, we excluded the results of SshCas9, SlCas9, cCas9 v32 and v35 in the revised manuscript.

Minor comments:

1.15 The writing is very uneven, with many grammatical mistakes

A reference for the EYFP reconstitution assay is not given in the text when it is first introduced

A1.15: We thank the reviewer for careful reading of our manuscript. We added the reference of the EYFP reconstitution assay in the revised manuscript on Page 4: "By using a previous EYFP fluorescence reconstitution assay in HEK293FT cells 8 (Fig. 1c),…". LetPub (www.letpub.com) linguistic assistance was used to help us check writing and grammatical mistakes during the preparation of this manuscript.

1.16 In figure 1, the legend on the scale for the heat map is too small to read clearly – especially the base of the Log scale

A1.16: We thank the reviewer for careful reading of our manuscript. We labeled the heat map figures with a larger font in the main manuscript.

1.17 In figure 2 and 3, the naming of the cCas9 variants is inconsistent between the heat maps and the bar graphs

A1.17: We thank the reviewer for careful reading of our manuscript. It has been corrected in the revision manuscript. We added the mutation description and corresponding abbreviation when it first appeared. Briefly, the chimeric Cas9 based on SaCas9-KKH was named as v+number (for example v42). The chimeric Cas9 based on the wild type SaCas9 scaffold was named as v+number+"-wt" (for example v42-wt). When additional mutations were introduced, we named the variant as v+number+"amino acid mutation" (for example, v17-L).

1.18 In figure 3b, the heat map of KKH should be shown for comparison

A1.18: We thank the reviewer for careful reading of our manuscript. It has been added in the Fig. 3b in the revised manuscript.

1.19 In figure 3f, what do the error bars represent? Also, since each data point here is the mean of 3 replicates, was the error propagated to obtain these overall error bars?

A1.19: We thank the reviewer for careful reading of our manuscript. Each point represents mean indel frequency of three biological repeats at one endogenous target site. Thick black line indicated the mean of all target sites. To avoid misleading, we deleted the boxplot in the revised manuscript and added the following description in the manuscript "…Each point represents mean of one endogenous site (n = 3 independent biological replicates). The black line indicated the mean of all targets. …".

Reviewer #2 (Remarks to the Author):

The protospacer adjacent motif (PAM) requirement of CRISPR nucleases is one of the most important determinants of targeting range. The smaller Cas9 ortholog from Staphylococcus aureus (SaCas9) recognizes an extended NNGRRN PAM (with a preference for T in the 6th position), and previous work by others showed that

the PAM recognition of SaCas9 could be expanded using a directed engineering approach to generate a variant called SaCas9-KKH that targets NNNRRT PAMs. In the present study, Ma et al. describe an approach where they seek to expand the PAM recognition capabilities of SaCas9-KKH by generating chimeric SaCas9-KKH variants that bear different 13 amino acid stretches from other Cas9 orthologs (in a region of the PAM-interacting domain that is thought to be important for PAM base readout). The approach is interesting and potentially useful, but unfortunately the results are severely under-sampled and thus do not justify the conclusions, the comparisons are not performed with
appropriate controls (ex. to wild-type SaCas9), and there are many missing data sets. This is compounded by a meandering narrative that is not adequately explained by the authors. The authors can remedy many of these critiques by adding much more data to support their claims.

Major comments:
2.1 One major omission is the complete lack of comparison to wild-type SaCas9. The authors should include data for WT SaCas9 in many relevant areas, especially on sites that contain PAMs that SaCas9 is known to be more potent than SaCas9-KKH (NNGRRV). For example, data for wild-type SaCas9 should be included in Fig. 1, Figs. 2B, 2E, and 2F; Supplementary Fig. 3 (especially for the endogenous site targeting, as many of these sites should be more potently targeted with SaCas9 vs. SaCas9-KKH). This also begs the larger question of why the authors moved the 13 aa stretch from orthologs into SaCas9-KKH instead of into wild-type SaCas9? For some of their prominent variants, the authors should move the 13aa sequence into wild-type SaCas9 and examine the PAM preferences of these variants. No comparison to wild-type SaCas9? It’s more potent on NNGRRT and NNGRRV PAMs…! Why didn’t the authors move the 13aa stretch into WT? Why was WT SaCas9 omitted from Figure 1D? And Figures 2b-2e?

A2.1: We thank the reviewer for this suggestion. We have included the wildtype SaCas9 in Fig. 1d, Fig. 2c, Fig. 4, Supplementary Fig. 2d, Supplementary Fig. 4, Supplementary Fig. 6 and Supplementary Fig. 9-11. We also constructed the corresponding cCas9 based on the wildtype SaCas9 scaffold, including v42-wt, v21-R-wt, v21-L-wt and N986S-wt. Firstly, we compared the cCas9 v42 and v42-wt to SaCas9 and SaCas9-KKH at CCCRRN and CCGRRN PAMs by using the EYFP reporter assay across a panel of doses (Fig. 2c). In addition, we compared these variants at CCNRRN PAMs by the EYFP reporter assay (Supplementary Fig.4). We described the results in the manuscript as following: "Notably, cCas9 v42 showed the highest activity at the CCCRRV (V=A, C and G) PAMs over a range of plasmid doses (Fig. 2c). …… Collectively, these results suggested that cCas9 v42 had an expanded PAM recognition at the NNVRRV PAMs compared to SaCas9-KKH, with the assumption that the first two nucleotides can be either A, C, G, and T[1]." To further examine whether the KKH trimutation in the chimeric Cas9 indeed expanded the third position of PAM, we compared the activities of SaCas9, SaCas9-KKH, cCas9 v42 and v42-wt by selecting 33 different endogenous gene target sites containing NNVRRN PAMs (V=A, C or G) (Supplementary Fig. 6). We described the results in the manuscript as following: "As expected, only the cCas9 v42 showed efficient activity with a mean mutagenesis frequency above 10% across all of the sites, but v42-wildtype, SaCas9 and SaCas9-KKH displayed only basal levels of indels at the NNARRN PAM and low activity with a mean indel frequency less than 6% at the NNCRRN PAM (Supplementary Fig. 6 and Supplementary Table 2)."

In addition, we tested the activities of varying amount of v21-R, v21-R-wt, v21-L, v21-L-wt, N986S, N986S-wt, SaCas9-KKH, SaCas9 at six non-NNNRRN PAMs by using the EYFP reporter assay. We described the results in the manuscript as following: "By using the fluorescent reporter assay (Fig. 1c), we observed that v21-L and v21-R showed high activities at CCMACT, CCMATG, CCMATT, CCMGCT, CCMGTG and CCMGTT PAMs (M=A or C), while N986S displayed relatively high efficiencies at CCMGTT, CCMATT and CCMACT PAMs (Fig. 4a). Similarly, the cCas9 variants with the wildtype SaCas9 scaffold were highly active at PAM sites with a guanine at the third position (Fig. 4)."

Then, we selected 11 endogenous target sites with the non-NNNRRN PAMs and assayed the activities of different Cas9 variants by using the deep sequencing analysis. We described the results in the manuscript as following: "We observed that the average indel frequencies induced by using v21-R, v21-L, N986S and v21-R-HF were more than 10% when targeting endogenous sites with 6 different PAMs (Fig. 4b, 4c and Supplementary Table 4). Furthermore, chimeric Cas9 variants with the scaffold of either wildtype SaCas9 or SaCas9-KKH displayed higher level of indels than SaCas9-KKH at sites of novel PAMs with a guanine at the third position (Fig. 4c and Supplementary Table 4)." In addition, we also confirmed that both v21-L and v21-R efficiently induced indels when targeting endogenous sites with NNVRRN PAMs (Supplementary Fig. 11).

These results showed that the chimeric Cas9 strategy is also suitable to the wildtype SaCas9. The chimeric Cas9 based on wildtype SaCas9 scaffold achieved the highest activities at NNGRRV PAMs and the second highest activities at NNGRRT PAMs (Fig. 2c and Fig. 4a).

2.2 Another major issue with this study is the undersampling of sites to justify their claims. Conclusions are often based on the results from a comparison on one target site per PAM – this is not a robust enough data set to draw meaningful conclusions. The authors should include more sites per PAM that they are claiming are now accessible with their variants, otherwise it's impossible to tell whether these sites were intentionally chosen because they showed the intended outcome.

A2.2: We thank the reviewer for this comment. To more comprehensively compare the activities of v42 and SaCas9-KKH, as shown in Fig. 2d, we chose totally 77 different gRNAs with more than 3 gRNAs per NNNRRN PAM. In Supplementary Fig. 6, we further chose 33 different endogenous sites to compare the nuclease activities of v42, v42-wt, SaCas9 and SaCas9-KKH. To evaluate the efficiency of v17-L at NNVRRN PAMs, 37 different endogenous sites were chosen. In addition, in the six non-NNNRRN PAMs, we selected 11 endogenous sites. Finally, we compare the activities of all the variants at 6 endogenous sites with NNNRRN PAMs.

2.3 As the authors are aware, the SaCas9 PAM is 6 nucleotides. This reviewer appreciates the desire for brevity and reducing the PAM to stating only positions 4-6, but in many cases this is confusing. The authors should make it abundantly clear what the nucleotides in positions 1-3 are. In many cases, the 3rd PAM position should be explicitly stated, since the context of the whole PAM is important for activity. The full PAM sequence should be included for all endogenous target sites, and the nucleotides in positions 1-3 should be included in the schematic of Fig. 1C (if the sequence is indeed consistent across all EYFP experiments).

A2.3: We thank the reviewer for this comment. We described the whole PAM with six positions in the revised manuscript. We listed the sequences of PAM sequences (1-6 positions) and the nucleotide right after the PAM (the 7th position) in the Supplementary Tables. To evaluate the preference at the 3rd PAM position, we generated a sequence logo by using the tested endogenous target sites with more than 5% mutation frequency generated by cCas9 v42 and SaCas9-KKH (Supplementary Fig. 5b). It is consistently with the previous report that A, C and G is preferred at 3rd position by SaCas9-KKH [2]. In addition, the nucleotides in positions 1-3 have been included in all figures.

2.4 Related to point #2, in supplementary Table 2, it looks like the authors are using a CCCNNN PAM for their EYFP experiments. Why did the authors choose a static PAM with a C in the 3rd position? Using a G in the 3rd position would have enabled a comparison to wild-type SaCas9, and the authors should therefore make it clear why they chose a C in the 3rd position. This is a major flaw in their experimental design that must be addressed.

A2.4: We thank the reviewer for this comment. We arbitrarily assigned triple cytosines at the first to third PAM positions because the SaCas9-KKH was reported to recognize NNNRRT PAM sites and the 3D structural study suggested that the 13-aa region is only responsible for the contact with 4th to 6th position of the PAM sequences [1,2]. We agree with

the reviewer for this comment that it is necessary to test the activities of Cas9 variants at sites with a G in the 3rd position. In the revised manuscript, we included the results of the EYFP reporter assay at PAMs with a C or G in the 3rd position, which allowed us to compare the activities of different Cas9 variants to the wildtype SaCas9 (Fig. 2c and Fig. 4a). In addition, we compared the activities of different variants across PAMs with A, C, G or T at the third position by the EYFP reporter assay (Supplementary Fig. 4 and Supplementary Fig. 9).

2.5 The authors need to be much more careful when making comparative statements to the KKH variant. For example, "These results suggested that cCas9 V42 had an enhanced PAM recognition capability than SaCas9-KKH" implies that the cV42 variant is more active than KKH on the PAMs that the KKH variant is known to target (NNNRRT). There are only two RRT PAMs in Supp. Fig. 3B, and KKH performs equal to cV42 on one site and almost ten-fold better on the other. The authors should use a word like "expanded" instead of enhanced, as the cV42 is not enhanced compared to KKH. Additionally, the authors should indicate in all of their comparisons to KKH which PAMs are expected to be targeted by KKH, and which are not (based on the NNNRRT PAM preference).

A2.5: We thank the reviewer for this comment. We carefully modified the corresponding text in the revised manuscript when describing the comparison between cCas9 v42 and SaCas9-KKH. We changed the title of our manuscript to "Engineer chimeric Cas9 to expand PAM recognition based on evolutionary information." We have classified the results of targeting endogenous sites into different groups of PAMs based on the SaCas9-KKH or SaCas9 preferences (Fig. 2d and Fig. 4b).

2.6 The manuscript would benefit from substantial English-language editing. There are numerous grammatical mistakes throughout (prominently in the title and in the abstract). Also, the title implies something different than what the study

delivers (since the variants aren't really enhanced), so the title should be revised accordingly.

A2.6: We thank the reviewer for this suggestion. We revised the title as following: "Engineer chimeric Cas9 to expand PAM recognition based on evolutionary information". LetPub (www.letpub.com) linguistic assistance was used to help us check writing and grammatical mistakes during the preparation of this manuscript. We have corrected the mistakes accordingly.

2.7 The authors should re-plot the KKH data beside the KKH(N968S) variant data in Fig. 3D. Also, the authors should probably name the KKH(N968S) variant as KSH, since they changed an amino acid that is contained within the KKH variant. Furthermore, the KSH variant data should be plotted in Fig. 3E, and also experiments should be performed with this variant for the equivalent sites in Fig. 3F and Supplementary Figure 4. From their assay data in Fig. 3D, the KSH variant looks like it should be the most active, so this is a notable omission not to include it in the most relevant experiments.

A2.7: We thank the reviewer for this suggestion. The mutation is at 986 not 968 which is not one of three mutations in SaCas9-KKH. To compare the activity of N986S and N986S-wt with other cCas9 variants, we tested the N986S and N986S-wt activities at 18 non-NNNRRN PAMs along with v21-R, v21-L, v21-R-wt, v21-L-wt, SaCas9 and SaCas9-KKH (Fig. 4a). Furthermore, we compared indel frequency induced by these variants across a panel of sites with NNVRRN or non-NNNRRN PAMs (Fig. 4b and Supplementary Fig. 11).

2.8 The on-target editing comparisons between KKH, V21R, and V21R-HF in Figure 4 should be expanded. At the one site examined, there is a clear loss of on-target activity, so the authors should compare the on-target activities on 10-12 on-target sites at a minimum if this is a major point of their manuscript (to justify including it as a main figure).

A2.8: We thank the reviewer for this suggestion. In Fig. 4b and 4c, we showed the activity of cCas9 v21-R-HF cross 11 endogenous target sites with non-NNNRRN PAMs compared with v21-R, v21-L, N986S, v21-R-wt, v21-L-wt, N986S-wt, SaCas9 and SaCas9-KKH. We also tested the activities of cCas9 v21-R-HF along with other variants at another six sites with NNVRRN (Supplementary Fig. 11). Furthermore, we measured the activities of v21-R-HF at two groups of on-target and off-target sites (Supplementary Fig. 10). We observed that v21-R-HF displayed significantly decreased rates of mutagenesis at two out of three endogenous off-target sites containing one point-mutation in the spacer sequences when directed by either wildtype gRNA or optimized gRNA-2. Totally, these results showed that v21-R-HF can generate indel at endogenous sites in human cells with a higher specificity.

2.9 In the text, the rationale for switching from chimeric variants to orthologs is not clear, and the statement in the manuscript "Next, we tested the gene editing activity of these SaCas9 orthologs in mammalian cells." does not indicate why. In Supplementary Figures 6B and 6C, it looks like the KKH variants of O32 and O35 actually decrease activity compared to V32 and V35. The authors should include experiments to compare the activities of wild-type O32 and O35 in Supplementary Figure 6D to O32-KKH and O35-KKH. Also, what crRNA/tracrRNAs are used in these experiments, are they the optimal one for the variant/ortholog?

A2.9: We thank the reviewer for this suggestion. As the activities of orthologs (SshCas9 and SlCas9) were weak and unrelated to the main idea of the chimeric Cas9, we excluded the results of SshCas9, SlCas9, cCas9 v32 and v35 in the revised manuscript.

2.10 In Supplementary Figure 2, the gRNA scaffold optimization is very interesting. The authors should perform identical experiments with wild-type SaCas9 to determine if they observe a concomitant increase in activity and targeting range with the optimized sgRNA scaffold. Furthermore, in Figure 2B and Supplementary

Figure 2C, where are the results for optimized sgRNA-1? And is optimized sgRNA-1 the previously described one from ref. 25? This should be clarified.

A2.10: We thank the reviewer for this suggestion. We performed the EYFP reporter assay to test whether there is a concomitant increase in activity and targeting range with optimized gRNA scaffold for SaCas9 and SaCas9-KKH (Supplementary Fig. 2). We found that the activity of SaCas9-KKH were increased but the targeting range remained approximately the same (Supplementary Fig. 2c). We further compared the activities of SaCas9 and SaCas9-KKH at CCGRRN with a guanine at the third position and CCCRRN PAMs with a cytosine at the third position, guided with wildtype gRNA or optimized gRNA-2. Similarly, we observed increased activity at CCGRRV PAMs by SaCas9 and CCSRRV PAMs (S=C or G) by SaCas9-KKH when directed with the optimized gRNA-2 (Supplementary Fig. 2d).

In addition, as shown in Supplementary Fig. 10, we further compared the activities of v21-R and v21-R-HF at two groups of on-target and off-target sites guided by either the wildtype gRNA or optimized gRNA-2.

We apologized for the omission of the description of the optimized gRNA-1. We described the result of optimized gRNA-1 in our previous publication [3]. We added the reference and described in the revised Supplementary Figure 2 as following: "... The optimized gRNA-1 is developed previously…".

Minor comments:

2.11 It would be very helpful if the authors could make the order of orthologs consistent between Figs. 1B and 1D.

A2.11: We thank the reviewer for this suggestion. Although the same sequence would be helpful for the comparison, as the order is determined by the sequence similarity (Fig. 1b) and cluster of the activities at different PAMs (Fig. 1d), we still kept the original order.

2.12 The "cCas9 V17K and cCas9 V17L" nomenclature is confusing, and implies a

mutation at Valine 17. Perhaps the authors can use more complete names (v17-I991L) or consider making the "V" lowercase (v17-L)?

A2.12: We thank the reviewer for this suggestion. We have corrected the nomenclature in the revised manuscript. The chimeric Cas9 based on SaCas9-KKH is named as v+number. The chimeric Cas9 based on wildtype SaCas9 is named as v+number+"-wt". For the addition mutation, we added the label of the mutation like v17-L.

2.13 In Supplementary Figure 2A, the authors should indicate from which strain each crRNA is derived from.

A2.13: We thank the reviewer for careful reading of our manuscript. It has been corrected in the revision manuscript. In Supplementary Fig. 2a, the strain name of the original source of each crRNA was added in the left.

2.14) What PAMs do the other orthologs in this study naturally target (can the authors predict bioinformatically from the crRNA sequences in the endogenous CRISPR locus)?

A2.14: We thank the reviewer for this suggestion. We chose the Staphlococus Warneri from which the v21 variant was derived for further analysis. We first predicted the CRISPR loci in the genome of Staphlococus Warneri by CRISPRfinder webserver [4]. As shown in Response Fig. 1, the natural target was further predicted by CRISPRTarget webserver [5]. Interestingly, these natural targets included the new non-NNNRRN PAM sites discovered in our assays of cCas9 v21 (Fig. 3 and Fig. 4).

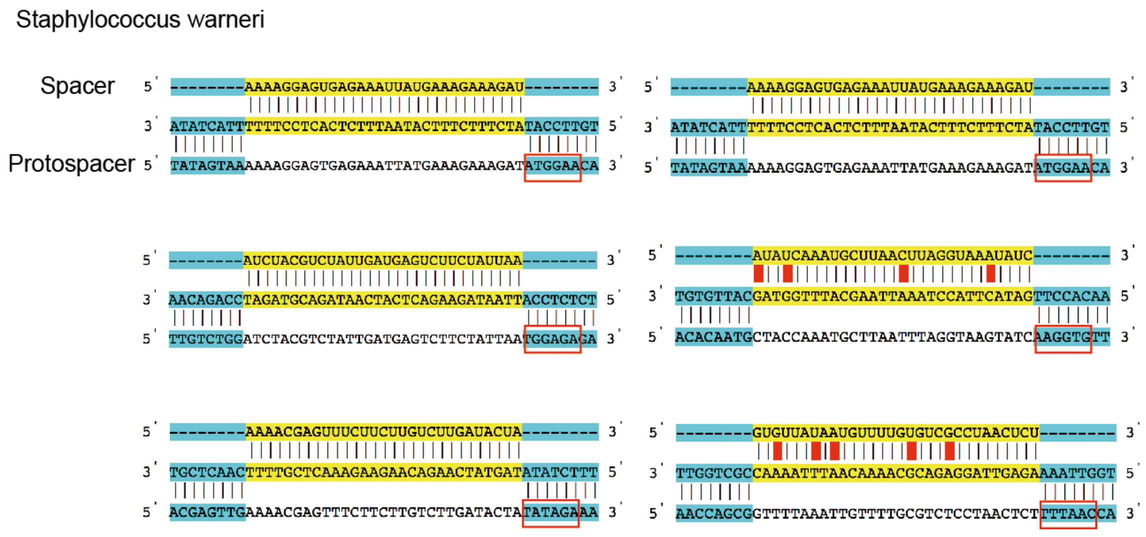

Response Figure 1| Prediction of the PAM in natural targets.

The red box indicates the predicted PAM sequences. The red thick line pointed to the mismatch. The upper sequences are the sequence of spacers from the CRISPR loci and the lower sequences are predicted targeted sequences from phage or plasmid in bacteria.

2.15 Why do the authors wait 8 days before extracting genomic DNA and performing the T7E1 assay? Almost all other genome editing studies use a 3-day time point.

A2.15: We thank the reviewer for this comment. To realize more sufficient genome editing, we chose the 5-day time point for endogenous gene editing assay in the revised manuscript.

2.16 I believe that the 6th position of the PAM is mislabeled in Figure 3, as order of PAMs between the plots of Figure 2B-2D compared to 3B-3D (in the 6th position, ACGT vs. ATCG, respectively) changes (and doesn't seem correct in Figure 3).

A2.16: We thank the reviewer for careful reading of our manuscript. We apologized for this mistake. It has been corrected in the revised Fig. 3.

2.17 In Supplementary Figure 5, the authors should perhaps generate the alignment with SaCas9 instead of SaCas9-KKH and simply indicate the positions where the KKH substitutions are found.

A2.17: We thank the reviewer for this suggestion. As the activities of orthologs (SshCas9 and SlCas9) were weak and unrelated to the main idea of the chimeric Cas9, we excluded the results of SshCas9, SlCas9, cCas9 v32 and v35 in the revised manuscript.

2.18 In Supplementary Table 2, it would be helpful for the authors to indicate important parts of the polypeptide sequences (for example, what sequence is the NLS, VPR, etc.)

A2.18: We thank the reviewer for careful reading of our manuscript. We have provided the annotaton of the sequences of the NLS and VPR domains in Supplementary Table 8.

2.19 Certain statements are lacking citations, such as: “By using our previous EYFP fluorescence reconstitution assay in HEK293FT cells”

A2.19: We thank the reviewer for careful reading of our manuscript. We apologized for this omission. The reference was added in the corresponding text.

References

1. Nishimasu, H. *et al*. Crystal Structure of Staphylococcus aureus Cas9. *Cell* **162,** 1113–1126 (2015).
2. Kleinstiver, B. P. *et al*. Broadening the targeting range of Staphylococcus aureus CRISPR-Cas9 by modifying PAM recognition. *Nat. Biotechnol.* **33,** 1–7 (2015).
3. Ma, D., Peng, S., Huang, W., Cai, Z. & Xie, Z. Rational Design of Mini-Cas9 for Transcriptional Activation. *ACS Synth. Biol.* **7,** 978–985 (2018).
4. Grissa, I., Vergnaud, G. & Pourcel, C. CRISPRFinder: a web tool to identify clustered regularly interspaced short palindromic repeats. *Nucleic Acids Res.* **35,** W52-7 (2007).

5. Biswas, A., Gagnon, J. N., Brouns, S. J. J., Fineran, P. C. & Brown, C. M. CRISPRTarget: Bioinformatic prediction and analysis of crRNA targets. *RNA Biol*. **10,** 817–827 (2013).

REVIEWERS' COMMENTS:

Reviewer #1 (Remarks to the Author):

Overall, the authors have done a good job addressing the referees' concerns and I support publication of the revised manuscript, following a careful stylistic revision to remove hyperbole and further improve grammar and clarity (which is already quite improved relative to the initial version).

Reviewer #2 (Remarks to the Author):

The authors are commended for performing NGS and additional experiments. However, major concerns remain about the on-target activities (and therefore utility) of the engineered variants. The fact remains that the genome editing activities of the v42 variant are substandard, which calls into question the usefulness of these variants for genome editing in human cells. On average, the mean activities of the v42 variant (~10% mean indels by NGS) across what the authors deem as targetable PAMs are ~2-fold less active than the engineered SaCas9-KKH variant on the NNNRRT PAMs (~20% mean indels by NGS; compare right-most panel of Figure 2d to the other four panels). This activity discrepancy is further highlighted by their EYFP reporter data shown in Figure 3b when comparing SaCas9-KKH to their v21 variants, where again SaCas9-KKH appears to exhibit 2-to-4-fold higher activities. The authors own statement: “We observed that v17-L displayed about half of the sites showing higher than 5% indels with a mean mutagenesis frequency of 9.5%” elaborates this low activity; this level of activity is barely higher than the ~6-7% editing by SaCas9-KKH on sites that the KKH variant is known NOT to target (see Sup Fig. 3b). These levels of editing activity are not consistent with the language used in the text, including the statement “… will be highly valuable for basic research and biomedical applications…”.

Overall, while the strategy of PAM engineering is interesting and somewhat novel, these variants do not constitute a major advance in terms of activity (and in fact, are about ~2-fold lower in activity compared to the SaCas9-KKH variant published over 3 years ago). A mean 9.5% editing activity in human cells will not be useful to the genome editing community.

Additionally, in the future, it would be helpful for the authors to track changes, or provide a marked up version of their revised manuscript with changes highlighted in different colored/strike-through text.

REVIEWERS' COMMENTS:

Reviewer #1 (Remarks to the Author):

Overall, the authors have done a good job addressing the referees' concerns and I support publication of the revised manuscript, following a careful stylistic revision to remove hyperbole and further improve grammar and clarity (which is already quite improved relative to the initial version).

R: Thanks a lot for your suggestions. We stylized the manuscript in the editor's direction and remove the hyperbole words like "novel", "new", etc. In addition, we improve the grammar question, especially the tense problem.

Reviewer #2 (Remarks to the Author):

The authors are commended for performing NGS and additional experiments. However, major concerns remain about the on-target activities (and therefore utility) of the engineered variants. The fact remains that the genome editing activities of the v42 variant are substandard, which calls into question the usefulness of these variants for genome editing in human cells. On average, the mean activities of the v42 variant (~10% mean indels by NGS) across what the authors deem as targetable PAMs are ~2-fold less active than the engineered SaCas9-KKH variant on the NNNRRT PAMs (~20% mean indels by NGS; compare right-most panel of Figure 2d to the other four panels). This activity discrepancy is further highlighted by their EYFP reporter data shown in Figure 3b when comparing SaCas9-KKH to their v21 variants, where again SaCas9-KKH appears to exhibit 2-to-4-fold higher activities.
The authors own statement: "We observed that v17-L displayed about half of the sites showing higher than 5%

indels with a mean mutagenesis frequency of 9.5%" elaborates this low activity; this level of activity is barely higher than the ~6-7% editing by SaCas9-KKH on sites that the KKH variant is known NOT to target (see Sup Fig. 3b). These levels of editing activity are not consistent with the language used in the text, including the statement "… will be highly valuable for basic research and biomedical applications…".

Overall, while the strategy of PAM engineering is interesting and somewhat novel, these variants do not constitute a major advance in terms of activity (and in fact, are about ~2-fold lower in activity compared to the SaCas9-KKH variant published over 3 years ago). A mean 9.5% editing activity in human cells will not be useful to the genome editing community.

Additionally, in the future, it would be helpful for the authors to track changes, or provide a marked up version of their revised manuscript with changes highlighted in different colored/strike-through text.

R: Thanks a lot for your suggestions. Although the v42 variant expands the PAM preference in NNVRRV, the activity is lower compared with SaCas9-KKH at the NNNRRT PAM sites. We added a few of sentences in the Discussion Part as "…Although cCas9 variants displayed expanded ability in non-canonical sites, we found these developed cCas9 variants showed decreased activities in previous canonical PAMs of SaCas9-KKH. For example, the cCas9 v42 generates lower efficiency in sites with NNNRRT PAMs, in line with the previous finding that SaCas9-KKH is weaker than wildtype SaCas9 at PAM sites with guanine at the third position. In general, one of possible explanation is that the specific recognition and broad recognition is contradicted with each other due to the nature of protein-DNA interaction.…" in Page 9-10.

In addition, we delete the "highly" in the statement "… will be highly valuable for basic research and biomedical applications…" to avoid hyperbole.
