# Supplementary material for: Engineer chimeric Cas9 to expand PAM recognition based on evolutionary information
Source: Nat Commun. 2019 Feb 4;10:560. doi: 10.1038/s41467-019-08395-8 (PMC6361995; doi:10.1038/s41467-019-08395-8)
Supplement: Supplementary file 2 — Peer Review File [file 41467_2019_8395_MOESM2_ESM.pdf]

nature research

Corresponding author(s): Zhen Xie

Reporting Summary

Nature Research wishes to improve the reproducibility of the work that we publish. This form provides structure for consistency and transparency in reporting. For further information on Nature Research policies, see Authors & Referees and the Editorial Policy Checklist.

Statistical parameters

When statistical analyses are reported, confirm that the following items are present in the relevant location (e.g. figure legend, table legend, main text, or Methods section).

n/a	Confirmed	
☐	☒	The exact sample size (n) for each experimental group/condition, given as a discrete number and unit of measurement
☐	☒	An indication of whether measurements were taken from distinct samples or whether the same sample was measured repeatedly
☐	☒	The statistical test(s) used AND whether they are one- or two-sided<br>*Only common tests should be described solely by name; describe more complex techniques in the Methods section.*
☒	☐	A description of all covariates tested
☐	☒	A description of any assumptions or corrections, such as tests of normality and adjustment for multiple comparisons
☐	☒	A full description of the statistics including central tendency (e.g. means) or other basic estimates (e.g. regression coefficient) AND variation (e.g. standard deviation) or associated estimates of uncertainty (e.g. confidence intervals)
☐	☒	For null hypothesis testing, the test statistic (e.g. F, t, r) with confidence intervals, effect sizes, degrees of freedom and P value noted<br>*Give P values as exact values whenever suitable.*
☒	☐	For Bayesian analysis, information on the choice of priors and Markov chain Monte Carlo settings
☒	☐	For hierarchical and complex designs, identification of the appropriate level for tests and full reporting of outcomes
☒	☐	Estimates of effect sizes (e.g. Cohen's d, Pearson's r), indicating how they were calculated
☐	☒	Clearly defined error bars<br>*State explicitly what error bars represent (e.g. SD, SE, CI)*

Our web collection on statistics for biologists may be useful.

Software and code

Policy information about availability of computer code

Data collection	A custom perl script was used.
Data analysis	A custom perl script was used to analyze the indel frequency.

For manuscripts utilizing custom algorithms or software that are central to the research but not yet described in published literature, software must be made available to editors/reviewers upon request. We strongly encourage code deposition in a community repository (e.g. GitHub). See the Nature Research guidelines for submitting code & software for further information.

Data

Policy information about availability of data

All manuscripts must include a data availability statement. This statement should provide the following information, where applicable:

- Accession codes, unique identifiers, or web links for publicly available datasets
- A list of figures that have associated raw data
- A description of any restrictions on data availability

plasmids information was listed in Supplementary Information. There are no restrictions on data availability. Next-generation sequencing data for detection indel frequency of the specific sites are available through NCBI Sequence Read Archive (PRJNA513032) [https://www.ncbi.nlm.nih.gov/bioproject/PRJNA513032]. The source data underlying Figs 1d, 2b-d, 3b-c, 4a-c and Supplementary Figs 2c-d, 3-11 are provided as a Source Data file. All the other data supporting the findings of

this study within the article and its supplementary information files are available from the corresponding author upon reasonable request.

Field-specific reporting

Please select the best fit for your research. If you are not sure, read the appropriate sections before making your selection.

☒ Life sciences ☐ Behavioural & social sciences ☐ Ecological, evolutionary & environmental sciences

For a reference copy of the document with all sections, see nature.com/authors/policies/ReportingSummary-flat.pdf

Life sciences study design

All studies must disclose on these points even when the disclosure is negative.

Sample size	Experiments were performed in biological triplicate n=3 unless otherwise noted.
Data exclusions	No data was excluded.
Replication	All attempts at replication were successful, and standard deviations were within expected ranges.
Randomization	Different cell passages were used for each biological replicate.
Blinding	Not applicable, as samples were processed identically through standard and in some cases automated procedures (DNA sequencing, transfection, DNA isolation) that should not bias outcomes.

Reporting for specific materials, systems and methods

Materials & experimental systems

n/a	Involved in the study	
☒	☐	Unique biological materials
☒	☐	Antibodies
☐	☒	Eukaryotic cell lines
☒	☐	Palaeontology
☒	☐	Animals and other organisms
☒	☐	Human research participants

Methods

n/a	Involved in the study	
☒	☐	ChIP-seq
☐	☒	Flow cytometry
☒	☐	MRI-based neuroimaging

Eukaryotic cell lines

Policy information about cell lines

Cell line source(s)	HEK293FT (Life)
Authentication	Cells were authenticated by the supplier.
Mycoplasma contamination	HEK293FT cells tested negative for mycoplasma.
Commonly misidentified lines (See ICLAC register)	No commonly misidentified cell lines were used.

Flow Cytometry

Plots

Confirm that:

☒ The axis labels state the marker and fluorochrome used (e.g. CD4-FITC).

☒ The axis scales are clearly visible. Include numbers along axes only for bottom left plot of group (a 'group' is an analysis of identical markers).

☒ All plots are contour plots with outliers or pseudocolor plots.

☒ A numerical value for number of cells or percentage (with statistics) is provided.

Methodology

Sample preparation	Cells were trypsinized 3 days after transfection and centrifuged at 300 g for 7 min at 4 °C. The supernatant was removed, and the cells were resuspended in 1× PBS that did not contain calcium or magnesium.
Instrument	LSRFortessa (BD Biosciences)
Software	Fortessa flow analyzer (BD Biosciences) was used for fluorescence-activated cell sorting (FACS) analysis
Cell population abundance	HEK293FT EBFP2+ in trans cells were typically over 50% of the population.
Gating strategy	Negative control (unstained) and fluorophore-positive cells were used to establish gates for each cell type.

☒ Tick this box to confirm that a figure exemplifying the gating strategy is provided in the Supplementary Information.
